# Improving Protein Fold Recognition by Deep Learning Networks

**DOI:** 10.1038/srep17573

**Published:** 2015-12-04

**Authors:** Taeho Jo, Jie Hou, Jesse Eickholt, Jianlin Cheng

**Affiliations:** 1 Department of Computer Science, University of Missouri, Columbia, MO 65211, USA; 2 Department of Biological Chemistry, University of Michigan, Ann Arbor, MI, 48109, USA; 3 Department of Computer Science, Central Michigan University, Mount Pleasant, MI 48859, USA

## Abstract

For accurate recognition of protein folds, a deep learning network method (DN-Fold) was developed to predict if a given query-template protein pair belongs to the same structural fold. The input used stemmed from the protein sequence and structural features extracted from the protein pair. We evaluated the performance of DN-Fold along with 18 different methods on Lindahl’s benchmark dataset and on a large benchmark set extracted from SCOP 1.75 consisting of about one million protein pairs, at three different levels of fold recognition (i.e., protein family, superfamily, and fold) depending on the evolutionary distance between protein sequences. The correct recognition rate of ensembled DN-Fold for Top 1 predictions is 84.5%, 61.5%, and 33.6% and for Top 5 is 91.2%, 76.5%, and 60.7% at family, superfamily, and fold levels, respectively. We also evaluated the performance of single DN-Fold (DN-FoldS), which showed the comparable results at the level of family and superfamily, compared to ensemble DN-Fold. Finally, we extended the binary classification problem of fold recognition to real-value regression task, which also show a promising performance. DN-Fold is freely available through a web server at http://iris.rnet.missouri.edu/dnfold.

Proteins are key functional units in living organisms and are involved in many biological processes in the cell. As a protein’s three dimensional structure largely determines its function, there is great interest in methods to determine or predict a protein’s tertiary structure. Several experimental methods including X-ray crystallography, NMR spectroscopy, and electron microscopy have been used to determine protein structure. However, due to the significant cost and time for using those methods, the number of proteins with known structure(s) is significantly smaller than the number of known protein sequences (i.e. by a factor of approximately 200) and the sequence-structure gap is still increasing^1^,^2^. Therefore, the construction of computational approaches and tools to predict a protein’s three dimensional structure from its sequence is an important problem not only for understanding the relationship between protein structure and function^3^, but also for advancement in protein-based biotechnologies and drug discovery^4^.

An important task in protein structure prediction is to identify proteins that have similar tertiary structures (from among those that have already been determined experimentally). By identifying such proteins, their structures can be used as a template to model the unknown structure of another protein. The process for identifying these structurally similar proteins is called fold recognition and forms the basis of the fold recognition problem. This task grows increasingly difficult when the sequence level identity between two protein sequences (i.e., a query protein without known structure and a template protein with known structure) is very low.

In this work, we applied a new, powerful classification method – Deep-learning Networks (DNs) to tackle the fold recognition problem. In order to apply DNs to the protein fold recognition problem, we treated it as a binary classification problem (i.e., predicting whether a protein pair shares the same structural fold based on the pairwise similarity features between them as done elsewhere in the literature^5^,^6^). DNs are a new set of machine learning algorithms to map a set of initial input features into multiple layers of higher-level representations through unsupervised learning in order to improve the prediction accuracy for the final supervised learning (e.g. classification) task. DNs have been applied to hand writing recognition and face recognition achieving state-of-art performance. Most recently, DNs were introduced to the field of bioinformatics to predict protein residue disorder and residue-residue contacts^7^,^8^,^9^, which delivered the best residue-residue contact prediction performance in the 10th Critical Assessment of Techniques for Protein Structure Prediction (CASP) in 2012. To the best of our knowledge, this work is the first attempt of using DNs for the fold recognition problem.

Specifically, our method, DN-Fold, predicts if two proteins are from the same fold, taking as input pairwise protein features such as sequence or family information, sequence alignment, sequence–profile alignment, profile–profile alignment, structural features, and structure-seeded profiles. We compared the performance of DN-Fold on the standard (LINDAHL dataset)^10^ along with our previous method, RF-Fold^11^, and 17 other methods such as PSI-BLAST^12^, HMMER^13^, SAM-T98^14^, SSHMM^15^, THREADER^16^, FUGUE^17^, SPARKS^18^, SP3^19^, HHpred^20^, FOLDpro^5^, SP4^21^, SP5^22^, RAPTOR^23^, SPARKS-X^24^, and BoostThreader^25^. DN-Fold achieved a performance approaching those methods that represent the state-of-the-art at all three levels of difficulty (i.e., protein family, superfamily, and fold). In addition to DN-Fold, which is an ensemble method comprised of several DN models, we constructed a single model method which we call DN-FoldS and a single linear regression model which we call DN-FoldR.

## Results and discussion

**Figure 1:**
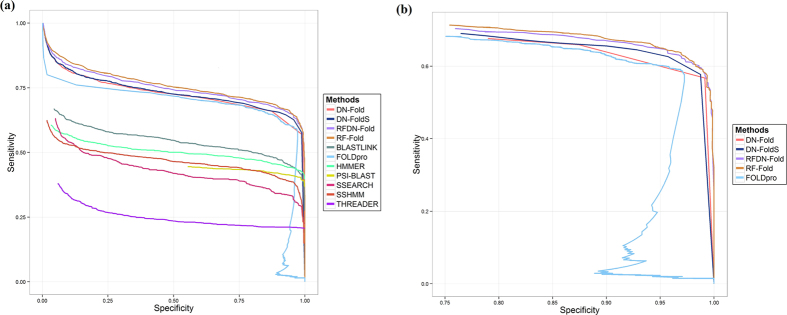
(**a**) The specificity - sensitivity plot of classification results at family level. (**b**) Plotting the performance at family level in the high specificity range ([0.75:1]).

**Figure 2:**
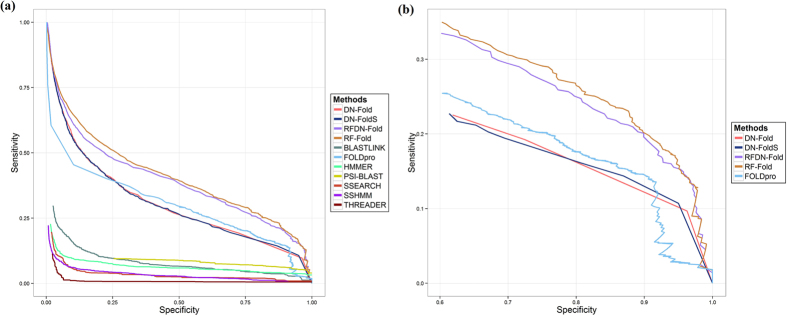
(**a**) The specificity - sensitivity plot of classification results at super-family level. (**b**) Plotting the performance at super-family level in the high specificity range ([0.6:1]).

**Figure 3:**
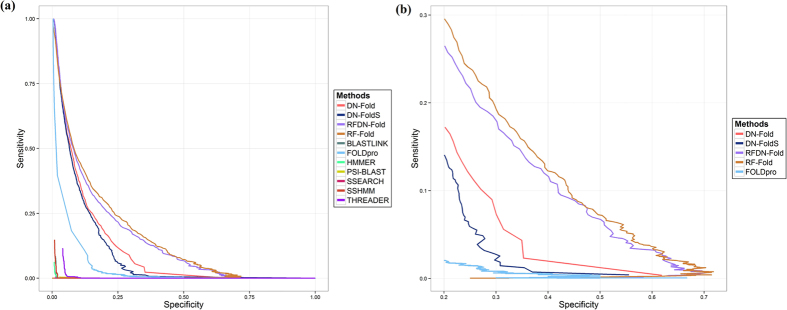
(**a**) The specificity - sensitivity plot of classification results at fold level. (**b**) Plotting the performance at fold level in the high specificity range ([0.2:0.8]).

As an initial attempt to apply DNs to the protein fold recognition problem, a variety of DN architectures and combinations of hyperparamenters were evaluated. The optimal DN configuration was used to predict fold membership for a pair of proteins at fold, superfamily and family levels and this initial classifier was termed DN-FoldS. We compared this approach with several fold recognition methods (RF-Fold, FOLDpro, THREADER, SSHMM, SSEARCH, BLASTLINK, HMMER and PSI-BLAST) on the LINDAHL dataset using specificity-sensitivity plots at fold, superfamily and family levels (Figs 1(a), 2(a) and 3(a)) as done elsewhere in the literature^10^. In these plots, the *specificity* is defined as the percentage of predicted positives that are true positives, and the *sensitivity* is defined as the percentage of true positives that are predicted as positives. We applied the same feature extraction algorithm with FOLDpro (a method based on support vector machines), and the plots show that DN-FoldS performed better than FOLDpro at the family and fold levels in the lower and higher ranges of specificity. To show the sensitivity for the range of higher specificity, we made three additional figures (Figs 1(b), 2(b) and 3(b)) plotting the performance in the high specificity range. Table 1 compares the sensitivity of DN-FoldS against 18 other fold recognition methods tested on the LINDAHL dataset at the family, superfamily and fold levels, for the Top 1 and Top 5 predicted template folds respectively. Here, the sensitivity (success rate) is defined as the percentage of query proteins having at least one correct template fold ranked first, or within the Top 5, respectively^10^

**Figure 4:**
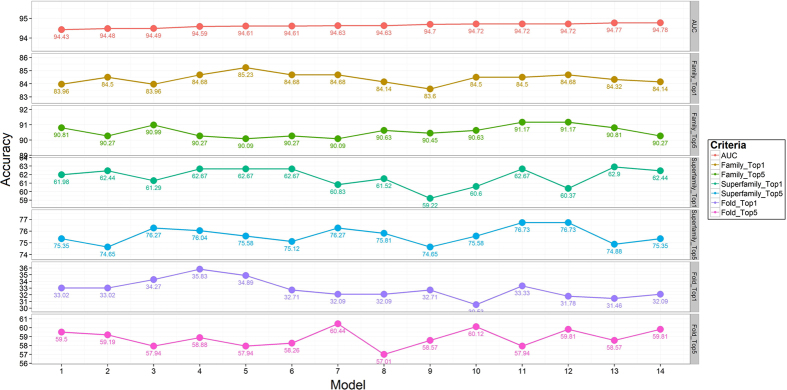
The prediction accuracy of 14 deep learning architectures on the training dataset at the levels of family, superfamily and fold at the Rank 1 and Rank 5, and were sorted by AUC from lowst to highest.

**Figure 5:**
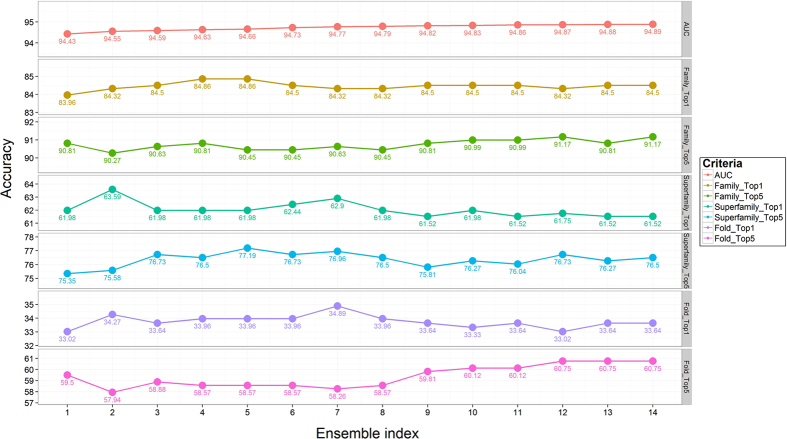
The prediction accuracy of 14 deep learning ensembles on the training dataset at the levels of family, superfamily and fold at the Rank 1 and Rank 5. The ensemble index was defined by the number of models in each ensemble. Each ensemble was evaluated by AUC.

In spite of an extensive parameter search and optimization process, the performance of DN-FoldS continued to lag behind RF-Fold. This was attributed, in part, to the fact the RF-Fold is an ensemble approach and DN-FoldS is comprised of a single classifier. Ensemble approaches typically generalize better and have lower variance in average performance across different datasets. As a result, an ensemble approach termed DN-Fold was constructed from a pool of candidate deep networks. To construct DN-Fold, 10-fold cross validation was applied on each architecture such that ten deep networks were trained from the 10-folds of data and the results of each fold were combined into the final prediction. A number of different deep network architectures were used and evaluated in terms of the AUC value. Figure 4 represents the performance of 14 deep network architectures on three kinds of categories, in which the models from left to right were sorted based on AUC. Within the level of superfamily and fold, models have relatively large variation, compared to steady trend at the level of family. We evaluated 14 different ensembles which integrated the models sequentially from lowest AUC to highest AUC, ranging from 1 model to 14 models. Simple averaging was applied to ensemble prediction of different models. Figure 5 reveals that model ensembling provides improved performance through all three levels, which also reduce the bias and variance.

We compared our novel method DN-Fold and a new meta-method RFDN-Fold (a combination of deep learning networks and random forests) with the other fold recognition methods (RF-Fold, FOLDpro, THREADER, SSHMM, SSEARCH, BLASTLINK, HMMER and PSI-BLAST) as previously described on the LINDAHL dataset using specificity-sensitivity plots at fold, superfamily and family levels (Figs 1(a), 2(a) and 3(a)) as in the work by Lindahl and Elofsson^10^. The sensitivity of DN-Fold and RFDN-Fold was also calculated and shown in Table 1. The last five rows in the table show that DN-Fold and RFDN-Fold performed better than FOLDpro^5^ in all but one case (i.e. Top 1 predictions at the family level). At the family level for Top 1 predictions, the success rate (sensitivity) of FOLDpro (85.0%) was slightly better than DN-Fold (84.5%), and RFDN-Fold (84.7%). At the superfamily level, the sensitivity of DN-Fold for the Top 1 or Top 5 predictions is 61.5% and 76.5%, about 6% higher than FOLDpro. In the case of RFDN-Fold, at the superfamily level, the sensitivity for the Top 1 or Top 5 predictions is 65.7% and 78.8%, 9~10% higher than FOLDpro. At the fold level, the sensitivity of DN-Fold for the Top 1 or Top 5 predictions is 33.64% and 60.7%, about 8~11% higher than FOLDpro. RFDN-Fold yields the largest improvement for Top 1 predictions at the fold level, which is 11.2% higher than FOLDPro’s. The sensitivity of RFDN-Fold for the Top 5 predictions is 61.7%, about 13.4% higher than FOLDpro’s.

When comparing the success rate of DN-Fold and RFDN-Fold for the Top 1 and Top 5 predictions, DN-Fold and RFDN-Fold also performed better than many of the other methods in Table 1, and competitive with state-of-the-art methods such as RF-Fold, RAPTOR, SPARKS-X, and BoostThreader. In particular, DN-Fold performed comparably to RF-Fold at the family and superfamily levels, and RFDN-Fold slightly outperformed or was nearly on par with RF-Fold and DN-Fold. Compared with SPARKS-X, DN-Fold and RFDN-Fold were less accurate at the fold level, but more accurate at the family and superfamily levels. Compared with BoostThreader, DN-Fold and RFDN-Fold were less accurate for Top 1 predictions at the three levels, but more accurate for Top 5 predictions at all three levels.

To determine if our method is prone to over-fitting and to see if our method is applicable to larger databases, we evaluated it on an independent dataset extracted from the SCOP(v1.75). This dataset was called SCOP_TEST. Table 2 shows the sensitivity on SCOP_TEST at the three levels for the Top 1 prediction or Top 5 predictions for query proteins. On this dataset, DN-Fold competes competitively against RF-Fold, and in all cases but the Top 5 at Family level and Top 1 at the superfamily level, RFDN-Fold shows improved results when compared with DN-Fold and RF-Fold at the fold level. For more detailed results of this comparison, consult the supplemental documentation. In this documentation, we provide a table for each method and each level which contains the name of protein domain, its identifier in SCOP, the length of its sequence, the number of sequences paired with each query sequence in each category and the final prediction results acquired by three methods. Each template sequence in Rank 1 and Rank 5 is linked with its SCOP id to compare with its query sequence. The table provides an intuitive way to understand the performance of the fold recognition methods. Complete information of our test dataset including benchmark results can be viewed at http://iris.rnet.missouri.edu/dnfold/data/model_evaluation.

**Figure 6:**
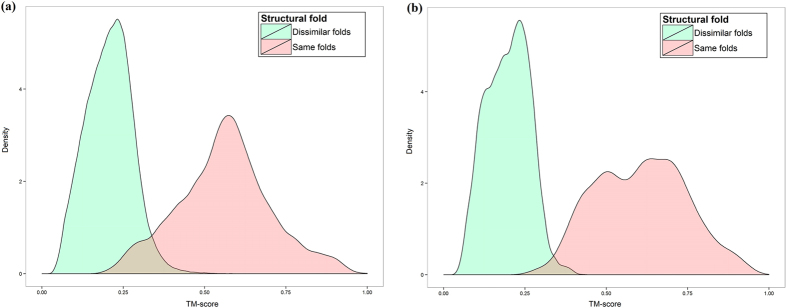
(**a**) The distribution of TM-score for protien pairings from the Foldpro dataset along with fold membership (i.e., if the protiens in the pair belong to the same fold). (**b**) The distribution of TM-score for protien pairings from the SCOP_TEST datasets along with fold membership.

**Figure 7:**
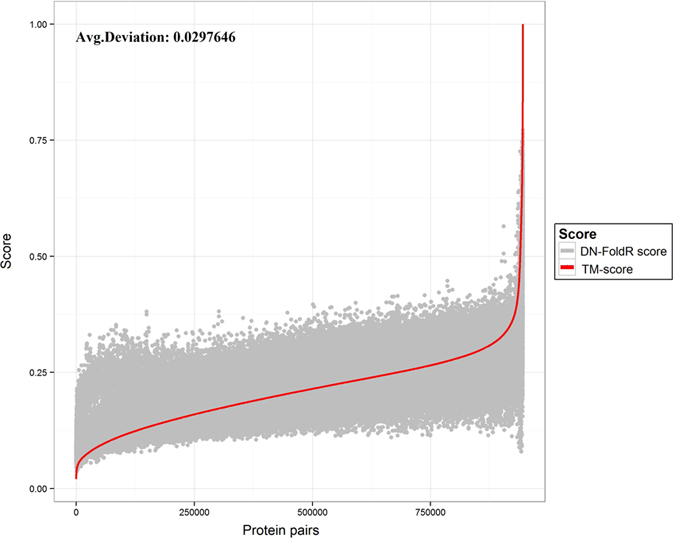
The comparison of TM-score predicted by DN-FoldR and true TM-score between protein pairs on FOLDpro dataset.

**Figure 8:**
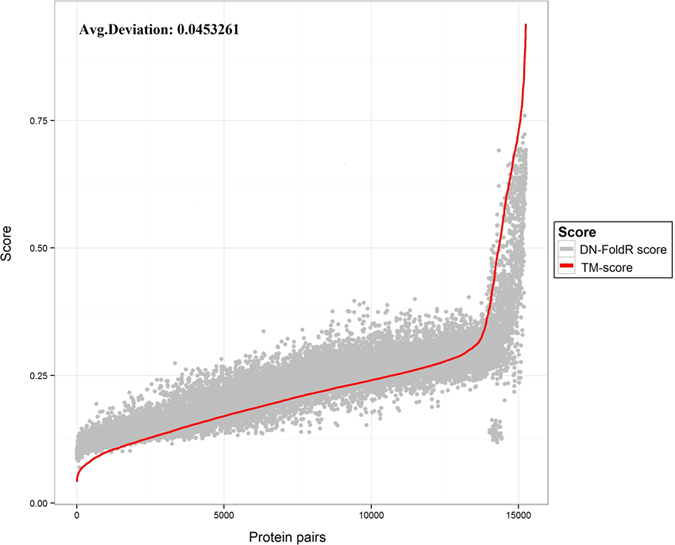
The comparison of TM-score predicted by DN-FoldR and true TM-score between protein pairs on SCOP_TEST dataset.

In addition to DN-Fold which is a binary classifier based on a DN, a linear regression model was also developed and named DN-FoldR. Development of this method was spurred for two reasons. First, the fold definition is binary and arbitrary compared to a continuously distributed structural space. The arbitrary nature of fold classification could be a limiting factor for performance. Second, it was noted that many model pairings had similar score in terms of the TM-score but had different classifications. This is illustrated in Fig. 6(a),(b). Particularly for TM-scores in the range of 0.30 to 0.50, there was significant overlap between pairs that did or did not belong to the same fold. This indicates that for protein pairings with significant structural differences, predicting fold membership may be difficult but it may still be possible to predict a secondary value which could be used to infer fold membership in some cases (e.g., if the predicted TM-score was above a particular threshold then the prediction for the pair would be same fold). The predicted real value could also be used to rank models and predict fold membership as it was done with the binary classifier. Figure 7 represents the deviation between the TM-score predicted by DN-FoldR and true TM-score on the LINDAHL dataset, and the averaged deviation is 0.029. Figure 8 shows the TM-score predicted by DN-FoldR and true value on the SCOP_TEST dataset, with given averaged deviation of 0.045. Table 1 reports the benchmark results of DN-FoldR on the LINDAHL dataset. At the family level, the sensitivity of DN-FoldR was 82.3% and 88.3% in Rank 1 and Rank 5, which exceeds the performance of SP5, SP4, HHpred, Fugue, THREADER, SSHMM, SSEARCH, BLASTLINK, SAM-T98, HMMER and PSI-BLAST. DN-FoldR also shows better performance than FOLDpro at the level of superfamily and fold, with 56%, 71% for the Top 1 prediction, and 27.4%, 57.3% for the Top 5 prediction. This result provides a new approach to the fold recognition problem in which a real value representing the on the continuously distributed structural space is learned from information such as sequence/profile alignment and structural information.

## Conclusions

In this work, we have constructed and evaluated three approaches (DN-Fold, DN-FoldS, DN-FoldR) to predict protein folds by using deep learning networks. The newly proposed method, DN-Fold, performed better than FOLDpro on a large standard benchmark dataset. When compared with 18 other methods evaluated on the same benchmark, DN-Fold was generally ranked among the top methods. Furthermore, combining DN-Fold with another machine learning-based fold recognition method (RF-Fold) shows good accuracy for fold recognition. While not advancing the state-of-the-art, the performance of DN-Fold was competitive, placing the approach among the top five methods at each level and demonstrates the effectiveness of applying the new deep learning technique to the fold recognition problem. DN-FoldR also extended the binary classification problem of fold recognition to a real-value regression task, which also show a promising performance, especially at the level of superfamily and fold.

## Methods

### Datasets and Feature Extraction

The principle dataset for this study is that used to train FOLDpro, a fold recognition program developed by Cheng and Baldi^5^, and originally developed by Lindahl and Elofsson^10^. This dataset consists of 976 proteins from the SCOP dataset (version 1.37)^6^ and was constructed such that the sequence identity between of any pair of proteins was <40%. Each protein was then paired with every other protein for an all-against-all comparison resulting in 951,600 (i.e., 976 * 975) protein pairs. Of these pairings, 555 shared the same family, 434 shared the same superfamily and 321 shared the same fold. In order to further assess the robustness of DN-Fold method, we checked how effectively our method could be applied to a new dataset that is independent from the LINDHAL dataset. Such inspection can largely determine if our method is prone to over-fitting and if our method is suitable for application on independent datasets. Following the database construction procedure from LINDAHL dataset^10^ and the pairwise feature generation process of each sequence pair from the FOLDpro dataset^5^, we created our test dataset from SCOP version 1.75. Since LINDAHL dataset is based on SCOP version 1.37, we carefully selected 124 protein domains which are not included in version 1.37 to make sure the test dataset is completely independent to our training dataset. The sequences with mutual pair sequence identity of 40% or less are extracted from the SCOP 1.75 subsets. We extracted 124 protein domains construct our test dataset, with 614, 336, and 300 protein pairs sharing the same family, superfamily and fold category (see Table 3).

For each pair of proteins in the LINDAHL dataset, a number of pairwise similarity features were calculated and used to characterize the protein pair. These similarity features used were obtained through five types of sequence alignment and/or protein structure prediction tools (i.e., sequence-sequence alignment, sequence-family information, sequence-profile alignment, profile-profile alignment and structural information) and selected based on their use in prior works^5^. Full details for the feature generation script can be found in the FOLDpro study^5^. This resulted in a set of 84 features for each protein pair. For the SCOP_TEST dataset, pairwise features were generated by characterizing their sequence alignment, sequence or family information, profile-profile alignment and their structural information^5^ for all 15,252 protein pairs in this dataset. We considered the same strategy of feature generation as the FOLDpro dataset, which also resulted in a total of 84 features which were used to train our methods, DN-FoldS, DN-Fold and RFDN-Fold. The results of this head-to-head comparison are shown in Table 2.

To construct the regression dataset for our experiment (i.e., the dataset used to train and evaluate DN-FoldR), pdb structure files for each of 976 proteins in the LINDAHL dataset from SCOP 1.75 were downloaded from the Protein Data Bank^1^. After preprocessing the pdb files, the sequence identity from the pdb file and SCOP 1.37 was checked. Of the 976 proteins, 973 had a matching structure file. The exceptions were domains: 1alo-d1alo_5, 1alo-d1alo_6, 1alo-d1alo_7. The detailed relation between sequence id and pdb id can be accessed from the supplementary data. Finally, 973 proteins were used to generate a TM-score for a total of 945,756 protein pairs which were combined with the FOLDpro feature data as the final regression dataset. More specifically, during the calculation of the TM-score by the structure alignment tool TM-align^26^, the prior information of protein pair’s structures was used to determine the choice of TM-score. If the protein pair belongs to the same fold, the higher TM-score normalized by short sequence was be chosen. Otherwise, the lower TM-score normalized by long sequence was chosen as similarity score. We applied the same strategy on the evaluation dataset, which generated 15,252 pairwise TM-scores for 124 proteins. The distribution of similarity scores from two categories is illustrated in Fig. 6(a,b).

### DN-Fold: Deep Learning Approach for Protein Fold Recognition

Determining if two proteins pertain to the same structural fold is a problem that can be readily addressed as a binary classification problem. In this context, a protein pair which shares the same structural fold is labeled as ‘1’ (i.e., the positive class) and pairs from dissimilar folds are labeled as ‘–1’ (i.e., the negative class). Using the aforementioned features, a function can be learned to map the inputs from the feature space to the correct classification. With DN-Fold, the classification task was performed using deep networks.

Conceptually, a deep network (DN) is akin to a standard, two layer neural network but typically contains several more hidden layers and can differ in the way the model is initialized. The additional hidden layers allow the network to capture more correlations and patterns present in the data and this often leads to increases in performance. Another advantage of deep networks is that they can be pre-initialized using a layer-by-layer technique that involves stacking several restricted Boltmann machines (RBMs). The end result is an unsupervised learning process that first attempts to learn patterns in the data and then maps these learned patterns to the proper labels. Furthermore, using unsupervised learning to initialize weights between adjacent layers of transforming feature representations largely avoids the vanishing gradient problem which can occur when training a standard multi-layer neural network. This happens when the error gradients used to adjust weights quickly approach 0 as they are propagated through hidden layers. Thus, a deep learning network has the advantage of multiple-layer abstraction while largely avoiding the pitfall of training a multi-layer neural network, which leads to its excellent performance in several machine learning problems such as face recognition, hand writing recognition, and protein contact map prediction^7^. Full details on the theory related to RBMs and training deep networks is provided in the foundational work by Hinton and Salakhutdinov^27^. For details on applying deep networks, consult Hinton’s practical guide^28^ for general information and for use as it applies to protein structure prediction see applications developed for protein contact prediction^9^ and order/disorder prediction^8^.

**Figure 9:**
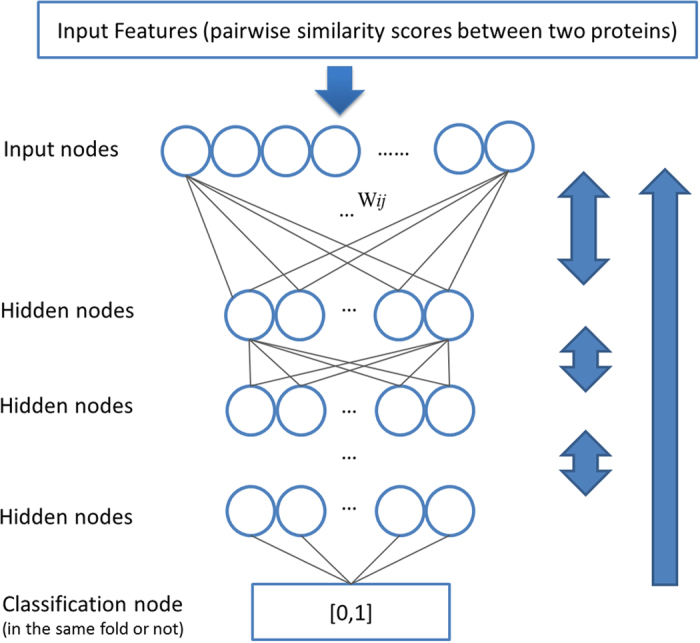
A deep learning network for classifying if two proteins share the same structural fold.

In this work, initialization of network was performed layer by layer utilizing a restricted Boltzmann machine (RBM). The RBM was used to initialize the weights in DNs by learning the weights between every two adjacent layers in a step-wise approach, starting from the visible layer, until reaching the last hidden layer. After the weights have been learned by the first RBM, it is applied to each training example and the probability for activating the hidden layers can be calculated and used as the visible layer for another RBM. This procedure of training a RBM can be repeated several times to create a multilayer network. A one layer standard feed-forward neural network is added at the top of the last hidden layer, taking activation probabilities of the last hidden layer as input to predict the label of the input values of the visible layer. Then all the nodes can be regarded as deterministic and the whole DN can be fine-tuned to adjust the weights using the standard back propagation algorithm to minimize the cross-entropy error 28,29,30,31. This process was completed over 25 epochs. The batchsize was set to 1000 training examples and the output of the DN was the prediction value of our method, DN-FoldS. The architecture of the overall deep network used in DN-FoldS was X-100-100-35-1 with X being the size of the input layer (i.e., the size of the feature vector). Figure 9 illustrates the model architecture and layer-by-layer learning procedure.

Different model architectures were trained using the same learning procedure but varying the number of hidden nodes and the epoch for each training process. Table 4 shows the 14 model architectures trained in this experiment. In order to balance the model bias and variance, an ensemble strategy was applied to average the outputs of these candidate deep networks. Specifically, each model was evaluated by the AUC value and the top ranked models were selected to construct the final ensemble network. In this work, we averaged the predictions of all 14 models, and the output of the ensemble of networks was the final prediction results of our method, DN-Fold.

The training and evaluation of DN-Fold was done using an in-house implementation of the algorithms needed to train RBMs and neural networks (i.e., standard back propagation). To increase the speed at which models could be trained and evaluated, the algorithms were implemented in terms of matrix operations and CUDAMat was used^32^. This allowed the more expensive operations to be performed on a CUDA-enabled GPU.

### Combination of Deep Learning Networks with Random Forests

Besides DN-Fold, an additional meta-method was developed and tested. This meta-method is called RFDN-Fold and combines DN-Fold and RF-Fold – another fold recognition method we developed based on random forests^11^. This meta approach is a simple sum of the outputs from DN-Fold and RF-Fold.

### DN-FoldR: Deep Learning Approach for Protein Fold Regression

Another means of approaching this problem is to learn a real-value measurement of structural similarity distributed over the structural space. A linear regression model could be used to map the input features for a protein pair to a structural similarity score (e.g., TM-Score). The underlying assumption is that protein pairs which share the same structural fold will have relatively larger structural similarity scores. To ease implementation and interpretation, the similarity score can be between 0 and 1.

The learning procedure was accomplished through two nonlinear hidden layers with rectified linear nodes and a linear layer was added as the last layer in the network for regression. The weights were randomly initialized within range [−0.5, 0.5]. The log likelihood of data was maximized by stochastic gradient decent with learning rate as 0.01, and the batch size was set to 1000 with maximum epoch as 50. The architecture of such deep network was X-100-100-35-1 with X being the size of the input layer. The machine learning package Pylean2^33^ was used to train the whole network on the 10 fold cross-validation sets.

## Training and Benchmarking. 

The LINDAHL dataset was partitioned into 10 subsets for the purpose of cross-validation. All the protein pairs that have the same query protein were put into the same subset. Then 9 of the 10 subsets were used for training and the remaining subset was used for testing. If a query protein in the training set was found in the test set as the template, it was also removed from the test set. Training and testing was repeated 10 times and the standard sensitivity (recall)/specificity (precision) was used to evaluate the classification results. Moreover, using the same evaluation procedure described elsewhere in the literature^5^,^10^,^23^,^24^,^25^, we compared DN-Fold with 17 other methods using the fold recognition success rate for the Top 1 and Top 5 ranked templates for the query proteins. The success rate (also called sensitivity) was calculated by taking the Top 1 or the Top 5 template proteins ranked for each query protein and calculating the percentage of query proteins having at least one correct template ranked Top 1, or within the Top 5^5^,^10^.

To further assess DN-Fold, predictions were made using the proteins contained in the SCOP_TEST set. After generating the features for all protein pairs, we retrained the DN-Fold method and RF-Fold, a method from a previous work, on the LINDAHL dataset using the same features and evaluated the model on our new dataset, SCOP_TEST, based on the success rate of Top 1 and Top 5 for the family, superfamily and fold levels.

DN-Fold is available as a web service at http://iris.rnet.missouri.edu/dnfold.

## Additional Information

**How to cite this article**: Jo, T. *et al.* Improving Protein Fold Recognition by Deep Learning Networks. *Sci. Rep.* **5**, 17573; doi: 10.1038/srep17573 (2015).

## Supplementary Material

Supplementary Information

## Figures and Tables

**Table 1:** The sensitivity (fold recognition success rate) of 22 fold recognition methods on the LINDAHL dataset at the three levels (family, superfamily, and fold) for the Top 1 prediction or Top 5 predictions for query proteins.

Method	Family		Superfamily		Fold	
	Top 1	Top 5	Top 1	Top 5	Top 1	Top 5
PSI-Blast	71.2	72.3	27.4	27.9	4	4.7
HMMER	67.7	73.5	20.7	31.3	4.4	14.6
SAM-T98	70.1	75.4	28.3	38.9	3.4	18.7
BLASTLINK	74.6	78.9	29.3	40.6	6.9	16.5
SSERCH	68.6	75.5	20.7	32.5	5.6	15.6
SSHMM	63.1	71.7	18.4	31.6	6.9	24
THREADER	49.2	58.9	10.8	24.7	14.6	37.7
Fugue	82.2	85.8	41.9	53.2	12.5	26.8
SPARKS	81.6	88.1	52.5	69.1	28.7	47.7
SP3	81.6	86.8	55.3	67.7	30.8	47.4
HHpred	82.9	87.1	58	70	25.2	39.4
SP4	80.9	86.3	57.8	57.8	30.8	53.6
SP5	82.4	87.6	59.8	70	37.9	58.7
RAPTOR	86.6	89.3	56.3	69	38.2	58.7
SPARKS-X	84.1	90.3	59.0	76.3	45.2	67.0
BoostThreader	86.5	90.5	66.1	76.4	42.6	57.4
RF-Fold	84.5	91.5	63.4	79.3	40.8	58.3
FOLDpro	85	89.9	55	70	26.5	48.3
DN-Fold	84.5	91.2	61.5	76.5	33.6	60.7
RFDN-Fold	84.7	91.5	65.7	78.8	37.7	61.7
DN-FoldS	84.1	91.2	62.7	76.7	33.3	57.9
DN-FoldR	82.3	88.3	56.0	71.0	27.4	56.7

Protein pairs (query – template proteins) that have substantial sequence similarity belong to the same family whose structural similarity is relatively easy to recognize. Protein pairs that have medium sequence similarity belong to the same superfamily whose structural similarity is generally difficult to recognize. Protein pairs that share structural fold, but have little or no sequence similarity belong to the same fold whose structural similarity is very hard to recognize.

**Table 2:** The sensitivity on the SCOP 1.75 evaluation set (SCOP_TEST) at the three levels for the Top 1 prediction or Top 5 predictions for query proteins.

Method	Family		Superfamily		Fold	
	Top 1	Top 5	Top 1	Top 5	Top 1	Top 5
RFDN-Fold	93.4	97.2	82.1	91.1	61.1	86.1
RF-Fold	93.4	98.1	83.9	91.1	55.6	72.2
DN-Fold	94.3	97.2	82.1	91.1	61.1	86.1
DN-FoldS	94.3	98.1	82.1	91.1	61.1	86.1
DN-FoldR	93.4	97.2	80.4	89.3	69.4	83.3

**Table 3:** Size of the SCOP 1.75 evaluation set (SCOP_TEST).

Evaluation Dataset		
	Protein sequence	Protein pair
Family	106	614
Superfamily	56	336
Fold	36	300
Total	124	1250

Here 106, 56 and 36 possible correct hits can be detected for family, superfamily and fold category. The true protein pair for all three levels are 614, 336, and 300, with a total of 1250 pairs among 15,252 pairwise sequences.

**Table 4:** 14 deep learning architectures with different hidden units and epoch.

INDEX	ARCHITECTURE
MODEL 1	84-100-100-30-1_60 epoch
MODEL 2	84-250-35-1_60 epoch
MODEL 3	84-100-100-25-1_30 epoch
MODEL 4	84-100-100-25-1_60 epoch
MODEL 5	84-150-150-25-1_60 epoch
MODEL 6	84-120-120-30-1_30 epoch
MODEL 7	84-250-35-1_30 epoch
MODEL 8	84-100-100-35-1_60 epoch
MODEL 9	84-100-35-1_30 epoch
MODEL 10	84-100-100-30-1_30 epoch
MODEL 11	84-100-100-35-1_30 epoch
MODEL 12	84-250-1_30_epoch
MODEL 13	84-150-150-25-1_30 epoch
MODEL 14	84-150_150_35_1_30 epoch

In this table 84-X-X-X-1_Y represent the architecture in the deep learning network, where X denotes the number of hidden nodes in each hidden layer, and Y denotes the epoch in each **training process.**
