# Supplementary material for: Improving Protein Fold Recognition by Deep Learning Networks
Source: Sci Rep. 2015 Dec 4;5:17573. doi: 10.1038/srep17573 (PMC4669437; doi:10.1038/srep17573)
Supplement: Supplementary Information [file srep17573-s1.pdf]

Improving Protein Fold Recognition by Deep Learning Networks

Taeho Jo[1,2], Jie Hou[1], Jesse Eickholt[3] and Jianlin Cheng[1*]

[1]Department of Computer Science, University of Missouri, Columbia, MO 65211, USA
[2]Department of Biological Chemistry, University of Michigan, Ann Arbor, MI, 48109, USA
[3] Department of Computer Science, Central Michigan University, Mount Pleasant, MI 48859, USA
*Corresponding author: chengji@missouri.edu

Prediction results on SCOP_TEST by DN-Fold

Family											
Name	SCOP ID	Length	Member	Rank1 Hit	Rank5 Hit	Rank1 List	Rank5 List				
d1b9ha_	c.67.1.4	385	2	1	1	d1o69a_(c.67.1.4)	d1o69a_(c.67.1.4)	d1m6sa_(c.67.1.1)	d1cl1a_(c.67.1.3)	d2ctza1(c.67.1.3)	d1m32a_(c.67.1.3)
d1brwa3	d.41.3.1	104	1	1	1	d2tpta3(d.41.3.1)	d2tpta3(d.41.3.1)	d2d0oa1(c.8.6.1)	d1wi4a1(b.36.1.1)	d1hdmb2(d.19.1.1)	d2cssa1(b.36.1.1)
d1byia_	c.37.1.10	225	2	1	1	d1nija1(c.37.1.10)	d1vcoa2(c.37.1.10)	d1nija1(c.37.1.10)	d1kkoa1(c.1.11.2)	d2d0oa1(c.8.6.1)	d1eoka_(c.1.8.5)
d1c7na_	c.67.1.3	395	4	0	1	d2r5ea1(c.67.1.1)	d1m32a_(c.67.1.3)	d1v72a1(c.67.1.1)	d1m6sa_(c.67.1.1)	d2r5ea1(c.67.1.1)	d2hoxa1(c.67.1.1)
d1cl1a_	c.67.1.3	392	4	1	1	d2ctza1(c.67.1.3)	d1m32a_(c.67.1.3)	d1iuga_(c.67.1.3)	d2ctza1(c.67.1.3)	d1b9ha_(c.67.1.4)	d1o69a_(c.67.1.4)
d1ctja_	a.3.1.1	90	6	1	1	d2c8sa1(a.3.1.1)	d2c8sa1(a.3.1.1)	d1gu2a_(a.3.1.1)	d1wvec1(a.3.1.1)	d1i8oa_(a.3.1.1)	d1dw0a_(a.3.1.1)
d1djqa1	c.1.4.1	341	1	1	1	d1ps9a1(c.1.4.1)	d1ps9a1(c.1.4.1)	d1wbha1(c.1.10.1)	d1rd5a_(c.1.2.4)	d1kkoa1(c.1.11.2)	d1vc4a_(c.1.2.4)
d1dw0a_	a.3.1.1	113	6	1	1	d1gu2a_(a.3.1.1)	d1gu2a_(a.3.1.1)	d2c8sa1(a.3.1.1)	d1e29a_(a.3.1.1)	d1i8oa_(a.3.1.1)	d1ctja_(a.3.1.1)
d1e29a_	a.3.1.1	136	6	1	1	d1ctja_(a.3.1.1)	d1wvec1(a.3.1.1)	d2c8sa1(a.3.1.1)	d1i8oa_(a.3.1.1)	d1ctja_(a.3.1.1)	d1gu2a_(a.3.1.1)
d1ejda_	d.68.2.2	420	2	1	1	d1g6sa_(d.68.2.2)	d1g6sa_(d.68.2.2)	d1rf6a_(d.68.2.2)	d1m6sa_(c.67.1.1)	d1iuga_(c.67.1.3)	d1u08a_(c.67.1.1)
d1eq3a_	d.26.1.1	97	6	1	1	d1m5ya3(d.26.1.1)	d1t11a3(d.26.1.1)	d1jvwa_(d.26.1.1)	d1m5ya3(d.26.1.1)	d1hdma2(d.19.1.1)	d1nj8a2(d.68.5.1)
d1ewsa_	g.9.1.1	33	3	1	1	d1ijva_(g.9.1.1)	d1ijva_(g.9.1.1)	d1kj6a_(g.9.1.1)	d1zuea1(g.9.1.1)	d1hdma2(d.19.1.1)	d1brwa3(d.41.3.1)
d1g3ka_	d.153.1.4	174	7	1	1	d1ryph_(d.153.1.4)	d1ryph_(d.153.1.4)	d1rypg_(d.153.1.4)	d1rype_(d.153.1.4)	d1rypb_(d.153.1.4)	d1ryp2_(d.153.1.4)
d1g6sa_	d.68.2.2	428	2	1	1	d1ejda_(d.68.2.2)	d1ejda_(d.68.2.2)	d1rf6a_(d.68.2.2)	d1m32a_(c.67.1.3)	d2ctza1(c.67.1.3)	d1m6sa_(c.67.1.1)
d1g9oa_	b.36.1.1	92	10	1	1	d1rzxa_(b.36.1.1)	d1rzxa_(b.36.1.1)	d1q3oa_(b.36.1.1)	d1uewa_(b.36.1.1)	d1r6ja_(b.36.1.1)	d1wi4a1(b.36.1.1)
d1gu2a_	a.3.1.1	125	6	1	1	d1dw0a_(a.3.1.1)	d1i8oa_(a.3.1.1)	d1e29a_(a.3.1.1)	d2c8sa1(a.3.1.1)	d1ctja_(a.3.1.1)	d1dw0a_(a.3.1.1)
d1hc7a3	d.68.5.1	75	1	1	1	d1nj8a2(d.68.5.1)	d1nj8a2(d.68.5.1)	d1kj6a_(g.9.1.1)	d1r6ja_(b.36.1.1)	d1hdmb2(d.19.1.1)	d2bkya1(d.68.6.1)
d1hdma2	d.19.1.1	82	6	1	1	d1hdmb2(d.19.1.1)	d1hyrc2(d.19.1.1)	d1lqva_(d.19.1.1)	d2h26a2(d.19.1.1)	d1hdmb2(d.19.1.1)	d1uvqb2(d.19.1.1)
d1hdmb2	d.19.1.1	86	6	1	1	d1uvqb2(d.19.1.1)	d1hdma2(d.19.1.1)	d1uvqb2(d.19.1.1)	d2h26a2(d.19.1.1)	d1lqva_(d.19.1.1)	d2k0bx1(a.5.2.1)
d1hl2a_	c.1.10.1	296	2	0	1	d1rd5a_(c.1.2.4)	d1wbha1(c.1.10.1)	d1to3a_(c.1.10.1)	d1rd5a_(c.1.2.4)	d1ps9a1(c.1.4.1)	d1i4na_(c.1.2.4)
d1hxva_	d.26.1.1	86	6	1	1	d1jvwa_(d.26.1.1)	d1jvwa_(d.26.1.1)	d1u79a_(d.26.1.1)	d1t11a3(d.26.1.1)	d1q6ha_(d.26.1.1)	d1q3oa_(b.36.1.1)
d1hyrc2	d.19.1.1	182	6	1	1	d1t7va2(d.19.1.1)	d1uvqb2(d.19.1.1)	d1t7va2(d.19.1.1)	d1hdma2(d.19.1.1)	d1lqva_(d.19.1.1)	d2h26a2(d.19.1.1)
d1i4na_	c.1.2.4	252	2	1	1	d1vc4a_(c.1.2.4)	d1rd5a_(c.1.2.4)	d1vc4a_(c.1.2.4)	d1to3a_(c.1.10.1)	d1wbha1(c.1.10.1)	d1hl2a_(c.1.10.1)
d1i8oa_	a.3.1.1	115	6	1	1	d1e29a_(a.3.1.1)	d1wvec1(a.3.1.1)	d1e29a_(a.3.1.1)	d1dw0a_(a.3.1.1)	d2c8sa1(a.3.1.1)	d1ctja_(a.3.1.1)
d1ijva_	g.9.1.1	37	3	1	1	d1kj6a_(g.9.1.1)	d1kj6a_(g.9.1.1)	d1zuea1(g.9.1.1)	d1ewsa_(g.9.1.1)	d1jdqa_(d.68.3.3)	d1hc7a3(d.68.5.1)
d1iuga_	c.67.1.3	349	4	1	1	d1m32a_(c.67.1.3)	d1cl1a_(c.67.1.3)	d1m32a_(c.67.1.3)	d1v72a1(c.67.1.1)	d1m6sa_(c.67.1.1)	d1o69a_(c.67.1.4)
d1jdqa_	d.68.3.3	99	1	1	1	d1pava_(d.68.3.3)	d1pava_(d.68.3.3)	d1msza_(d.68.7.1)	d1eq3a_(d.26.1.1)	d1uewa_(b.36.1.1)	d1m5ya3(d.26.1.1)
d1jj7a_	c.37.1.12	252	3	1	1	d3dhwc1(c.37.1.12)	d3dhwc1(c.37.1.12)	d2onka1(c.37.1.12)	d1l2ta_(c.37.1.12)	d1ps9a1(c.1.4.1)	d1djqa1(c.1.4.1)
d1jvwa_	d.26.1.1	161	6	1	1	d1u79a_(d.26.1.1)	d1u79a_(d.26.1.1)	d1t11a3(d.26.1.1)	d1q6ha_(d.26.1.1)	d1eq3a_(d.26.1.1)	d1hxva_(d.26.1.1)
d1kj6a_	g.9.1.1	46	3	1	1	d1ijva_(g.9.1.1)	d1zuea1(g.9.1.1)	d1ewsa_(g.9.1.1)	d1ijva_(g.9.1.1)	d1hc7a3(d.68.5.1)	d1jdqa_(d.68.3.3)
d1l2ta_	c.37.1.12	231	3	1	1	d3dhwc1(c.37.1.12)	d3dhwc1(c.37.1.12)	d1jj7a_(c.37.1.12)	d2onka1(c.37.1.12)	d1g6oa_(c.37.1.11)	d1nija1(c.37.1.10)
d1l5ja2	c.8.2.1	213	1	1	1	d2b3ya1(c.8.2.1)	d2b3ya1(c.8.2.1)	d1g6oa_(c.37.1.11)	d2hi6a1(c.8.2.3)	d1nxja_(c.8.7.1)	d1kbla2(c.8.1.1)
d1lqva_	d.19.1.1	173	6	1	1	d2h26a2(d.19.1.1)	d2h26a2(d.19.1.1)	d1t7va2(d.19.1.1)	d1uvqb2(d.19.1.1)	d1hyrc2(d.19.1.1)	d1hdmb2(d.19.1.1)
d1m32a_	c.67.1.3	362	4	1	1	d1iuga_(c.67.1.3)	d1iuga_(c.67.1.3)	d1m6sa_(c.67.1.1)	d1b9ha_(c.67.1.4)	d1o69a_(c.67.1.4)	d1v72a1(c.67.1.1)
d1m5ya3	d.26.1.1	109	6	1	1	d1eq3a_(d.26.1.1)	d1hxva_(d.26.1.1)	d1eq3a_(d.26.1.1)	d1hdma2(d.19.1.1)	d1jdqa_(d.68.3.3)	d1nj8a2(d.68.5.1)
d1m6sa_	c.67.1.1	344	4	1	1	d1v72a1(c.67.1.1)	d1v72a1(c.67.1.1)	d1b9ha_(c.67.1.4)	d1o69a_(c.67.1.4)	d1m32a_(c.67.1.3)	d1ohwa_(c.67.1.4)
d1mn3a_	a.5.2.4	55	1	1	1	d1otra_(a.5.2.4)	d1otra_(a.5.2.4)	d2bwba1(a.5.2.1)	d3e46a1(a.5.2.1)	d1wgna_(a.5.2.1)	d2dnaa1(a.5.2.1)
d1msza_	d.68.7.1	63	1	1	1	d1whra_(d.68.7.1)	d1whra_(d.68.7.1)	d1nj8a2(d.68.5.1)	d1ttea1(a.5.2.1)	d1pava_(d.68.3.3)	d1jdqa_(d.68.3.3)
d1nija1	c.37.1.10	223	2	1	1	d1byia_(c.37.1.10)	d1byia_(c.37.1.10)	d2onka1(c.37.1.12)	d1g6oa_(c.37.1.11)	d3dhwc1(c.37.1.12)	d1l2ta_(c.37.1.12)
d1nj8a2	d.68.5.1	63	1	1	1	d1hc7a3(d.68.5.1)	d1hc7a3(d.68.5.1)	d1msza_(d.68.7.1)	d1eq3a_(d.26.1.1)	d2f23a2(d.26.1.2)	d1m5ya3(d.26.1.1)
d1o69a_	c.67.1.4	375	2	1	1	d1b9ha_(c.67.1.4)	d1b9ha_(c.67.1.4)	d1m32a_(c.67.1.3)	d1cl1a_(c.67.1.3)	d2ctza1(c.67.1.3)	d1m6sa_(c.67.1.1)
d1oaia_	a.5.2.3	60	1	1	1	d1v92a_(a.5.2.3)	d1v92a_(a.5.2.3)	d1xb2b1(a.5.2.2)	d1vdla_(a.5.2.1)	d1wj7a1(a.5.2.1)	d1wgna_(a.5.2.1)
d1ohwa_	c.67.1.4	462	2	0	0	d1m6sa_(c.67.1.1)	d1m6sa_(c.67.1.1)	d1m32a_(c.67.1.3)	d1iuga_(c.67.1.3)	d1u08a_(c.67.1.1)	d1v72a1(c.67.1.1)
d1otra_	a.5.2.4	50	1	0	0	d1wj7a1(a.5.2.1)	d1wj7a1(a.5.2.1)	d2crna1(a.5.2.1)	d1wgna_(a.5.2.1)	d1wiva_(a.5.2.1)	d1veka_(a.5.2.1)
d1pava_	d.68.3.3	79	1	1	1	d1jdqa_(d.68.3.3)	d1jdqa_(d.68.3.3)	d1eq3a_(d.26.1.1)	d1msza_(d.68.7.1)	d1r6ja_(b.36.1.1)	d1hdma2(d.19.1.1)
d1pgya_	a.5.2.1	48	14	1	1	d1wiva_(a.5.2.1)	d2k0bx1(a.5.2.1)	d1wiva_(a.5.2.1)	d1veka_(a.5.2.1)	d1wgna_(a.5.2.1)	d3e46a1(a.5.2.1)
d1ps9a1	c.1.4.1	331	1	1	1	d1djqa1(c.1.4.1)	d1djqa1(c.1.4.1)	d1vc4a_(c.1.2.4)	d1to3a_(c.1.10.1)	d1hl2a_(c.1.10.1)	d1rd5a_(c.1.2.4)
d1q3oa_	b.36.1.1	105	10	1	1	d1rzxa_(b.36.1.1)	d1rzxa_(b.36.1.1)	d1r6ja_(b.36.1.1)	d1vaea_(b.36.1.1)	d2cssa1(b.36.1.1)	d1wi4a1(b.36.1.1)
d1q6ha_	d.26.1.1	211	6	1	1	d1jvwa_(d.26.1.1)	d1t11a3(d.26.1.1)	d1jvwa_(d.26.1.1)	d1u79a_(d.26.1.1)	d1hxva_(d.26.1.1)	d1rypb_(d.153.1.4)
d1r61a_	c.8.8.1	206	1	1	1	d2b0aa1(c.8.8.1)	d2b0aa1(c.8.8.1)	d1a9xb1(c.8.3.1)	d2b3ya1(c.8.2.1)	d1q3oa_(b.36.1.1)	d1nxja_(c.8.7.1)
d1r6ja_	b.36.1.1	83	10	1	1	d1tp5a1(b.36.1.1)	d1tp5a1(b.36.1.1)	d1rzxa_(b.36.1.1)	d1rgwa_(b.36.1.1)	d1uewa_(b.36.1.1)	d2cssa1(b.36.1.1)
d1rd5a_	c.1.2.4	262	2	1	1	d1i4na_(c.1.2.4)	d1i4na_(c.1.2.4)	d1vc4a_(c.1.2.4)	d1to3a_(c.1.10.1)	d1hl2a_(c.1.10.1)	d1wbha1(c.1.10.1)
d1rf6a_	d.68.2.2	428	2	1	1	d1ejda_(d.68.2.2)	d1ejda_(d.68.2.2)	d1g6sa_(d.68.2.2)	d1u08a_(c.67.1.1)	d1m32a_(c.67.1.3)	d1iuga_(c.67.1.3)
d1rgwa_	b.36.1.1	86	10	1	1	d1tp5a1(b.36.1.1)	d1tp5a1(b.36.1.1)	d1r6ja_(b.36.1.1)	d1va8a1(b.36.1.1)	d1rzxa_(b.36.1.1)	d1wi4a1(b.36.1.1)
d1ryp2_	d.153.1.4	234	7	1	1	d1rypc_(d.153.1.4)	d1rypc_(d.153.1.4)	d1ryph_(d.153.1.4)	d1rypg_(d.153.1.4)	d1g3ka_(d.153.1.4)	d1rypb_(d.153.1.4)
d1rypb_	d.153.1.4	251	7	1	1	d1ryp2_(d.153.1.4)	d1ryp2_(d.153.1.4)	d1rypc_(d.153.1.4)	d1rype_(d.153.1.4)	d1rypg_(d.153.1.4)	d1g3ka_(d.153.1.4)
d1rypc_	d.153.1.4	245	7	1	1	d1yara1(d.153.1.4)	d1yara1(d.153.1.4)	d1ryp2_(d.153.1.4)	d1rypg_(d.153.1.4)	d1rypb_(d.153.1.4)	d1ryph_(d.153.1.4)
d1rype_	d.153.1.4	243	7	1	1	d1rypg_(d.153.1.4)	d1rypg_(d.153.1.4)	d1g3ka_(d.153.1.4)	d1ryp2_(d.153.1.4)	d1yara1(d.153.1.4)	d1ryph_(d.153.1.4)
d1rypg_	d.153.1.4	245	7	1	1	d1yara1(d.153.1.4)	d1yara1(d.153.1.4)	d1rype_(d.153.1.4)	d1rypc_(d.153.1.4)	d1ryp2_(d.153.1.4)	d1g3ka_(d.153.1.4)
d1ryph_	d.153.1.4	206	7	1	1	d1ryp2_(d.153.1.4)	d1ryp2_(d.153.1.4)	d1yara1(d.153.1.4)	d1rype_(d.153.1.4)	d1g3ka_(d.153.1.4)	d1rypc_(d.153.1.4)
d1rzxa_	b.36.1.1	99	10	1	1	d1q3oa_(b.36.1.1)	d1q3oa_(b.36.1.1)	d1tp5a1(b.36.1.1)	d1g9oa_(b.36.1.1)	d1wi4a1(b.36.1.1)	d1vaea_(b.36.1.1)
d1t11a3	d.26.1.1	114	6	1	1	d1hxva_(d.26.1.1)	d1q6ha_(d.26.1.1)	d1hxva_(d.26.1.1)	d1u79a_(d.26.1.1)	d1jvwa_(d.26.1.1)	d2f23a2(d.26.1.2)
d1t7va2	d.19.1.1	180	6	1	1	d2h26a2(d.19.1.1)	d2h26a2(d.19.1.1)	d1uvqb2(d.19.1.1)	d1hdma2(d.19.1.1)	d1hyrc2(d.19.1.1)	d1lqva_(d.19.1.1)
d1to3a_	c.1.10.1	292	2	0	1	d1rd5a_(c.1.2.4)	d1wbha1(c.1.10.1)	d1rd5a_(c.1.2.4)	d1vc4a_(c.1.2.4)	d1ps9a1(c.1.4.1)	d1i4na_(c.1.2.4)
d1tp5a1	b.36.1.1	103	10	1	1	d1q3oa_(b.36.1.1)	d1q3oa_(b.36.1.1)	d1rgwa_(b.36.1.1)	d1va8a1(b.36.1.1)	d1g9oa_(b.36.1.1)	d1r6ja_(b.36.1.1)
d1ttea1	a.5.2.1	56	14	1	1	d1veka_(a.5.2.1)	d1veka_(a.5.2.1)	d1wgna_(a.5.2.1)	d1wiva_(a.5.2.1)	d2cpwa1(a.5.2.1)	d2crna1(a.5.2.1)
d1u08a_	c.67.1.1	383	4	1	1	d2r5ea1(c.67.1.1)	d2r5ea1(c.67.1.1)	d1m6sa_(c.67.1.1)	d1v72a1(c.67.1.1)	d2hoxa1(c.67.1.1)	d1m32a_(c.67.1.3)
d1u79a_	d.26.1.1	126	6	1	1	d1jvwa_(d.26.1.1)	d1jvwa_(d.26.1.1)	d1q6ha_(d.26.1.1)	d1hxva_(d.26.1.1)	d1t11a3(d.26.1.1)	d1uewa_(b.36.1.1)
d1uewa_	b.36.1.1	115	10	1	1	d1g9oa_(b.36.1.1)	d1g9oa_(b.36.1.1)	d1rgwa_(b.36.1.1)	d2cssa1(b.36.1.1)	d1vaea_(b.36.1.1)	d1wi4a1(b.36.1.1)
d1uvqb2	d.19.1.1	93	6	1	1	d1hdmb2(d.19.1.1)	d1t7va2(d.19.1.1)	d1hdmb2(d.19.1.1)	d1lqva_(d.19.1.1)	d1hyrc2(d.19.1.1)	d1hdma2(d.19.1.1)
d1v72a1	c.67.1.1	346	4	1	1	d1m6sa_(c.67.1.1)	d1m6sa_(c.67.1.1)	d1u08a_(c.67.1.1)	d1m32a_(c.67.1.3)	d1c7na_(c.67.1.3)	d1iuga_(c.67.1.3)
d1v92a_	a.5.2.3	47	1	1	1	d1oaia_(a.5.2.3)	d1oaia_(a.5.2.3)	d1wgna_(a.5.2.1)	d2bwba1(a.5.2.1)	d1wiva_(a.5.2.1)	d1xb2b1(a.5.2.2)
d1va8a1	b.36.1.1	101	10	1	1	d2cssa1(b.36.1.1)	d2cssa1(b.36.1.1)	d1r6ja_(b.36.1.1)	d1rgwa_(b.36.1.1)	d1wi4a1(b.36.1.1)	d1vaea_(b.36.1.1)
d1vaea_	b.36.1.1	112	10	1	1	d1uewa_(b.36.1.1)	d1r6ja_(b.36.1.1)	d1uewa_(b.36.1.1)	d1tp5a1(b.36.1.1)	d1rgwa_(b.36.1.1)	d1g9oa_(b.36.1.1)
d1vc4a_	c.1.2.4	255	2	1	1	d1i4na_(c.1.2.4)	d1i4na_(c.1.2.4)	d1rd5a_(c.1.2.4)	d1to3a_(c.1.10.1)	d1ps9a1(c.1.4.1)	d1wbha1(c.1.10.1)
d1vcoa2	c.37.1.10	273	2	1	1	d1byia_(c.37.1.10)	d1nija1(c.37.1.10)	d1byia_(c.37.1.10)	d1v72a1(c.67.1.1)	d3dhwc1(c.37.1.12)	d1ps9a1(c.1.4.1)
d1vdla_	a.5.2.1	81	14	1	1	d1wj7a1(a.5.2.1)	d1wj7a1(a.5.2.1)	d1wiva_(a.5.2.1)	d1vega_(a.5.2.1)	d2bwba1(a.5.2.1)	d2dnaa1(a.5.2.1)
d1vega_	a.5.2.1	84	14	1	1	d1veka_(a.5.2.1)	d1wiva_(a.5.2.1)	d1veka_(a.5.2.1)	d1wgna_(a.5.2.1)	d2crna1(a.5.2.1)	d1vdla_(a.5.2.1)
d1veka_	a.5.2.1	85	14	1	1	d2crna1(a.5.2.1)	d1wiva_(a.5.2.1)	d1vega_(a.5.2.1)	d2cpwa1(a.5.2.1)	d2crna1(a.5.2.1)	d1wgna_(a.5.2.1)
d1wbha1	c.1.10.1	214	2	0	0	d1vc4a_(c.1.2.4)	d1kkoa1(c.1.11.2)	d1rd5a_(c.1.2.4)	d1vc4a_(c.1.2.4)	d1djqa1(c.1.4.1)	d1i4na_(c.1.2.4)
d1wgna_	a.5.2.1	64	14	1	1	d1wiva_(a.5.2.1)	d2bwba1(a.5.2.1)	d3e46a1(a.5.2.1)	d2cpwa1(a.5.2.1)	d1wiva_(a.5.2.1)	d2crna1(a.5.2.1)
d1whra_	d.68.7.1	125	1	1	1	d1msza_(d.68.7.1)	d1msza_(d.68.7.1)	d1e29a_(a.3.1.1)	d1kida_(c.8.5.1)	d1jo0a_(d.68.4.1)	d1i8oa_(a.3.1.1)
d1wi4a1	b.36.1.1	97	10	1	1	d1tp5a1(b.36.1.1)	d1g9oa_(b.36.1.1)	d1rgwa_(b.36.1.1)	d1tp5a1(b.36.1.1)	d1vaea_(b.36.1.1)	d1q3oa_(b.36.1.1)
d1wiva_	a.5.2.1	74	14	1	1	d1veka_(a.5.2.1)	d1wgna_(a.5.2.1)	d1veka_(a.5.2.1)	d2crna1(a.5.2.1)	d3e46a1(a.5.2.1)	d2cpwa1(a.5.2.1)
d1wj7a1	a.5.2.1	92	14	1	1	d1wiva_(a.5.2.1)	d1vdla_(a.5.2.1)	d1wiva_(a.5.2.1)	d1veka_(a.5.2.1)	d2bwba1(a.5.2.1)	d1wgna_(a.5.2.1)
d1wvec1	a.3.1.1	75	6	1	1	d1ctja_(a.3.1.1)	d1dw0a_(a.3.1.1)	d1e29a_(a.3.1.1)	d1i8oa_(a.3.1.1)	d1ctja_(a.3.1.1)	d2c8sa1(a.3.1.1)
d1yara1	d.153.1.4	222	7	1	1	d1rype_(d.153.1.4)	d1g3ka_(d.153.1.4)	d1rype_(d.153.1.4)	d1ryph_(d.153.1.4)	d1rypg_(d.153.1.4)	d1rypb_(d.153.1.4)
d1zuea1	g.9.1.1	43	3	1	1	d1kj6a_(g.9.1.1)	d1ewsa_(g.9.1.1)	d1ijva_(g.9.1.1)	d1kj6a_(g.9.1.1)	d1hc7a3(d.68.5.1)	d1hdmb2(d.19.1.1)
d2b0aa1	c.8.8.1	187	1	1	1	d1r61a_(c.8.8.1)	d1r61a_(c.8.8.1)	d1a9xb1(c.8.3.1)	d2d0oa1(c.8.6.1)	d1u08a_(c.67.1.1)	d1kbla2(c.8.1.1)
d2b3ya1	c.8.2.1	260	1	1	1	d1l5ja2(c.8.2.1)	d1l5ja2(c.8.2.1)	d1eoka_(c.1.8.5)	d1kbla2(c.8.1.1)	d1vc4a_(c.1.2.4)	d1i4na_(c.1.2.4)
d2bwba1	a.5.2.1	45	14	1	1	d2dnaa1(a.5.2.1)	d1wgna_(a.5.2.1)	d1veka_(a.5.2.1)	d1wiva_(a.5.2.1)	d2cpwa1(a.5.2.1)	d2dnaa1(a.5.2.1)
d2c8sa1	a.3.1.1	150	6	1	1	d1ctja_(a.3.1.1)	d1dw0a_(a.3.1.1)	d1ctja_(a.3.1.1)	d1e29a_(a.3.1.1)	d1i8oa_(a.3.1.1)	d1wvec1(a.3.1.1)
d2cpwa1	a.5.2.1	52	14	1	1	d2crna1(a.5.2.1)	d2crna1(a.5.2.1)	d1wgna_(a.5.2.1)	d1veka_(a.5.2.1)	d2dnaa1(a.5.2.1)	d1wiva_(a.5.2.1)
d2crna1	a.5.2.1	52	14	1	1	d1veka_(a.5.2.1)	d1veka_(a.5.2.1)	d2bwba1(a.5.2.1)	d1wiva_(a.5.2.1)	d1wgna_(a.5.2.1)	d2dnaa1(a.5.2.1)
d2cssa1	b.36.1.1	109	10	1	1	d1rgwa_(b.36.1.1)	d1rgwa_(b.36.1.1)	d1tp5a1(b.36.1.1)	d1g9oa_(b.36.1.1)	d1wi4a1(b.36.1.1)	d1va8a1(b.36.1.1)
d2ctza1	c.67.1.3	422	4	1	1	d1cl1a_(c.67.1.3)	d1iuga_(c.67.1.3)	d1m32a_(c.67.1.3)	d1cl1a_(c.67.1.3)	d1b9ha_(c.67.1.4)	d1m6sa_(c.67.1.1)
d2dkla1	a.5.2.1	73	14	1	1	d1wiva_(a.5.2.1)	d1wiva_(a.5.2.1)	d1vdla_(a.5.2.1)	d1veka_(a.5.2.1)	d2crna1(a.5.2.1)	d1wgna_(a.5.2.1)
d2dnaa1	a.5.2.1	51	14	1	1	d2bwba1(a.5.2.1)	d1wiva_(a.5.2.1)	d1veka_(a.5.2.1)	d1wgna_(a.5.2.1)	d2cpwa1(a.5.2.1)	d2bwba1(a.5.2.1)
d2h26a2	d.19.1.1	178	6	1	1	d1lqva_(d.19.1.1)	d1lqva_(d.19.1.1)	d1hyrc2(d.19.1.1)	d1t7va2(d.19.1.1)	d1hdma2(d.19.1.1)	d1uvqb2(d.19.1.1)
d2hoxa1	c.67.1.1	426	4	1	1	d1u08a_(c.67.1.1)	d1v72a1(c.67.1.1)	d1u08a_(c.67.1.1)	d2r5ea1(c.67.1.1)	d1c7na_(c.67.1.3)	d1m32a_(c.67.1.3)
d2k0bx1	a.5.2.1	53	14	1	1	d2dnaa1(a.5.2.1)	d1wiva_(a.5.2.1)	d2dnaa1(a.5.2.1)	d1veka_(a.5.2.1)	d1wgna_(a.5.2.1)	d2bwba1(a.5.2.1)
d2onka1	c.37.1.12	241	3	1	1	d1l2ta_(c.37.1.12)	d1l2ta_(c.37.1.12)	d1jj7a_(c.37.1.12)	d3dhwc1(c.37.1.12)	d1g6oa_(c.37.1.11)	d1nija1(c.37.1.10)
d2r5ea1	c.67.1.1	419	4	1	1	d1u08a_(c.67.1.1)	d1m6sa_(c.67.1.1)	d1v72a1(c.67.1.1)	d1u08a_(c.67.1.1)	d2hoxa1(c.67.1.1)	d1c7na_(c.67.1.3)
d2tpta3	d.41.3.1	106	1	1	1	d1brwa3(d.41.3.1)	d1brwa3(d.41.3.1)	d1tp5a1(b.36.1.1)	d2gyck1(d.41.4.2)	d1nj8a2(d.68.5.1)	d1wi4a1(b.36.1.1)
d3dhwc1	c.37.1.12	241	3	1	1	d1jj7a_(c.37.1.12)	d1jj7a_(c.37.1.12)	d2onka1(c.37.1.12)	d1l2ta_(c.37.1.12)	d1g6oa_(c.37.1.11)	d1nija1(c.37.1.10)
d3e46a1	a.5.2.1	43	14	1	1	d1wiva_(a.5.2.1)	d1wgna_(a.5.2.1)	d2dnaa1(a.5.2.1)	d2bwba1(a.5.2.1)	d2cpwa1(a.5.2.1)	d1wiva_(a.5.2.1)
Total Hit				100	103						
Total seq				106	106						
Success Rate				94.34%	97.17%						

Superfamily											
Name	SCOP ID	Length	Member	Rank1 Hit	Rank5 Hit	Rank1 List	Rank5 List				
d1a0tp_	f.4.3.2	414	1	1	1	d1t16a_(f.4.3.4)	d1t16a_(f.4.3.4)	d1rf6a_(d.68.2.2)	d2b0aa1(c.8.8.1)	d2isba1(c.8.9.1)	d1r61a_(c.8.8.1)
d1b9ha_	c.67.1.4	385	10	1	1	d2ctza1(c.67.1.3)	d1u08a_(c.67.1.1)	d1m6sa_(c.67.1.1)	d1cl1a_(c.67.1.3)	d2ctza1(c.67.1.3)	d1m32a_(c.67.1.3)
d1byia_	c.37.1.10	225	5	0	1	d1kkoa1(c.1.11.2)	d1g6oa_(c.37.1.11)	d1jj7a_(c.37.1.12)	d1kkoa1(c.1.11.2)	d2d0oa1(c.8.6.1)	d1eoka_(c.1.8.5)
d1c7na_	c.67.1.3	395	8	1	1	d2r5ea1(c.67.1.1)	d1b9ha_(c.67.1.4)	d1v72a1(c.67.1.1)	d1m6sa_(c.67.1.1)	d2r5ea1(c.67.1.1)	d2hoxa1(c.67.1.1)
d1cl1a_	c.67.1.3	392	8	1	1	d1b9ha_(c.67.1.4)	d1u08a_(c.67.1.1)	d2r5ea1(c.67.1.1)	d1m6sa_(c.67.1.1)	d1b9ha_(c.67.1.4)	d1o69a_(c.67.1.4)
d1eq3a_	d.26.1.1	97	1	0	0	d1nj8a2(d.68.5.1)	d1hdmb2(d.19.1.1)	d1jdqa_(d.68.3.3)	d1pava_(d.68.3.3)	d1hdma2(d.19.1.1)	d1nj8a2(d.68.5.1)
d1g6oa_	c.37.1.11	324	7	1	1	d1jj7a_(c.37.1.12)	d2onka1(c.37.1.12)	d1l2ta_(c.37.1.12)	d1jj7a_(c.37.1.12)	d3dhwc1(c.37.1.12)	d1nija1(c.37.1.10)
d1hxva_	d.26.1.1	86	1	1	1	d2f23a2(d.26.1.2)	d2f23a2(d.26.1.2)	d1r6ja_(b.36.1.1)	d1q3oa_(b.36.1.1)	d1g9oa_(b.36.1.1)	d1rzxa_(b.36.1.1)
d1iuga_	c.67.1.3	349	8	1	1	d1v72a1(c.67.1.1)	d2r5ea1(c.67.1.1)	d1v72a1(c.67.1.1)	d1m6sa_(c.67.1.1)	d1o69a_(c.67.1.4)	d1b9ha_(c.67.1.4)
d1jj7a_	c.37.1.12	252	4	1	1	d1g6oa_(c.37.1.11)	d1nija1(c.37.1.10)	d1g6oa_(c.37.1.11)	d1byia_(c.37.1.10)	d1ps9a1(c.1.4.1)	d1djqa1(c.1.4.1)
d1jvwa_	d.26.1.1	161	1	0	0	d1va8a1(b.36.1.1)	d1jdqa_(d.68.3.3)	d1va8a1(b.36.1.1)	d1uewa_(b.36.1.1)	d1q3oa_(b.36.1.1)	d1l5ja2(c.8.2.1)
d1kida_	c.8.5.1	194	1	1	1	d1q3qa2(c.8.5.2)	d1q3qa2(c.8.5.2)	d1nija1(c.37.1.10)	d2d0oa1(c.8.6.1)	d1i4na_(c.1.2.4)	d1wbha1(c.1.10.1)
d1l2ta_	c.37.1.12	231	4	1	1	d1g6oa_(c.37.1.11)	d1g6oa_(c.37.1.11)	d1nija1(c.37.1.10)	d1vcoa2(c.37.1.10)	d1djqa1(c.1.4.1)	d1kida_(c.8.5.1)
d1l5ja2	c.8.2.1	213	1	1	1	d2hi6a1(c.8.2.3)	d2hi6a1(c.8.2.3)	d1g6oa_(c.37.1.11)	d1nxja_(c.8.7.1)	d2d0oa1(c.8.6.1)	d1kbla2(c.8.1.1)
d1m32a_	c.67.1.3	362	8	1	1	d1v72a1(c.67.1.1)	d1m6sa_(c.67.1.1)	d1b9ha_(c.67.1.4)	d1o69a_(c.67.1.4)	d1v72a1(c.67.1.1)	d1u08a_(c.67.1.1)
d1m5ya3	d.26.1.1	109	1	0	0	d1hdma2(d.19.1.1)	d1hdmb2(d.19.1.1)	d1hdma2(d.19.1.1)	d1uvqb2(d.19.1.1)	d1jdqa_(d.68.3.3)	d1nj8a2(d.68.5.1)
d1m6sa_	c.67.1.1	344	8	1	1	d1ohwa_(c.67.1.4)	d1b9ha_(c.67.1.4)	d1o69a_(c.67.1.4)	d1m32a_(c.67.1.3)	d1ohwa_(c.67.1.4)	d1iuga_(c.67.1.3)
d1mn3a_	a.5.2.4	55	18	1	1	d1wgna_(a.5.2.1)	d2bwba1(a.5.2.1)	d3e46a1(a.5.2.1)	d2k0bx1(a.5.2.1)	d1wgna_(a.5.2.1)	d2dnaa1(a.5.2.1)
d1nija1	c.37.1.10	223	5	1	1	d2onka1(c.37.1.12)	d2onka1(c.37.1.12)	d1g6oa_(c.37.1.11)	d3dhwc1(c.37.1.12)	d1l2ta_(c.37.1.12)	d2d0oa1(c.8.6.1)
d1o69a_	c.67.1.4	375	10	1	1	d2ctza1(c.67.1.3)	d1m32a_(c.67.1.3)	d1v72a1(c.67.1.1)	d1cl1a_(c.67.1.3)	d2ctza1(c.67.1.3)	d1m6sa_(c.67.1.1)
d1oaia_	a.5.2.3	60	18	1	1	d1xb2b1(a.5.2.2)	d1xb2b1(a.5.2.2)	d1vdla_(a.5.2.1)	d1wj7a1(a.5.2.1)	d2dnaa1(a.5.2.1)	d1wgna_(a.5.2.1)
d1ohwa_	c.67.1.4	462	10	1	1	d1m6sa_(c.67.1.1)	d1m6sa_(c.67.1.1)	d1m32a_(c.67.1.3)	d1iuga_(c.67.1.3)	d1u08a_(c.67.1.1)	d1v72a1(c.67.1.1)
d1otra_	a.5.2.4	50	18	1	1	d1wj7a1(a.5.2.1)	d1wj7a1(a.5.2.1)	d2crna1(a.5.2.1)	d1wgna_(a.5.2.1)	d1wiva_(a.5.2.1)	d1veka_(a.5.2.1)
d1pgya_	a.5.2.1	48	5	1	1	d1oaia_(a.5.2.3)	d1v92a_(a.5.2.3)	d1oaia_(a.5.2.3)	d1mn3a_(a.5.2.4)	d1xb2b1(a.5.2.2)	d1otra_(a.5.2.4)
d1q3qa2	c.8.5.2	154	1	1	1	d1kida_(c.8.5.1)	d1kida_(c.8.5.1)	d1a9xb1(c.8.3.1)	d1lqva_(d.19.1.1)	d1kbla2(c.8.1.1)	d1wbha1(c.1.10.1)
d1q6ha_	d.26.1.1	211	1	0	0	d1rypb_(d.153.1.4)	d1kkoa1(c.1.11.2)	d1rypb_(d.153.1.4)	d1rypg_(d.153.1.4)	d1hyrc2(d.19.1.1)	d1b9ha_(c.67.1.4)
d1t11a3	d.26.1.1	114	1	1	1	d2f23a2(d.26.1.2)	d2f23a2(d.26.1.2)	d1rgwa_(b.36.1.1)	d1g9oa_(b.36.1.1)	d1r6ja_(b.36.1.1)	d1rzxa_(b.36.1.1)
d1t16a_	f.4.3.4	428	1	1	1	d1a0tp_(f.4.3.2)	d1a0tp_(f.4.3.2)	d1ejda_(d.68.2.2)	d1g6sa_(d.68.2.2)	d1rf6a_(d.68.2.2)	d2b3ya1(c.8.2.1)
d1ttea1	a.5.2.1	56	5	0	1	d1msza_(d.68.7.1)	d1xb2b1(a.5.2.2)	d1otra_(a.5.2.4)	d1msza_(d.68.7.1)	d1whra_(d.68.7.1)	d1jo0a_(d.68.4.1)
d1u08a_	c.67.1.1	383	8	1	1	d1c7na_(c.67.1.3)	d1m32a_(c.67.1.3)	d1b9ha_(c.67.1.4)	d1c7na_(c.67.1.3)	d1cl1a_(c.67.1.3)	d1iuga_(c.67.1.3)
d1u79a_	d.26.1.1	126	1	0	1	d1uewa_(b.36.1.1)	d2f23a2(d.26.1.2)	d1g9oa_(b.36.1.1)	d1uewa_(b.36.1.1)	d1q3oa_(b.36.1.1)	d1va8a1(b.36.1.1)
d1v72a1	c.67.1.1	346	8	1	1	d1m32a_(c.67.1.3)	d1m32a_(c.67.1.3)	d1c7na_(c.67.1.3)	d1o69a_(c.67.1.4)	d1iuga_(c.67.1.3)	d1b9ha_(c.67.1.4)
d1v92a_	a.5.2.3	47	18	1	1	d2bwba1(a.5.2.1)	d1wgna_(a.5.2.1)	d2bwba1(a.5.2.1)	d1wiva_(a.5.2.1)	d1xb2b1(a.5.2.2)	d2dnaa1(a.5.2.1)
d1vcoa2	c.37.1.10	273	5	0	1	d1ps9a1(c.1.4.1)	d3dhwc1(c.37.1.12)	d1g6oa_(c.37.1.11)	d1rd5a_(c.1.2.4)	d1v72a1(c.67.1.1)	d1ps9a1(c.1.4.1)
d1vdla_	a.5.2.1	81	5	1	1	d1xb2b1(a.5.2.2)	d1otra_(a.5.2.4)	d1v92a_(a.5.2.3)	d1oaia_(a.5.2.3)	d1xb2b1(a.5.2.2)	d1mn3a_(a.5.2.4)
d1vega_	a.5.2.1	84	5	1	1	d1v92a_(a.5.2.3)	d1v92a_(a.5.2.3)	d1oaia_(a.5.2.3)	d1jo0a_(d.68.4.1)	d1dw0a_(a.3.1.1)	d1wvec1(a.3.1.1)
d1veka_	a.5.2.1	85	5	1	1	d1xb2b1(a.5.2.2)	d1xb2b1(a.5.2.2)	d1otra_(a.5.2.4)	d1v92a_(a.5.2.3)	d1oaia_(a.5.2.3)	d1wvec1(a.3.1.1)
d1wgna_	a.5.2.1	64	5	1	1	d1xb2b1(a.5.2.2)	d1v92a_(a.5.2.3)	d1oaia_(a.5.2.3)	d1xb2b1(a.5.2.2)	d1otra_(a.5.2.4)	d1wvec1(a.3.1.1)
d1wiva_	a.5.2.1	74	5	1	1	d1xb2b1(a.5.2.2)	d1oaia_(a.5.2.3)	d1otra_(a.5.2.4)	d1xb2b1(a.5.2.2)	d1v92a_(a.5.2.3)	d1wvec1(a.3.1.1)
d1wj7a1	a.5.2.1	92	5	1	1	d1oaia_(a.5.2.3)	d1otra_(a.5.2.4)	d1xb2b1(a.5.2.2)	d1oaia_(a.5.2.3)	d1v92a_(a.5.2.3)	d1wvec1(a.3.1.1)
d1xb2b1	a.5.2.2	57	19	1	1	d1wiva_(a.5.2.1)	d1wiva_(a.5.2.1)	d1veka_(a.5.2.1)	d2bwba1(a.5.2.1)	d2cpwa1(a.5.2.1)	d2dnaa1(a.5.2.1)
d2b3ya1	c.8.2.1	260	1	0	0	d1i4na_(c.1.2.4)	d1eoka_(c.1.8.5)	d1kbla2(c.8.1.1)	d1vc4a_(c.1.2.4)	d1kida_(c.8.5.1)	d1i4na_(c.1.2.4)
d2bwba1	a.5.2.1	45	5	1	1	d1xb2b1(a.5.2.2)	d1xb2b1(a.5.2.2)	d1mn3a_(a.5.2.4)	d1otra_(a.5.2.4)	d1v92a_(a.5.2.3)	d1oaia_(a.5.2.3)
d2cpwa1	a.5.2.1	52	5	1	1	d1xb2b1(a.5.2.2)	d1xb2b1(a.5.2.2)	d1v92a_(a.5.2.3)	d1oaia_(a.5.2.3)	d1otra_(a.5.2.4)	d1wvec1(a.3.1.1)
d2crna1	a.5.2.1	52	5	1	1	d1xb2b1(a.5.2.2)	d1otra_(a.5.2.4)	d1v92a_(a.5.2.3)	d1xb2b1(a.5.2.2)	d1mn3a_(a.5.2.4)	d1oaia_(a.5.2.3)
d2ctza1	c.67.1.3	422	8	1	1	d1b9ha_(c.67.1.4)	d2r5ea1(c.67.1.1)	d1u08a_(c.67.1.1)	d1b9ha_(c.67.1.4)	d1m6sa_(c.67.1.1)	d1o69a_(c.67.1.4)
d2dkla1	a.5.2.1	73	5	1	1	d1otra_(a.5.2.4)	d1v92a_(a.5.2.3)	d1otra_(a.5.2.4)	d1oaia_(a.5.2.3)	d1msza_(d.68.7.1)	d1hdmb2(d.19.1.1)
d2dnaa1	a.5.2.1	51	5	1	1	d1xb2b1(a.5.2.2)	d1otra_(a.5.2.4)	d1mn3a_(a.5.2.4)	d1v92a_(a.5.2.3)	d1oaia_(a.5.2.3)	d1xb2b1(a.5.2.2)
d2f23a2	d.26.1.2	80	7	1	1	d1hxva_(d.26.1.1)	d1t11a3(d.26.1.1)	d1hxva_(d.26.1.1)	d1g9oa_(b.36.1.1)	d1r6ja_(b.36.1.1)	d1rgwa_(b.36.1.1)
d2hi6a1	c.8.2.3	133	2	0	1	d1kbla2(c.8.1.1)	d1l5ja2(c.8.2.1)	d1t11a3(d.26.1.1)	d1nxja_(c.8.7.1)	d1kbla2(c.8.1.1)	d2d0oa1(c.8.6.1)
d2hoxa1	c.67.1.1	426	8	1	1	d1c7na_(c.67.1.3)	d1b9ha_(c.67.1.4)	d1c7na_(c.67.1.3)	d1m32a_(c.67.1.3)	d2ctza1(c.67.1.3)	d1cl1a_(c.67.1.3)
d2k0bx1	a.5.2.1	53	5	1	1	d1oaia_(a.5.2.3)	d1xb2b1(a.5.2.2)	d1otra_(a.5.2.4)	d1v92a_(a.5.2.3)	d1oaia_(a.5.2.3)	d1mn3a_(a.5.2.4)
d2onka1	c.37.1.12	241	4	1	1	d1g6oa_(c.37.1.11)	d1g6oa_(c.37.1.11)	d1nija1(c.37.1.10)	d1byia_(c.37.1.10)	d1vc4a_(c.1.2.4)	d1ps9a1(c.1.4.1)
d2r5ea1	c.67.1.1	419	8	1	1	d1c7na_(c.67.1.3)	d1iuga_(c.67.1.3)	d1o69a_(c.67.1.4)	d1c7na_(c.67.1.3)	d1b9ha_(c.67.1.4)	d1m32a_(c.67.1.3)
d3dhwc1	c.37.1.12	241	4	1	1	d1g6oa_(c.37.1.11)	d1g6oa_(c.37.1.11)	d1nija1(c.37.1.10)	d1vcoa2(c.37.1.10)	d1djqa1(c.1.4.1)	d1to3a_(c.1.10.1)
d3e46a1	a.5.2.1	43	5	1	1	d1v92a_(a.5.2.3)	d1mn3a_(a.5.2.4)	d1xb2b1(a.5.2.2)	d1v92a_(a.5.2.3)	d1oaia_(a.5.2.3)	d1wvec1(a.3.1.1)
Total Hit				46	51						
Total seq				56	56						
Success Rate				82.14%	91.07%						

Fold											
Name	SCOP ID	Length	Member	Rank1 Hit	Rank5 Hit	Rank1 List	Rank5 List				
d1a9xb1	c.8.3.1	152	11	0	1	d1r6ja_(b.36.1.1)	d2b0aa1(c.8.8.1)	d1q3oa_(b.36.1.1)	d1rgwa_(b.36.1.1)	d1u08a_(c.67.1.1)	d1r6ja_(b.36.1.1)
d1brwa3	d.41.3.1	104	1	0	0	d1wi4a1(b.36.1.1)	d1jdqa_(d.68.3.3)	d2d0oa1(c.8.6.1)	d1wi4a1(b.36.1.1)	d1hdmb2(d.19.1.1)	d2cssa1(b.36.1.1)
d1djqa1	c.1.4.1	341	8	1	1	d1vc4a_(c.1.2.4)	d1wbha1(c.1.10.1)	d1rd5a_(c.1.2.4)	d1kkoa1(c.1.11.2)	d1i4na_(c.1.2.4)	d1vc4a_(c.1.2.4)
d1ejda_	d.68.2.2	420	8	0	0	d1m32a_(c.67.1.3)	d1m6sa_(c.67.1.1)	d1ps9a1(c.1.4.1)	d1iuga_(c.67.1.3)	d1u08a_(c.67.1.1)	d1m32a_(c.67.1.3)
d1eoka_	c.1.8.5	283	9	1	1	d1ps9a1(c.1.4.1)	d1djqa1(c.1.4.1)	d1ps9a1(c.1.4.1)	d2b3ya1(c.8.2.1)	d1lqva_(d.19.1.1)	d2hoxa1(c.67.1.1)
d1g6sa_	d.68.2.2	428	8	0	0	d1m32a_(c.67.1.3)	d1m32a_(c.67.1.3)	d2ctza1(c.67.1.3)	d1u08a_(c.67.1.1)	d1m6sa_(c.67.1.1)	d1iuga_(c.67.1.3)
d1hc7a3	d.68.5.1	75	9	1	1	d2bkya1(d.68.6.1)	d1msza_(d.68.7.1)	d2bkya1(d.68.6.1)	d1kj6a_(g.9.1.1)	d1r6ja_(b.36.1.1)	d1hdmb2(d.19.1.1)
d1hl2a_	c.1.10.1	296	7	1	1	d1rd5a_(c.1.2.4)	d1rd5a_(c.1.2.4)	d1ps9a1(c.1.4.1)	d1i4na_(c.1.2.4)	d1djqa1(c.1.4.1)	d1c7na_(c.67.1.3)
d1i4na_	c.1.2.4	252	7	1	1	d1wbha1(c.1.10.1)	d1djqa1(c.1.4.1)	d1to3a_(c.1.10.1)	d1wbha1(c.1.10.1)	d1hl2a_(c.1.10.1)	d1cl1a_(c.67.1.3)
d1jdqa_	d.68.3.3	99	9	1	1	d1msza_(d.68.7.1)	d1msza_(d.68.7.1)	d1eq3a_(d.26.1.1)	d1uewa_(b.36.1.1)	d1m5ya3(d.26.1.1)	d2f23a2(d.26.1.2)
d1jo0a_	d.68.4.1	98	10	0	1	d1kida_(c.8.5.1)	d1jdqa_(d.68.3.3)	d1ttea1(a.5.2.1)	d1kida_(c.8.5.1)	d1hdma2(d.19.1.1)	d1e29a_(a.3.1.1)
d1kbla2	c.8.1.1	134	11	1	1	d2hi6a1(c.8.2.3)	d2isba1(c.8.9.1)	d1nxja_(c.8.7.1)	d2hi6a1(c.8.2.3)	d1a9xb1(c.8.3.1)	d2b3ya1(c.8.2.1)
d1kida_	c.8.5.1	194	10	0	1	d1i4na_(c.1.2.4)	d2d0oa1(c.8.6.1)	d1nija1(c.37.1.10)	d1jj7a_(c.37.1.12)	d1i4na_(c.1.2.4)	d1wbha1(c.1.10.1)
d1kkoa1	c.1.11.2	252	9	1	1	d1wbha1(c.1.10.1)	d1djqa1(c.1.4.1)	d1to3a_(c.1.10.1)	d1wbha1(c.1.10.1)	d1i4na_(c.1.2.4)	d1byia_(c.37.1.10)
d1l5ja2	c.8.2.1	213	9	1	1	d1kbla2(c.8.1.1)	d1nxja_(c.8.7.1)	d2d0oa1(c.8.6.1)	d1kbla2(c.8.1.1)	d1wbha1(c.1.10.1)	d1g6oa_(c.37.1.11)
d1msza_	d.68.7.1	63	9	0	1	d1ttea1(a.5.2.1)	d1nj8a2(d.68.5.1)	d1pava_(d.68.3.3)	d1jo0a_(d.68.4.1)	d1jdqa_(d.68.3.3)	d1ttea1(a.5.2.1)
d1nj8a2	d.68.5.1	63	9	0	1	d1eq3a_(d.26.1.1)	d1msza_(d.68.7.1)	d2bkya1(d.68.6.1)	d1eq3a_(d.26.1.1)	d2f23a2(d.26.1.2)	d1m5ya3(d.26.1.1)
d1nxja_	c.8.7.1	157	11	1	1	d1kbla2(c.8.1.1)	d2b3ya1(c.8.2.1)	d1kbla2(c.8.1.1)	d1r61a_(c.8.8.1)	d2isba1(c.8.9.1)	d1byia_(c.37.1.10)
d1pava_	d.68.3.3	79	9	1	1	d1msza_(d.68.7.1)	d1msza_(d.68.7.1)	d1eq3a_(d.26.1.1)	d1hdmb2(d.19.1.1)	d1r6ja_(b.36.1.1)	d1hdma2(d.19.1.1)
d1ps9a1	c.1.4.1	331	8	1	1	d1rd5a_(c.1.2.4)	d1vc4a_(c.1.2.4)	d1to3a_(c.1.10.1)	d1hl2a_(c.1.10.1)	d1i4na_(c.1.2.4)	d1rd5a_(c.1.2.4)
d1q3qa2	c.8.5.2	154	10	1	1	d1kbla2(c.8.1.1)	d1a9xb1(c.8.3.1)	d2d0oa1(c.8.6.1)	d1kbla2(c.8.1.1)	d1lqva_(d.19.1.1)	d1wbha1(c.1.10.1)
d1r61a_	c.8.8.1	206	10	0	1	d1q3oa_(b.36.1.1)	d1a9xb1(c.8.3.1)	d2b3ya1(c.8.2.1)	d2hi6a1(c.8.2.3)	d1nxja_(c.8.7.1)	d1q3oa_(b.36.1.1)
d1rd5a_	c.1.2.4	262	7	1	1	d1wbha1(c.1.10.1)	d1to3a_(c.1.10.1)	d1hl2a_(c.1.10.1)	d1ps9a1(c.1.4.1)	d1wbha1(c.1.10.1)	d1djqa1(c.1.4.1)
d1rf6a_	d.68.2.2	428	8	0	0	d1m32a_(c.67.1.3)	d1u08a_(c.67.1.1)	d1m32a_(c.67.1.3)	d2ctza1(c.67.1.3)	d1iuga_(c.67.1.3)	d1cl1a_(c.67.1.3)
d1to3a_	c.1.10.1	292	7	1	1	d1rd5a_(c.1.2.4)	d1rd5a_(c.1.2.4)	d1vc4a_(c.1.2.4)	d1ps9a1(c.1.4.1)	d1i4na_(c.1.2.4)	d1kkoa1(c.1.11.2)
d1vc4a_	c.1.2.4	255	7	1	1	d1wbha1(c.1.10.1)	d1to3a_(c.1.10.1)	d1djqa1(c.1.4.1)	d1kkoa1(c.1.11.2)	d1ps9a1(c.1.4.1)	d1wbha1(c.1.10.1)
d1wbha1	c.1.10.1	214	7	1	1	d1vc4a_(c.1.2.4)	d1kkoa1(c.1.11.2)	d1rd5a_(c.1.2.4)	d1vc4a_(c.1.2.4)	d1djqa1(c.1.4.1)	d1i4na_(c.1.2.4)
d1whra_	d.68.7.1	125	9	0	1	d1kida_(c.8.5.1)	d1jo0a_(d.68.4.1)	d1e29a_(a.3.1.1)	d1kida_(c.8.5.1)	d1i8oa_(a.3.1.1)	d1hdma2(d.19.1.1)
d2b0aa1	c.8.8.1	187	10	1	1	d1a9xb1(c.8.3.1)	d1a9xb1(c.8.3.1)	d2b3ya1(c.8.2.1)	d2d0oa1(c.8.6.1)	d1kbla2(c.8.1.1)	d1u08a_(c.67.1.1)
d2b3ya1	c.8.2.1	260	9	0	1	d1i4na_(c.1.2.4)	d1kbla2(c.8.1.1)	d1kida_(c.8.5.1)	d1eoka_(c.1.8.5)	d1vc4a_(c.1.2.4)	d1i4na_(c.1.2.4)
d2bkya1	d.68.6.1	90	10	1	1	d1hc7a3(d.68.5.1)	d1hc7a3(d.68.5.1)	d1nj8a2(d.68.5.1)	d1hxva_(d.26.1.1)	d1q3oa_(b.36.1.1)	d1r6ja_(b.36.1.1)
d2d0oa1	c.8.6.1	163	11	1	1	d2hi6a1(c.8.2.3)	d2hi6a1(c.8.2.3)	d1kida_(c.8.5.1)	d1nija1(c.37.1.10)	d1byia_(c.37.1.10)	d2f23a2(d.26.1.2)
d2gyck1	d.41.4.2	132	2	0	0	d2cssa1(b.36.1.1)	d1rzxa_(b.36.1.1)	d2cssa1(b.36.1.1)	d1wi4a1(b.36.1.1)	d1tp5a1(b.36.1.1)	d1va8a1(b.36.1.1)
d2hi6a1	c.8.2.3	133	9	1	1	d1kbla2(c.8.1.1)	d1nxja_(c.8.7.1)	d1kbla2(c.8.1.1)	d2d0oa1(c.8.6.1)	d1t11a3(d.26.1.1)	d2cssa1(b.36.1.1)
d2isba1	c.8.9.1	179	11	1	1	d1nxja_(c.8.7.1)	d1q3qa2(c.8.5.2)	d1nxja_(c.8.7.1)	d1kbla2(c.8.1.1)	d1m32a_(c.67.1.3)	d1rf6a_(d.68.2.2)
d2tpta3	d.41.3.1	106	1	0	1	d1wi4a1(b.36.1.1)	d2gyck1(d.41.4.2)	d1tp5a1(b.36.1.1)	d1nj8a2(d.68.5.1)	d1wi4a1(b.36.1.1)	d1hdmb2(d.19.1.1)
Total Hit				22	31						
Total seq				36	36						
Success Rate				61.11%	86.11%						

Prediction results on SCOP_TEST by RF-Fold

Family											
Name	SCOP ID	Length	Member	Rank1 Hit	Rank5 Hit	Rank1 List	Rank5 List				
d1b9ha_	c.67.1.4	385	2	1	1	d1o69a_(c.67.1.4)	d1o69a_(c.67.1.4)	d1u08a_(c.67.1.1)	d1m6sa_(c.67.1.1)	d1cl1a_(c.67.1.3)	d2ctza1(c.67.1.3)
d1brwa3	d.41.3.1	104	1	1	1	d2tpta3(d.41.3.1)	d2tpta3(d.41.3.1)	d1wi4a1(b.36.1.1)	d2cssa1(b.36.1.1)	d1kbla2(c.8.1.1)	d1nj8a2(d.68.5.1)
d1byia_	c.37.1.10	225	2	1	1	d1nija1(c.37.1.10)	d1vcoa2(c.37.1.10)	d1nija1(c.37.1.10)	d1hl2a_(c.1.10.1)	d1u08a_(c.67.1.1)	d1jj7a_(c.37.1.12)
d1c7na_	c.67.1.3	395	4	0	0	d2r5ea1(c.67.1.1)	d1v72a1(c.67.1.1)	d1m6sa_(c.67.1.1)	d2r5ea1(c.67.1.1)	d2hoxa1(c.67.1.1)	d1u08a_(c.67.1.1)
d1cl1a_	c.67.1.3	392	4	1	1	d2ctza1(c.67.1.3)	d1m32a_(c.67.1.3)	d1iuga_(c.67.1.3)	d2ctza1(c.67.1.3)	d2r5ea1(c.67.1.1)	d1b9ha_(c.67.1.4)
d1ctja_	a.3.1.1	90	6	1	1	d1wvec1(a.3.1.1)	d2c8sa1(a.3.1.1)	d1gu2a_(a.3.1.1)	d1wvec1(a.3.1.1)	d1i8oa_(a.3.1.1)	d1e29a_(a.3.1.1)
d1djqa1	c.1.4.1	341	1	1	1	d1ps9a1(c.1.4.1)	d1ps9a1(c.1.4.1)	d1wbha1(c.1.10.1)	d1rd5a_(c.1.2.4)	d1kkoa1(c.1.11.2)	d1hl2a_(c.1.10.1)
d1dw0a_	a.3.1.1	113	6	1	1	d1gu2a_(a.3.1.1)	d1gu2a_(a.3.1.1)	d2c8sa1(a.3.1.1)	d1e29a_(a.3.1.1)	d1i8oa_(a.3.1.1)	d1ctja_(a.3.1.1)
d1e29a_	a.3.1.1	136	6	1	1	d1ctja_(a.3.1.1)	d1wvec1(a.3.1.1)	d2c8sa1(a.3.1.1)	d1i8oa_(a.3.1.1)	d1ctja_(a.3.1.1)	d1gu2a_(a.3.1.1)
d1ejda_	d.68.2.2	420	2	1	1	d1g6sa_(d.68.2.2)	d1g6sa_(d.68.2.2)	d1rf6a_(d.68.2.2)	d1m6sa_(c.67.1.1)	d1cl1a_(c.67.1.3)	d1kida_(c.8.5.1)
d1eq3a_	d.26.1.1	97	6	1	1	d1m5ya3(d.26.1.1)	d1t11a3(d.26.1.1)	d1m5ya3(d.26.1.1)	d1hxva_(d.26.1.1)	d1pava_(d.68.3.3)	d1vaea_(b.36.1.1)
d1ewsa_	g.9.1.1	33	3	1	1	d1ijva_(g.9.1.1)	d1ijva_(g.9.1.1)	d1kj6a_(g.9.1.1)	d1zuea1(g.9.1.1)	d1nija1(c.37.1.10)	d1jvwa_(d.26.1.1)
d1g3ka_	d.153.1.4	174	7	1	1	d1ryph_(d.153.1.4)	d1ryph_(d.153.1.4)	d1rypg_(d.153.1.4)	d1rype_(d.153.1.4)	d1ryp2_(d.153.1.4)	d1rypc_(d.153.1.4)
d1g6sa_	d.68.2.2	428	2	1	1	d1ejda_(d.68.2.2)	d1ejda_(d.68.2.2)	d1rf6a_(d.68.2.2)	d1m32a_(c.67.1.3)	d1iuga_(c.67.1.3)	d1cl1a_(c.67.1.3)

Superfamily											
Name	SCOP ID	Length	Member	Rank1 Hit	Rank5 Hit	Rank1 List	Rank5 List				
d1a0tp_	f.4.3.2	414	1	1	1	d1t16a_(f.4.3.4)	d1t16a_(f.4.3.4)	d1a9xb1(c.8.3.1)	d1eoka_(c.1.8.5)	d2b0aa1(c.8.8.1)	d1nxja_(c.8.7.1)
d1b9ha_	c.67.1.4	385	10	1	1	d1m6sa_(c.67.1.1)	d2r5ea1(c.67.1.1)	d1u08a_(c.67.1.1)	d1m6sa_(c.67.1.1)	d1cl1a_(c.67.1.3)	d2ctza1(c.67.1.3)
d1byia_	c.37.1.10	225	5	1	1	d1jj7a_(c.37.1.12)	d1jj7a_(c.37.1.12)	d1m32a_(c.67.1.3)	d1g6sa_(d.68.2.2)	d1hl2a_(c.1.10.1)	d1u08a_(c.67.1.1)
d1c7na_	c.67.1.3	395	8	1	1	d2r5ea1(c.67.1.1)	d1v72a1(c.67.1.1)	d1m6sa_(c.67.1.1)	d2r5ea1(c.67.1.1)	d2hoxa1(c.67.1.1)	d1u08a_(c.67.1.1)
d1cl1a_	c.67.1.3	392	8	1	1	d1b9ha_(c.67.1.4)	d1u08a_(c.67.1.1)	d2r5ea1(c.67.1.1)	d1m6sa_(c.67.1.1)	d1b9ha_(c.67.1.4)	d1o69a_(c.67.1.4)
d1eq3a_	d.26.1.1	97	1	0	0	d1pava_(d.68.3.3)	d1jdqa_(d.68.3.3)	d1r6ja_(b.36.1.1)	d1pava_(d.68.3.3)	d1vaea_(b.36.1.1)	d1nj8a2(d.68.5.1)
d1g6oa_	c.37.1.11	324	7	1	1	d1jj7a_(c.37.1.12)	d2onka1(c.37.1.12)	d1l2ta_(c.37.1.12)	d1jj7a_(c.37.1.12)	d3dhwc1(c.37.1.12)	d1nija1(c.37.1.10)
d1hxva_	d.26.1.1	86	1	1	1	d2f23a2(d.26.1.2)	d2f23a2(d.26.1.2)	d1q3oa_(b.36.1.1)	d1vaea_(b.36.1.1)	d1jdqa_(d.68.3.3)	d1g9oa_(b.36.1.1)
d1iuga_	c.67.1.3	349	8	1	1	d1v72a1(c.67.1.1)	d2r5ea1(c.67.1.1)	d1v72a1(c.67.1.1)	d1m6sa_(c.67.1.1)	d1o69a_(c.67.1.4)	d1b9ha_(c.67.1.4)
d1jj7a_	c.37.1.12	252	4	1	1	d1g6oa_(c.37.1.11)	d1nija1(c.37.1.10)	d1g6oa_(c.37.1.11)	d1byia_(c.37.1.10)	d1ps9a1(c.1.4.1)	d1iuga_(c.67.1.3)
d1jvwa_	d.26.1.1	161	1	0	0	d1pava_(d.68.3.3)	d1a9xb1(c.8.3.1)	d1pava_(d.68.3.3)	d1va8a1(b.36.1.1)	d2tpta3(d.41.3.1)	d1yara1(d.153.1.4)
d1kida_	c.8.5.1	194	1	1	1	d1q3qa2(c.8.5.2)	d1q3qa2(c.8.5.2)	d1nija1(c.37.1.10)	d1ejda_(d.68.2.2)	d1wbha1(c.1.10.1)	d1hl2a_(c.1.10.1)
d1l2ta_	c.37.1.12	231	4	1	1	d1g6oa_(c.37.1.11)	d1g6oa_(c.37.1.11)	d1nija1(c.37.1.10)	d1vcoa2(c.37.1.10)	d1byia_(c.37.1.10)	d1kida_(c.8.5.1)
d1l5ja2	c.8.2.1	213	1	1	1	d2hi6a1(c.8.2.3)	d2hi6a1(c.8.2.3)	d1iuga_(c.67.1.3)	d1nxja_(c.8.7.1)	d2d0oa1(c.8.6.1)	d1kbla2(c.8.1.1)

d1g9oa_	b.36.1.1	92	10	1	1	d1rzxa_(b.36.1.1)	d1rzxa_(b.36.1.1)	d1q3oa_(b.36.1.1)	d1uewa_(b.36.1.1)	d1r6ja_(b.36.1.1)	d1wi4a1(b.36.1.1)
d1gu2a_	a.3.1.1	125	6	1	1	d1dw0a_(a.3.1.1)	d1i8oa_(a.3.1.1)	d1e29a_(a.3.1.1)	d2c8sa1(a.3.1.1)	d1ctja_(a.3.1.1)	d1dw0a_(a.3.1.1)
d1hc7a3	d.68.5.1	75	1	1	1	d1nj8a2(d.68.5.1)	d1nj8a2(d.68.5.1)	d1nija1(c.37.1.10)	d1rgwa_(b.36.1.1)	d1ewsa_(g.9.1.1)	d1pava_(d.68.3.3)
d1hdma2	d.19.1.1	82	6	1	1	d1lqva_(d.19.1.1)	d1hyrc2(d.19.1.1)	d1lqva_(d.19.1.1)	d2h26a2(d.19.1.1)	d1pava_(d.68.3.3)	d1vaea_(b.36.1.1)
d1hdmb2	d.19.1.1	86	6	1	1	d1uvqb2(d.19.1.1)	d1hdma2(d.19.1.1)	d1uvqb2(d.19.1.1)	d1hyrc2(d.19.1.1)	d1lqva_(d.19.1.1)	d1pava_(d.68.3.3)
d1hl2a_	c.1.10.1	296	2	0	1	d1ps9a1(c.1.4.1)	d1wbha1(c.1.10.1)	d1m32a_(c.67.1.3)	d1rd5a_(c.1.2.4)	d1ps9a1(c.1.4.1)	d1djqa1(c.1.4.1)
d1hxva_	d.26.1.1	86	6	1	1	d1u79a_(d.26.1.1)	d1eq3a_(d.26.1.1)	d1jvwa_(d.26.1.1)	d1u79a_(d.26.1.1)	d1t11a3(d.26.1.1)	d1q6ha_(d.26.1.1)
d1hyrc2	d.19.1.1	182	6	1	1	d1t7va2(d.19.1.1)	d1uvqb2(d.19.1.1)	d1t7va2(d.19.1.1)	d1lqva_(d.19.1.1)	d2h26a2(d.19.1.1)	d1eoka_(c.1.8.5)
d1i4na_	c.1.2.4	252	2	1	1	d1vc4a_(c.1.2.4)	d1rd5a_(c.1.2.4)	d1vc4a_(c.1.2.4)	d1djqa1(c.1.4.1)	d1to3a_(c.1.10.1)	d1wbha1(c.1.10.1)
d1i8oa_	a.3.1.1	115	6	1	1	d1ctja_(a.3.1.1)	d1wvec1(a.3.1.1)	d1e29a_(a.3.1.1)	d1dw0a_(a.3.1.1)	d2c8sa1(a.3.1.1)	d1ctja_(a.3.1.1)
d1ijva_	g.9.1.1	37	3	1	1	d1kj6a_(g.9.1.1)	d1kj6a_(g.9.1.1)	d1zuea1(g.9.1.1)	d1ewsa_(g.9.1.1)	d1whra_(d.68.7.1)	d1nj8a2(d.68.5.1)
d1iuga_	c.67.1.3	349	4	1	1	d1m32a_(c.67.1.3)	d1cl1a_(c.67.1.3)	d1m32a_(c.67.1.3)	d1v72a1(c.67.1.1)	d1m6sa_(c.67.1.1)	d1o69a_(c.67.1.4)
d1jdqa_	d.68.3.3	99	1	1	1	d1pava_(d.68.3.3)	d1pava_(d.68.3.3)	d1msza_(d.68.7.1)	d1r6ja_(b.36.1.1)	d1eq3a_(d.26.1.1)	d1hxva_(d.26.1.1)
d1jj7a_	c.37.1.12	252	3	1	1	d1l2ta_(c.37.1.12)	d3dhwc1(c.37.1.12)	d2onka1(c.37.1.12)	d1l2ta_(c.37.1.12)	d1nija1(c.37.1.10)	d1g6oa_(c.37.1.11)
d1jvwa_	d.26.1.1	161	6	1	1	d1u79a_(d.26.1.1)	d1u79a_(d.26.1.1)	d1t11a3(d.26.1.1)	d1q6ha_(d.26.1.1)	d1eq3a_(d.26.1.1)	d1hxva_(d.26.1.1)
d1kj6a_	g.9.1.1	46	3	1	1	d1ijva_(g.9.1.1)	d1zuea1(g.9.1.1)	d1ewsa_(g.9.1.1)	d1ijva_(g.9.1.1)	d1nj8a2(d.68.5.1)	d1hc7a3(d.68.5.1)
d1l2ta_	c.37.1.12	231	3	1	1	d3dhwc1(c.37.1.12)	d3dhwc1(c.37.1.12)	d1jj7a_(c.37.1.12)	d2onka1(c.37.1.12)	d1g6oa_(c.37.1.11)	d1nija1(c.37.1.10)
d1l5ja2	c.8.2.1	213	1	1	1	d2b3ya1(c.8.2.1)	d2b3ya1(c.8.2.1)	d1iuga_(c.67.1.3)	d2hi6a1(c.8.2.3)	d1nxja_(c.8.7.1)	d1rd5a_(c.1.2.4)
d1lqva_	d.19.1.1	173	6	1	1	d2h26a2(d.19.1.1)	d2h26a2(d.19.1.1)	d1t7va2(d.19.1.1)	d1uvqb2(d.19.1.1)	d1hyrc2(d.19.1.1)	d1hdmb2(d.19.1.1)
d1m32a_	c.67.1.3	362	4	1	1	d1iuga_(c.67.1.3)	d1iuga_(c.67.1.3)	d2ctza1(c.67.1.3)	d1m6sa_(c.67.1.1)	d1b9ha_(c.67.1.4)	d1v72a1(c.67.1.1)
d1m5ya3	d.26.1.1	109	6	1	1	d1eq3a_(d.26.1.1)	d1hxva_(d.26.1.1)	d1t11a3(d.26.1.1)	d1eq3a_(d.26.1.1)	d1pava_(d.68.3.3)	d1jdqa_(d.68.3.3)
d1m6sa_	c.67.1.1	344	4	1	1	d1v72a1(c.67.1.1)	d1u08a_(c.67.1.1)	d1v72a1(c.67.1.1)	d1o69a_(c.67.1.4)	d1m32a_(c.67.1.3)	d1ohwa_(c.67.1.4)
d1mn3a_	a.5.2.4	55	1	1	1	d1otra_(a.5.2.4)	d1otra_(a.5.2.4)	d2bwba1(a.5.2.1)	d3e46a1(a.5.2.1)	d2k0bx1(a.5.2.1)	d1wgna_(a.5.2.1)
d1msza_	d.68.7.1	63	1	1	1	d1whra_(d.68.7.1)	d1whra_(d.68.7.1)	d2k0bx1(a.5.2.1)	d2bkya1(d.68.6.1)	d1pava_(d.68.3.3)	d2crna1(a.5.2.1)
d1nija1	c.37.1.10	223	2	1	1	d1byia_(c.37.1.10)	d1byia_(c.37.1.10)	d2onka1(c.37.1.12)	d1g6oa_(c.37.1.11)	d3dhwc1(c.37.1.12)	d1l2ta_(c.37.1.12)
d1nj8a2	d.68.5.1	63	1	1	1	d1hc7a3(d.68.5.1)	d1hc7a3(d.68.5.1)	d1whra_(d.68.7.1)	d1msza_(d.68.7.1)	d1m5ya3(d.26.1.1)	d2bkya1(d.68.6.1)
d1o69a_	c.67.1.4	375	2	1	1	d1b9ha_(c.67.1.4)	d1b9ha_(c.67.1.4)	d1m32a_(c.67.1.3)	d1cl1a_(c.67.1.3)	d2ctza1(c.67.1.3)	d1m6sa_(c.67.1.1)
d1oaia_	a.5.2.3	60	1	1	1	d1v92a_(a.5.2.3)	d1v92a_(a.5.2.3)	d1xb2b1(a.5.2.2)	d1wj7a1(a.5.2.1)	d2dnaa1(a.5.2.1)	d1wgna_(a.5.2.1)
d1ohwa_	c.67.1.4	462	2	0	0	d1m6sa_(c.67.1.1)	d1m6sa_(c.67.1.1)	d1m32a_(c.67.1.3)	d1iuga_(c.67.1.3)	d1c7na_(c.67.1.3)	d1v72a1(c.67.1.1)
d1otra_	a.5.2.4	50	1	0	1	d1wj7a1(a.5.2.1)	d1mn3a_(a.5.2.4)	d1wj7a1(a.5.2.1)	d2bwba1(a.5.2.1)	d2crna1(a.5.2.1)	d1wiva_(a.5.2.1)
d1pava_	d.68.3.3	79	1	1	1	d1jdqa_(d.68.3.3)	d1jdqa_(d.68.3.3)	d1hxva_(d.26.1.1)	d1eq3a_(d.26.1.1)	d1msza_(d.68.7.1)	d1m5ya3(d.26.1.1)
d1pgya_	a.5.2.1	48	14	1	1	d1wgna_(a.5.2.1)	d1wiva_(a.5.2.1)	d1veka_(a.5.2.1)	d1wgna_(a.5.2.1)	d3e46a1(a.5.2.1)	d2dnaa1(a.5.2.1)
d1ps9a1	c.1.4.1	331	1	1	1	d1djqa1(c.1.4.1)	d1djqa1(c.1.4.1)	d1vc4a_(c.1.2.4)	d1to3a_(c.1.10.1)	d1hl2a_(c.1.10.1)	d1rd5a_(c.1.2.4)
d1q3oa_	b.36.1.1	105	10	1	1	d1vaea_(b.36.1.1)	d1rzxa_(b.36.1.1)	d1r6ja_(b.36.1.1)	d1vaea_(b.36.1.1)	d2cssa1(b.36.1.1)	d1wi4a1(b.36.1.1)
d1q6ha_	d.26.1.1	211	6	1	1	d1jvwa_(d.26.1.1)	d1t11a3(d.26.1.1)	d1jvwa_(d.26.1.1)	d1u79a_(d.26.1.1)	d1hxva_(d.26.1.1)	d1rypg_(d.153.1.4)
d1r61a_	c.8.8.1	206	1	1	1	d2b0aa1(c.8.8.1)	d2b0aa1(c.8.8.1)	d2r5ea1(c.67.1.1)	d1nxja_(c.8.7.1)	d1ps9a1(c.1.4.1)	d1m32a_(c.67.1.3)
d1r6ja_	b.36.1.1	83	10	1	1	d1tp5a1(b.36.1.1)	d1tp5a1(b.36.1.1)	d1rzxa_(b.36.1.1)	d1rgwa_(b.36.1.1)	d2cssa1(b.36.1.1)	d1g9oa_(b.36.1.1)
d1rd5a_	c.1.2.4	262	2	1	1	d1i4na_(c.1.2.4)	d1i4na_(c.1.2.4)	d1vc4a_(c.1.2.4)	d1to3a_(c.1.10.1)	d1wbha1(c.1.10.1)	d1djqa1(c.1.4.1)
d1rf6a_	d.68.2.2	428	2	1	1	d1ejda_(d.68.2.2)	d1ejda_(d.68.2.2)	d1g6sa_(d.68.2.2)	d1m32a_(c.67.1.3)	d1hl2a_(c.1.10.1)	d2ctza1(c.67.1.3)
d1rgwa_	b.36.1.1	86	10	1	1	d1tp5a1(b.36.1.1)	d1tp5a1(b.36.1.1)	d1r6ja_(b.36.1.1)	d1va8a1(b.36.1.1)	d1g9oa_(b.36.1.1)	d2cssa1(b.36.1.1)
d1ryp2_	d.153.1.4	234	7	1	1	d1rypc_(d.153.1.4)	d1rypc_(d.153.1.4)	d1ryph_(d.153.1.4)	d1rypg_(d.153.1.4)	d1g3ka_(d.153.1.4)	d1rypb_(d.153.1.4)
d1rypb_	d.153.1.4	251	7	1	1	d1rypc_(d.153.1.4)	d1ryp2_(d.153.1.4)	d1rypc_(d.153.1.4)	d1rype_(d.153.1.4)	d1rypg_(d.153.1.4)	d1g3ka_(d.153.1.4)
d1rypc_	d.153.1.4	245	7	1	1	d1yara1(d.153.1.4)	d1yara1(d.153.1.4)	d1ryp2_(d.153.1.4)	d1rypg_(d.153.1.4)	d1rypb_(d.153.1.4)	d1ryph_(d.153.1.4)
d1rype_	d.153.1.4	243	7	1	1	d1rypg_(d.153.1.4)	d1rypg_(d.153.1.4)	d1g3ka_(d.153.1.4)	d1ryp2_(d.153.1.4)	d1yara1(d.153.1.4)	d1ryph_(d.153.1.4)
d1rypg_	d.153.1.4	245	7	1	1	d1yara1(d.153.1.4)	d1yara1(d.153.1.4)	d1rype_(d.153.1.4)	d1rypc_(d.153.1.4)	d1ryp2_(d.153.1.4)	d1g3ka_(d.153.1.4)
d1ryph_	d.153.1.4	206	7	1	1	d1rypb_(d.153.1.4)	d1ryp2_(d.153.1.4)	d1yara1(d.153.1.4)	d1rype_(d.153.1.4)	d1g3ka_(d.153.1.4)	d1rypc_(d.153.1.4)
d1rzxa_	b.36.1.1	99	10	1	1	d1tp5a1(b.36.1.1)	d1q3oa_(b.36.1.1)	d1tp5a1(b.36.1.1)	d1g9oa_(b.36.1.1)	d1wi4a1(b.36.1.1)	d1uewa_(b.36.1.1)
d1t11a3	d.26.1.1	114	6	1	1	d1hxva_(d.26.1.1)	d1q6ha_(d.26.1.1)	d1hxva_(d.26.1.1)	d1u79a_(d.26.1.1)	d1jvwa_(d.26.1.1)	d2f23a2(d.26.1.2)
d1t7va2	d.19.1.1	180	6	1	1	d1hyrc2(d.19.1.1)	d2h26a2(d.19.1.1)	d1uvqb2(d.19.1.1)	d1hyrc2(d.19.1.1)	d1lqva_(d.19.1.1)	d1eoka_(c.1.8.5)
d1to3a_	c.1.10.1	292	2	0	1	d1i4na_(c.1.2.4)	d1wbha1(c.1.10.1)	d1rd5a_(c.1.2.4)	d1vc4a_(c.1.2.4)	d1ps9a1(c.1.4.1)	d1i4na_(c.1.2.4)
d1tp5a1	b.36.1.1	103	10	1	1	d1q3oa_(b.36.1.1)	d1q3oa_(b.36.1.1)	d1rgwa_(b.36.1.1)	d1va8a1(b.36.1.1)	d1g9oa_(b.36.1.1)	d1r6ja_(b.36.1.1)
d1ttea1	a.5.2.1	56	14	1	1	d2cpwa1(a.5.2.1)	d1veka_(a.5.2.1)	d1wgna_(a.5.2.1)	d1wiva_(a.5.2.1)	d2cpwa1(a.5.2.1)	d2crna1(a.5.2.1)
d1u08a_	c.67.1.1	383	4	1	1	d2r5ea1(c.67.1.1)	d2r5ea1(c.67.1.1)	d1m6sa_(c.67.1.1)	d2hoxa1(c.67.1.1)	d1b9ha_(c.67.1.4)	d1c7na_(c.67.1.3)
d1u79a_	d.26.1.1	126	6	1	1	d1jvwa_(d.26.1.1)	d1jvwa_(d.26.1.1)	d1q6ha_(d.26.1.1)	d1hxva_(d.26.1.1)	d1t11a3(d.26.1.1)	d1tp5a1(b.36.1.1)
d1uewa_	b.36.1.1	115	10	1	1	d2cssa1(b.36.1.1)	d1g9oa_(b.36.1.1)	d1rgwa_(b.36.1.1)	d2cssa1(b.36.1.1)	d1vaea_(b.36.1.1)	d1wi4a1(b.36.1.1)
d1uvqb2	d.19.1.1	93	6	1	1	d1lqva_(d.19.1.1)	d1t7va2(d.19.1.1)	d1hdmb2(d.19.1.1)	d1lqva_(d.19.1.1)	d1hyrc2(d.19.1.1)	d1tp5a1(b.36.1.1)
d1v72a1	c.67.1.1	346	4	1	1	d1m6sa_(c.67.1.1)	d1m6sa_(c.67.1.1)	d1u08a_(c.67.1.1)	d1m32a_(c.67.1.3)	d1iuga_(c.67.1.3)	d1b9ha_(c.67.1.4)
d1v92a_	a.5.2.3	47	1	1	1	d1oaia_(a.5.2.3)	d1oaia_(a.5.2.3)	d2bwba1(a.5.2.1)	d1wiva_(a.5.2.1)	d1wj7a1(a.5.2.1)	d1xb2b1(a.5.2.2)
d1va8a1	b.36.1.1	101	10	1	1	d2cssa1(b.36.1.1)	d2cssa1(b.36.1.1)	d1r6ja_(b.36.1.1)	d1rgwa_(b.36.1.1)	d1wi4a1(b.36.1.1)	d1vaea_(b.36.1.1)
d1vaea_	b.36.1.1	112	10	1	1	d1tp5a1(b.36.1.1)	d1uewa_(b.36.1.1)	d1tp5a1(b.36.1.1)	d1rgwa_(b.36.1.1)	d1g9oa_(b.36.1.1)	d1q3oa_(b.36.1.1)
d1vc4a_	c.1.2.4	255	2	1	1	d1i4na_(c.1.2.4)	d1i4na_(c.1.2.4)	d1rd5a_(c.1.2.4)	d1to3a_(c.1.10.1)	d1ps9a1(c.1.4.1)	d1wbha1(c.1.10.1)
d1vcoa2	c.37.1.10	273	2	1	1	d1byia_(c.37.1.10)	d1byia_(c.37.1.10)	d3dhwc1(c.37.1.12)	d1ps9a1(c.1.4.1)	d1m32a_(c.67.1.3)	d1rf6a_(d.68.2.2)
d1vdla_	a.5.2.1	81	14	1	1	d1wj7a1(a.5.2.1)	d1wgna_(a.5.2.1)	d1wj7a1(a.5.2.1)	d2bwba1(a.5.2.1)	d2dnaa1(a.5.2.1)	d1xb2b1(a.5.2.2)
d1vega_	a.5.2.1	84	14	1	1	d1wiva_(a.5.2.1)	d2cpwa1(a.5.2.1)	d1wiva_(a.5.2.1)	d1veka_(a.5.2.1)	d1wgna_(a.5.2.1)	d2bwba1(a.5.2.1)
d1veka_	a.5.2.1	85	14	1	1	d2crna1(a.5.2.1)	d1wiva_(a.5.2.1)	d1vega_(a.5.2.1)	d2cpwa1(a.5.2.1)	d2crna1(a.5.2.1)	d1wgna_(a.5.2.1)
d1wbha1	c.1.10.1	214	2	0	1	d1vc4a_(c.1.2.4)	d1to3a_(c.1.10.1)	d1rd5a_(c.1.2.4)	d1ps9a1(c.1.4.1)	d1vc4a_(c.1.2.4)	d1i4na_(c.1.2.4)
d1wgna_	a.5.2.1	64	14	1	1	d1veka_(a.5.2.1)	d3e46a1(a.5.2.1)	d2cpwa1(a.5.2.1)	d1wiva_(a.5.2.1)	d2crna1(a.5.2.1)	d1veka_(a.5.2.1)
d1whra_	d.68.7.1	125	1	1	1	d1msza_(d.68.7.1)	d1msza_(d.68.7.1)	d1l2ta_(c.37.1.12)	d1ttea1(a.5.2.1)	d1q3qa2(c.8.5.2)	d1gu2a_(a.3.1.1)
d1wi4a1	b.36.1.1	97	10	1	1	d1tp5a1(b.36.1.1)	d1g9oa_(b.36.1.1)	d1rgwa_(b.36.1.1)	d1tp5a1(b.36.1.1)	d1va8a1(b.36.1.1)	d1rzxa_(b.36.1.1)
d1wiva_	a.5.2.1	74	14	1	1	d1wgna_(a.5.2.1)	d1wgna_(a.5.2.1)	d1veka_(a.5.2.1)	d2crna1(a.5.2.1)	d2cpwa1(a.5.2.1)	d2bwba1(a.5.2.1)
d1wj7a1	a.5.2.1	92	14	1	1	d1wiva_(a.5.2.1)	d1vdla_(a.5.2.1)	d1wiva_(a.5.2.1)	d2dnaa1(a.5.2.1)	d1veka_(a.5.2.1)	d2bwba1(a.5.2.1)
d1wvec1	a.3.1.1	75	6	1	1	d2c8sa1(a.3.1.1)	d1dw0a_(a.3.1.1)	d1e29a_(a.3.1.1)	d1i8oa_(a.3.1.1)	d1ctja_(a.3.1.1)	d2c8sa1(a.3.1.1)
d1yara1	d.153.1.4	222	7	1	1	d1rype_(d.153.1.4)	d1g3ka_(d.153.1.4)	d1rype_(d.153.1.4)	d1ryph_(d.153.1.4)	d1rypg_(d.153.1.4)	d1rypb_(d.153.1.4)
d1zuea1	g.9.1.1	43	3	1	1	d1kj6a_(g.9.1.1)	d1ewsa_(g.9.1.1)	d1ijva_(g.9.1.1)	d1kj6a_(g.9.1.1)	d2gyck1(d.41.4.2)	d1oaia_(a.5.2.3)
d2b0aa1	c.8.8.1	187	1	1	1	d1r61a_(c.8.8.1)	d1r61a_(c.8.8.1)	d1pava_(d.68.3.3)	d2b3ya1(c.8.2.1)	d1m32a_(c.67.1.3)	d1i4na_(c.1.2.4)
d2b3ya1	c.8.2.1	260	1	1	1	d1l5ja2(c.8.2.1)	d1l5ja2(c.8.2.1)	d1g6sa_(d.68.2.2)	d1cl1a_(c.67.1.3)	d1o69a_(c.67.1.4)	d2ctza1(c.67.1.3)
d2bwba1	a.5.2.1	45	14	1	1	d2cpwa1(a.5.2.1)	d1wgna_(a.5.2.1)	d1veka_(a.5.2.1)	d1wiva_(a.5.2.1)	d2cpwa1(a.5.2.1)	d2dnaa1(a.5.2.1)
d2c8sa1	a.3.1.1	150	6	1	1	d1ctja_(a.3.1.1)	d1dw0a_(a.3.1.1)	d1ctja_(a.3.1.1)	d1e29a_(a.3.1.1)	d1i8oa_(a.3.1.1)	d1wvec1(a.3.1.1)
d2cpwa1	a.5.2.1	52	14	1	1	d2crna1(a.5.2.1)	d2crna1(a.5.2.1)	d1wgna_(a.5.2.1)	d1veka_(a.5.2.1)	d1wiva_(a.5.2.1)	d2bwba1(a.5.2.1)
d2crna1	a.5.2.1	52	14	1	1	d1veka_(a.5.2.1)	d1veka_(a.5.2.1)	d1wiva_(a.5.2.1)	d1wgna_(a.5.2.1)	d2dnaa1(a.5.2.1)	d2cpwa1(a.5.2.1)
d2cssa1	b.36.1.1	109	10	1	1	d1rgwa_(b.36.1.1)	d1rgwa_(b.36.1.1)	d1tp5a1(b.36.1.1)	d1g9oa_(b.36.1.1)	d1wi4a1(b.36.1.1)	d1va8a1(b.36.1.1)
d2ctza1	c.67.1.3	422	4	1	1	d1cl1a_(c.67.1.3)	d1m32a_(c.67.1.3)	d1cl1a_(c.67.1.3)	d2r5ea1(c.67.1.1)	d1b9ha_(c.67.1.4)	d1o69a_(c.67.1.4)
d2dkla1	a.5.2.1	73	14	1	1	d1wiva_(a.5.2.1)	d1wiva_(a.5.2.1)	d1veka_(a.5.2.1)	d2crna1(a.5.2.1)	d1wgna_(a.5.2.1)	d2cpwa1(a.5.2.1)
d2dnaa1	a.5.2.1	51	14	1	1	d2bwba1(a.5.2.1)	d3e46a1(a.5.2.1)	d1wiva_(a.5.2.1)	d2cpwa1(a.5.2.1)	d2bwba1(a.5.2.1)	d2k0bx1(a.5.2.1)
d2h26a2	d.19.1.1	178	6	1	1	d1lqva_(d.19.1.1)	d1lqva_(d.19.1.1)	d1hyrc2(d.19.1.1)	d1t7va2(d.19.1.1)	d1eoka_(c.1.8.5)	d1ohwa_(c.67.1.4)
d2hoxa1	c.67.1.1	426	4	0	1	d1c7na_(c.67.1.3)	d1v72a1(c.67.1.1)	d1u08a_(c.67.1.1)	d2r5ea1(c.67.1.1)	d1c7na_(c.67.1.3)	d1cl1a_(c.67.1.3)
d2k0bx1	a.5.2.1	53	14	1	1	d2dnaa1(a.5.2.1)	d1wiva_(a.5.2.1)	d2dnaa1(a.5.2.1)	d1wgna_(a.5.2.1)	d2bwba1(a.5.2.1)	d2crna1(a.5.2.1)
d2onka1	c.37.1.12	241	3	1	1	d3dhwc1(c.37.1.12)	d1l2ta_(c.37.1.12)	d1jj7a_(c.37.1.12)	d3dhwc1(c.37.1.12)	d1g6oa_(c.37.1.11)	d1nija1(c.37.1.10)
d2r5ea1	c.67.1.1	419	4	1	1	d1u08a_(c.67.1.1)	d1m6sa_(c.67.1.1)	d1v72a1(c.67.1.1)	d1u08a_(c.67.1.1)	d2hoxa1(c.67.1.1)	d1c7na_(c.67.1.3)
d2tpta3	d.41.3.1	106	1	1	1	d1brwa3(d.41.3.1)	d1brwa3(d.41.3.1)	d2f23a2(d.26.1.2)	d1nj8a2(d.68.5.1)	d1b9ha_(c.67.1.4)	d1wi4a1(b.36.1.1)
d3dhwc1	c.37.1.12	241	3	1	1	d1jj7a_(c.37.1.12)	d1jj7a_(c.37.1.12)	d2onka1(c.37.1.12)	d1l2ta_(c.37.1.12)	d1g6oa_(c.37.1.11)	d1nija1(c.37.1.10)
d3e46a1	a.5.2.1	43	14	1	1	d1wiva_(a.5.2.1)	d1wgna_(a.5.2.1)	d2dnaa1(a.5.2.1)	d2bwba1(a.5.2.1)	d2cpwa1(a.5.2.1)	d1wiva_(a.5.2.1)
**Total Hit**				99	104						
**Total seq**				106	106						
**Success Rate**				93.40%	98.11%						

d1m32a_	c.67.1.3	362	8	1	1	d1m6sa_(c.67.1.1)	d1m6sa_(c.67.1.1)	d1b9ha_(c.67.1.4)	d1o69a_(c.67.1.4)	d1v72a1(c.67.1.1)	d1u08a_(c.67.1.1)
d1m5ya3	d.26.1.1	109	1	0	0	d1jdqa_(d.68.3.3)	d1vaea_(b.36.1.1)	d2c8sa1(a.3.1.1)	d1i8oa_(a.3.1.1)	d1pava_(d.68.3.3)	d1jdqa_(d.68.3.3)
d1m6sa_	c.67.1.1	344	8	1	1	d1ohwa_(c.67.1.4)	d1c7na_(c.67.1.3)	d1o69a_(c.67.1.4)	d1m32a_(c.67.1.3)	d1ohwa_(c.67.1.4)	d1iuga_(c.67.1.3)
d1mn3a_	a.5.2.4	55	18	1	1	d1wgna_(a.5.2.1)	d2bwba1(a.5.2.1)	d3e46a1(a.5.2.1)	d2k0bx1(a.5.2.1)	d1wgna_(a.5.2.1)	d2dnaa1(a.5.2.1)
d1nija1	c.37.1.10	223	5	1	1	d1g6oa_(c.37.1.11)	d1jj7a_(c.37.1.12)	d2onka1(c.37.1.12)	d1g6oa_(c.37.1.11)	d3dhwc1(c.37.1.12)	d1l2ta_(c.37.1.12)
d1o69a_	c.67.1.4	375	10	1	1	d1m6sa_(c.67.1.1)	d1m32a_(c.67.1.3)	d1iuga_(c.67.1.3)	d1cl1a_(c.67.1.3)	d2r5ea1(c.67.1.1)	d2ctza1(c.67.1.3)
d1oaia_	a.5.2.3	60	18	1	1	d1xb2b1(a.5.2.2)	d1xb2b1(a.5.2.2)	d1vdla_(a.5.2.1)	d1wj7a1(a.5.2.1)	d2dnaa1(a.5.2.1)	d1wgna_(a.5.2.1)
d1ohwa_	c.67.1.4	462	10	1	1	d1m6sa_(c.67.1.1)	d1m6sa_(c.67.1.1)	d1m32a_(c.67.1.3)	d1iuga_(c.67.1.3)	d1c7na_(c.67.1.3)	d1v72a1(c.67.1.1)
d1otra_	a.5.2.4	50	18	1	1	d1wj7a1(a.5.2.1)	d1wj7a1(a.5.2.1)	d2bwba1(a.5.2.1)	d2crna1(a.5.2.1)	d1wgna_(a.5.2.1)	d1wiva_(a.5.2.1)
d1pgya_	a.5.2.1	48	5	1	1	d1oaia_(a.5.2.3)	d1v92a_(a.5.2.3)	d1oaia_(a.5.2.3)	d1otra_(a.5.2.4)	d1nj8a2(d.68.5.1)	d1vcoa2(c.37.1.10)
d1q3qa2	c.8.5.2	154	1	1	1	d1kida_(c.8.5.1)	d1kida_(c.8.5.1)	d2isba1(c.8.9.1)	d1wbha1(c.1.10.1)	d1to3a_(c.1.10.1)	d1jo0a_(d.68.4.1)
d1q6ha_	d.26.1.1	211	1	0	0	d1rypg_(d.153.1.4)	d1l5ja2(c.8.2.1)	d1yara1(d.153.1.4)	d1rypg_(d.153.1.4)	d1pava_(d.68.3.3)	d1a9xb1(c.8.3.1)
d1t11a3	d.26.1.1	114	1	1	1	d2f23a2(d.26.1.2)	d2f23a2(d.26.1.2)	d1g9oa_(b.36.1.1)	d1vaea_(b.36.1.1)	d2gyck1(d.41.4.2)	d1jdqa_(d.68.3.3)
d1t16a_	f.4.3.4	428	1	1	1	d1a0tp_(f.4.3.2)	d1a0tp_(f.4.3.2)	d1ejda_(d.68.2.2)	d2hoxa1(c.67.1.1)	d1rf6a_(d.68.2.2)	d1eoka_(c.1.8.5)
d1ttea1	a.5.2.1	56	5	1	1	d1otra_(a.5.2.4)	d1v92a_(a.5.2.3)	d1otra_(a.5.2.4)	d1wvec1(a.3.1.1)	d1msza_(d.68.7.1)	d1jo0a_(d.68.4.1)
d1u08a_	c.67.1.1	383	8	1	1	d1c7na_(c.67.1.3)	d1m32a_(c.67.1.3)	d1b9ha_(c.67.1.4)	d1c7na_(c.67.1.3)	d1cl1a_(c.67.1.3)	d2ctza1(c.67.1.3)
d1u79a_	d.26.1.1	126	1	0	1	d1tp5a1(b.36.1.1)	d2f23a2(d.26.1.2)	d1uewa_(b.36.1.1)	d1q3oa_(b.36.1.1)	d1r6ja_(b.36.1.1)	d1tp5a1(b.36.1.1)
d1v72a1	c.67.1.1	346	8	1	1	d1m32a_(c.67.1.3)	d1m32a_(c.67.1.3)	d1c7na_(c.67.1.3)	d1o69a_(c.67.1.4)	d1iuga_(c.67.1.3)	d1b9ha_(c.67.1.4)
d1v92a_	a.5.2.3	47	18	1	1	d2bwba1(a.5.2.1)	d2bwba1(a.5.2.1)	d1wiva_(a.5.2.1)	d1wj7a1(a.5.2.1)	d1xb2b1(a.5.2.2)	d2dnaa1(a.5.2.1)
d1vcoa2	c.37.1.10	273	5	0	1	d1ps9a1(c.1.4.1)	d3dhwc1(c.37.1.12)	d1m6sa_(c.67.1.1)	d1ps9a1(c.1.4.1)	d1m32a_(c.67.1.3)	d1rf6a_(d.68.2.2)
d1vdla_	a.5.2.1	81	5	1	1	d1xb2b1(a.5.2.2)	d1otra_(a.5.2.4)	d1v92a_(a.5.2.3)	d1oaia_(a.5.2.3)	d1xb2b1(a.5.2.2)	d1whra_(d.68.7.1)
d1vega_	a.5.2.1	84	5	1	1	d1v92a_(a.5.2.3)	d1v92a_(a.5.2.3)	d1whra_(d.68.7.1)	d1hc7a3(d.68.5.1)	d1uewa_(b.36.1.1)	d1jj7a_(c.37.1.12)
d1veka_	a.5.2.1	85	5	1	1	d1xb2b1(a.5.2.2)	d1xb2b1(a.5.2.2)	d1a9xb1(c.8.3.1)	d1uewa_(b.36.1.1)	d1vaea_(b.36.1.1)	d1hc7a3(d.68.5.1)
d1wgna_	a.5.2.1	64	5	1	1	d1xb2b1(a.5.2.2)	d1v92a_(a.5.2.3)	d1oaia_(a.5.2.3)	d1xb2b1(a.5.2.2)	d1otra_(a.5.2.4)	d1wvec1(a.3.1.1)
d1wiva_	a.5.2.1	74	5	1	1	d1xb2b1(a.5.2.2)	d1oaia_(a.5.2.3)	d1otra_(a.5.2.4)	d1xb2b1(a.5.2.2)	d1v92a_(a.5.2.3)	d1wvec1(a.3.1.1)
d1wj7a1	a.5.2.1	92	5	1	1	d1xb2b1(a.5.2.2)	d1otra_(a.5.2.4)	d1xb2b1(a.5.2.2)	d1oaia_(a.5.2.3)	d1v92a_(a.5.2.3)	d1m5ya3(d.26.1.1)
d1xb2b1	a.5.2.2	57	19	1	1	d1wiva_(a.5.2.1)	d1wiva_(a.5.2.1)	d1vdla_(a.5.2.1)	d2bwba1(a.5.2.1)	d2cpwa1(a.5.2.1)	d2dnaa1(a.5.2.1)
d2b3ya1	c.8.2.1	260	1	0	0	d1g6sa_(d.68.2.2)	d1g6sa_(d.68.2.2)	d1cl1a_(c.67.1.3)	d1m32a_(c.67.1.3)	d1o69a_(c.67.1.4)	d2ctza1(c.67.1.3)
d2bwba1	a.5.2.1	45	5	1	1	d1xb2b1(a.5.2.2)	d1xb2b1(a.5.2.2)	d1mn3a_(a.5.2.4)	d1otra_(a.5.2.4)	d1v92a_(a.5.2.3)	d1oaia_(a.5.2.3)
d2cpwa1	a.5.2.1	52	5	1	1	d1xb2b1(a.5.2.2)	d1xb2b1(a.5.2.2)	d1v92a_(a.5.2.3)	d1oaia_(a.5.2.3)	d1otra_(a.5.2.4)	d1wvec1(a.3.1.1)
d2crna1	a.5.2.1	52	5	1	1	d1xb2b1(a.5.2.2)	d1otra_(a.5.2.4)	d1v92a_(a.5.2.3)	d1xb2b1(a.5.2.2)	d1oaia_(a.5.2.3)	d1wvec1(a.3.1.1)
d2ctza1	c.67.1.3	422	8	1	1	d1b9ha_(c.67.1.4)	d2r5ea1(c.67.1.1)	d1u08a_(c.67.1.1)	d1b9ha_(c.67.1.4)	d1m6sa_(c.67.1.1)	d1o69a_(c.67.1.4)
d2dkla1	a.5.2.1	73	5	0	1	d1wvec1(a.3.1.1)	d1xb2b1(a.5.2.2)	d1oaia_(a.5.2.3)	d1jo0a_(d.68.4.1)	d1hdma2(d.19.1.1)	d1wvec1(a.3.1.1)
d2dnaa1	a.5.2.1	51	5	1	1	d1xb2b1(a.5.2.2)	d1otra_(a.5.2.4)	d1mn3a_(a.5.2.4)	d1v92a_(a.5.2.3)	d1oaia_(a.5.2.3)	d1xb2b1(a.5.2.2)
d2f23a2	d.26.1.2	80	7	1	1	d1hxva_(d.26.1.1)	d1t11a3(d.26.1.1)	d1hxva_(d.26.1.1)	d1rgwa_(b.36.1.1)	d1va8a1(b.36.1.1)	d2d0oa1(c.8.6.1)
d2hi6a1	c.8.2.3	133	2	0	1	d1kbla2(c.8.1.1)	d1l5ja2(c.8.2.1)	d1nxja_(c.8.7.1)	d1kbla2(c.8.1.1)	d1a9xb1(c.8.3.1)	d2d0oa1(c.8.6.1)
d2hoxa1	c.67.1.1	426	8	1	1	d1c7na_(c.67.1.3)	d1o69a_(c.67.1.4)	d1b9ha_(c.67.1.4)	d1c7na_(c.67.1.3)	d1m32a_(c.67.1.3)	d1cl1a_(c.67.1.3)
d2k0bx1	a.5.2.1	53	5	1	1	d1oaia_(a.5.2.3)	d1xb2b1(a.5.2.2)	d1otra_(a.5.2.4)	d1v92a_(a.5.2.3)	d1oaia_(a.5.2.3)	d1mn3a_(a.5.2.4)
d2onka1	c.37.1.12	241	4	1	1	d1g6oa_(c.37.1.11)	d1g6oa_(c.37.1.11)	d1nija1(c.37.1.10)	d1g6sa_(d.68.2.2)	d1djqa1(c.1.4.1)	d1ps9a1(c.1.4.1)
d2r5ea1	c.67.1.1	419	8	1	1	d1c7na_(c.67.1.3)	d2ctza1(c.67.1.3)	d1o69a_(c.67.1.4)	d1cl1a_(c.67.1.3)	d1c7na_(c.67.1.3)	d1b9ha_(c.67.1.4)
d3dhwc1	c.37.1.12	241	4	1	1	d1g6oa_(c.37.1.11)	d1g6oa_(c.37.1.11)	d1nija1(c.37.1.10)	d1i4na_(c.1.2.4)	d1djqa1(c.1.4.1)	d1c7na_(c.67.1.3)
d3e46a1	a.5.2.1	43	5	1	1	d1v92a_(a.5.2.3)	d1mn3a_(a.5.2.4)	d1otra_(a.5.2.4)	d1xb2b1(a.5.2.2)	d1v92a_(a.5.2.3)	d1oaia_(a.5.2.3)
**Total Hit**				47	51						
**Total seq**				56	56						
**Success Rate**				83.93%	91.07%						

Fold											
**Name**	**SCOP ID**	**Length**	**Member**	**Rank1 Hit**	**Rank5 Hit**	**Rank1 List**	**Rank5 List**				
d1a9xb1	c.8.3.1	152	11	0	0	d1u08a_(c.67.1.1)	d2ctza1(c.67.1.3)	d1cl1a_(c.67.1.3)	d1u08a_(c.67.1.1)	d1m32a_(c.67.1.3)	d1b9ha_(c.67.1.4)
d1brwa3	d.41.3.1	104	1	0	0	d1wi4a1(b.36.1.1)	d1wi4a1(b.36.1.1)	d2cssa1(b.36.1.1)	d1kbla2(c.8.1.1)	d1nj8a2(d.68.5.1)	d1a9xb1(c.8.3.1)
d1djqa1	c.1.4.1	341	8	1	1	d1rd5a_(c.1.2.4)	d1wbha1(c.1.10.1)	d1rd5a_(c.1.2.4)	d1kkoa1(c.1.11.2)	d1i4na_(c.1.2.4)	d1hl2a_(c.1.10.1)
d1ejda_	d.68.2.2	420	8	0	0	d1m6sa_(c.67.1.1)	d1m6sa_(c.67.1.1)	d1eoka_(c.1.8.5)	d1cl1a_(c.67.1.3)	d1kida_(c.8.5.1)	d1iuga_(c.67.1.3)
d1eoka_	c.1.8.5	283	9	1	1	d1ps9a1(c.1.4.1)	d1djqa1(c.1.4.1)	d1ps9a1(c.1.4.1)	d1t16a_(f.4.3.4)	d2hoxa1(c.67.1.1)	d1g6sa_(d.68.2.2)
d1g6sa_	d.68.2.2	428	8	0	0	d1m32a_(c.67.1.3)	d1m32a_(c.67.1.3)	d1hl2a_(c.1.10.1)	d1iuga_(c.67.1.3)	d1a9xb1(c.8.3.1)	d1cl1a_(c.67.1.3)
d1hc7a3	d.68.5.1	75	9	1	1	d1pava_(d.68.3.3)	d1pava_(d.68.3.3)	d1jdqa_(d.68.3.3)	d1nija1(c.37.1.10)	d1rgwa_(b.36.1.1)	d1ewsa_(g.9.1.1)
d1hl2a_	c.1.10.1	296	7	1	1	d1ps9a1(c.1.4.1)	d1rd5a_(c.1.2.4)	d1ps9a1(c.1.4.1)	d1djqa1(c.1.4.1)	d1m32a_(c.67.1.3)	d1iuga_(c.67.1.3)
d1i4na_	c.1.2.4	252	7	1	1	d1wbha1(c.1.10.1)	d1djqa1(c.1.4.1)	d1to3a_(c.1.10.1)	d1ps9a1(c.1.4.1)	d1wbha1(c.1.10.1)	d1hl2a_(c.1.10.1)
d1jdqa_	d.68.3.3	99	9	1	1	d1msza_(d.68.7.1)	d1msza_(d.68.7.1)	d1r6ja_(b.36.1.1)	d1eq3a_(d.26.1.1)	d1hxva_(d.26.1.1)	d1vaea_(b.36.1.1)
d1jo0a_	d.68.4.1	98	10	0	1	d1byia_(c.37.1.10)	d1msza_(d.68.7.1)	d1kida_(c.8.5.1)	d1hl2a_(c.1.10.1)	d1m5ya3(d.26.1.1)	d1byia_(c.37.1.10)
d1kbla2	c.8.1.1	134	11	1	1	d2hi6a1(c.8.2.3)	d1l5ja2(c.8.2.1)	d2isba1(c.8.9.1)	d1nxja_(c.8.7.1)	d2hi6a1(c.8.2.3)	d1wbha1(c.1.10.1)
d1kida_	c.8.5.1	194	10	0	0	d1ejda_(d.68.2.2)	d1nija1(c.37.1.10)	d1ejda_(d.68.2.2)	d1byia_(c.37.1.10)	d1wbha1(c.1.10.1)	d1hl2a_(c.1.10.1)
d1kkoa1	c.1.11.2	252	9	1	1	d1ps9a1(c.1.4.1)	d1vc4a_(c.1.2.4)	d1djqa1(c.1.4.1)	d1wbha1(c.1.10.1)	d1i4na_(c.1.2.4)	d1ps9a1(c.1.4.1)
d1l5ja2	c.8.2.1	213	9	0	1	d1rd5a_(c.1.2.4)	d1nxja_(c.8.7.1)	d2d0oa1(c.8.6.1)	d1kbla2(c.8.1.1)	d1iuga_(c.67.1.3)	d1djqa1(c.1.4.1)
d1msza_	d.68.7.1	63	9	1	1	d1pava_(d.68.3.3)	d1nj8a2(d.68.5.1)	d2bkya1(d.68.6.1)	d1pava_(d.68.3.3)	d2k0bx1(a.5.2.1)	d2crna1(a.5.2.1)
d1nj8a2	d.68.5.1	63	9	0	1	d1m5ya3(d.26.1.1)	d1whra_(d.68.7.1)	d1msza_(d.68.7.1)	d2bkya1(d.68.6.1)	d1l2ta_(c.37.1.12)	d1m5ya3(d.26.1.1)
d1nxja_	c.8.7.1	157	11	1	1	d1kbla2(c.8.1.1)	d1l5ja2(c.8.2.1)	d1kbla2(c.8.1.1)	d1r61a_(c.8.8.1)	d1pava_(d.68.3.3)	d1byia_(c.37.1.10)
d1pava_	d.68.3.3	79	9	1	1	d1msza_(d.68.7.1)	d1msza_(d.68.7.1)	d1hxva_(d.26.1.1)	d1eq3a_(d.26.1.1)	d1m5ya3(d.26.1.1)	d1r6ja_(b.36.1.1)
d1ps9a1	c.1.4.1	331	8	1	1	d1rd5a_(c.1.2.4)	d1wbha1(c.1.10.1)	d1vc4a_(c.1.2.4)	d1to3a_(c.1.10.1)	d1hl2a_(c.1.10.1)	d1rd5a_(c.1.2.4)
d1q3qa2	c.8.5.2	154	10	1	1	d2isba1(c.8.9.1)	d2isba1(c.8.9.1)	d1jj7a_(c.37.1.12)	d1wbha1(c.1.10.1)	d1to3a_(c.1.10.1)	d1jo0a_(d.68.4.1)
d1r61a_	c.8.8.1	206	10	1	1	d1nxja_(c.8.7.1)	d1nxja_(c.8.7.1)	d2r5ea1(c.67.1.1)	d1nija1(c.37.1.10)	d1ps9a1(c.1.4.1)	d1m32a_(c.67.1.3)
d1rd5a_	c.1.2.4	262	7	1	1	d1wbha1(c.1.10.1)	d1to3a_(c.1.10.1)	d1hl2a_(c.1.10.1)	d1ps9a1(c.1.4.1)	d1wbha1(c.1.10.1)	d1djqa1(c.1.4.1)
d1rf6a_	d.68.2.2	428	8	0	0	d1cl1a_(c.67.1.3)	d1m32a_(c.67.1.3)	d1hl2a_(c.1.10.1)	d2ctza1(c.67.1.3)	d1iuga_(c.67.1.3)	d1cl1a_(c.67.1.3)
d1to3a_	c.1.10.1	292	7	1	1	d1i4na_(c.1.2.4)	d1rd5a_(c.1.2.4)	d1vc4a_(c.1.2.4)	d1djqa1(c.1.4.1)	d1ps9a1(c.1.4.1)	d1i4na_(c.1.2.4)
d1vc4a_	c.1.2.4	255	7	1	1	d1wbha1(c.1.10.1)	d1to3a_(c.1.10.1)	d1djqa1(c.1.4.1)	d1ps9a1(c.1.4.1)	d1wbha1(c.1.10.1)	d1iuga_(c.67.1.3)
d1wbha1	c.1.10.1	214	7	1	1	d1vc4a_(c.1.2.4)	d1rd5a_(c.1.2.4)	d1ps9a1(c.1.4.1)	d1vc4a_(c.1.2.4)	d1djqa1(c.1.4.1)	d1i4na_(c.1.2.4)
d1whra_	d.68.7.1	125	9	0	0	d1l2ta_(c.37.1.12)	d1l2ta_(c.37.1.12)	d1ttea1(a.5.2.1)	d1eoka_(c.1.8.5)	d1q3qa2(c.8.5.2)	d1gu2a_(a.3.1.1)
d2b0aa1	c.8.8.1	187	10	0	1	d1pava_(d.68.3.3)	d1a9xb1(c.8.3.1)	d2b3ya1(c.8.2.1)	d1pava_(d.68.3.3)	d1m32a_(c.67.1.3)	d1i4na_(c.1.2.4)
d2b3ya1	c.8.2.1	260	9	0	0	d1g6sa_(d.68.2.2)	d1g6sa_(d.68.2.2)	d1cl1a_(c.67.1.3)	d1m32a_(c.67.1.3)	d1o69a_(c.67.1.4)	d2ctza1(c.67.1.3)
d2bkya1	d.68.6.1	90	10	0	1	d1kbla2(c.8.1.1)	d1msza_(d.68.7.1)	d1pava_(d.68.3.3)	d1kbla2(c.8.1.1)	d1tp5a1(b.36.1.1)	d1vcoa2(c.37.1.10)
d2d0oa1	c.8.6.1	163	11	1	1	d2hi6a1(c.8.2.3)	d2hi6a1(c.8.2.3)	d1nija1(c.37.1.10)	d1byia_(c.37.1.10)	d1pava_(d.68.3.3)	d2f23a2(d.26.1.2)
d2gyck1	d.41.4.2	132	2	0	0	d2cssa1(b.36.1.1)	d2cssa1(b.36.1.1)	d1tp5a1(b.36.1.1)	d1rgwa_(b.36.1.1)	d1va8a1(b.36.1.1)	d2d0oa1(c.8.6.1)
d2hi6a1	c.8.2.3	133	9	1	1	d1kbla2(c.8.1.1)	d1nxja_(c.8.7.1)	d1kbla2(c.8.1.1)	d1a9xb1(c.8.3.1)	d2d0oa1(c.8.6.1)	d1pava_(d.68.3.3)
d2isba1	c.8.9.1	179	11	0	1	d1m32a_(c.67.1.3)	d1q3qa2(c.8.5.2)	d1m6sa_(c.67.1.1)	d1m32a_(c.67.1.3)	d1ohwa_(c.67.1.4)	d1pava_(d.68.3.3)
d2tpta3	d.41.3.1	106	1	0	0	d1nj8a2(d.68.5.1)	d2f23a2(d.26.1.2)	d1nj8a2(d.68.5.1)	d1b9ha_(c.67.1.4)	d1wi4a1(b.36.1.1)	d1v72a1(c.67.1.1)
**Total Hit**				20	26						
**Total seq**				36	36						
**Success Rate**				55.56%	72.22%						

Prediction results on SCOP_TEST by RFDN-Fold

Family											
**Name**	**SCOP ID**	**Length**	**Member**	**Rank1 Hit**	**Rank5 Hit**	**Rank1 List**	**Rank5 List**				
d1b9ha_	c.67.1.4	385	2	1	1	d1o69a_(c.67.1.4)	d1o69a_(c.67.1.4)	d1m6sa_(c.67.1.1)	d1cl1a_(c.67.1.3)	d2ctza1(c.67.1.3)	d1m32a_(c.67.1.3)
d1brwa3	d.41.3.1	104	1	1	1	d2tpta3(d.41.3.1)	d2tpta3(d.41.3.1)	d2d0oa1(c.8.6.1)	d1wi4a1(b.36.1.1)	d2cssa1(b.36.1.1)	d1tp5a1(b.36.1.1)
d1byia_	c.37.1.10	225	2	1	1	d1nija1(c.37.1.10)	d1vcoa2(c.37.1.10)	d1nija1(c.37.1.10)	d1kkoa1(c.1.11.2)	d1hl2a_(c.1.10.1)	d1jj7a_(c.37.1.12)
d1c7na_	c.67.1.3	395	4	0	0	d2r5ea1(c.67.1.1)	d1v72a1(c.67.1.1)	d1m6sa_(c.67.1.1)	d2r5ea1(c.67.1.1)	d2hoxa1(c.67.1.1)	d1u08a_(c.67.1.1)
d1cl1a_	c.67.1.3	392	4	1	1	d2ctza1(c.67.1.3)	d1m32a_(c.67.1.3)	d1iuga_(c.67.1.3)	d2ctza1(c.67.1.3)	d2r5ea1(c.67.1.1)	d1b9ha_(c.67.1.4)
d1ctja_	a.3.1.1	90	6	1	1	d1wvec1(a.3.1.1)	d2c8sa1(a.3.1.1)	d1gu2a_(a.3.1.1)	d1wvec1(a.3.1.1)	d1i8oa_(a.3.1.1)	d1e29a_(a.3.1.1)
d1djqa1	c.1.4.1	341	1	1	1	d1ps9a1(c.1.4.1)	d1ps9a1(c.1.4.1)	d1wbha1(c.1.10.1)	d1rd5a_(c.1.2.4)	d1kkoa1(c.1.11.2)	d1vc4a_(c.1.2.4)
d1dw0a_	a.3.1.1	113	6	1	1	d1gu2a_(a.3.1.1)	d1gu2a_(a.3.1.1)	d2c8sa1(a.3.1.1)	d1e29a_(a.3.1.1)	d1i8oa_(a.3.1.1)	d1ctja_(a.3.1.1)
d1e29a_	a.3.1.1	136	6	1	1	d1ctja_(a.3.1.1)	d1wvec1(a.3.1.1)	d2c8sa1(a.3.1.1)	d1i8oa_(a.3.1.1)	d1ctja_(a.3.1.1)	d1gu2a_(a.3.1.1)
d1ejda_	d.68.2.2	420	2	1	1	d1g6sa_(d.68.2.2)	d1g6sa_(d.68.2.2)	d1rf6a_(d.68.2.2)	d1m6sa_(c.67.1.1)	d1ps9a1(c.1.4.1)	d1iuga_(c.67.1.3)
d1eq3a_	d.26.1.1	97	6	1	1	d1m5ya3(d.26.1.1)	d1t11a3(d.26.1.1)	d1m5ya3(d.26.1.1)	d1hxva_(d.26.1.1)	d1pava_(d.68.3.3)	d1nj8a2(d.68.5.1)
d1ewsa_	g.9.1.1	33	3	1	1	d1ijva_(g.9.1.1)	d1ijva_(g.9.1.1)	d1kj6a_(g.9.1.1)	d1zuea1(g.9.1.1)	d1hdma2(d.19.1.1)	d1brwa3(d.41.3.1)
d1g3ka_	d.153.1.4	174	7	1	1	d1ryph_(d.153.1.4)	d1ryph_(d.153.1.4)	d1rypg_(d.153.1.4)	d1rype_(d.153.1.4)	d1ryp2_(d.153.1.4)	d1rypc_(d.153.1.4)
d1g6sa_	d.68.2.2	428	2	1	1	d1ejda_(d.68.2.2)	d1ejda_(d.68.2.2)	d1rf6a_(d.68.2.2)	d1m32a_(c.67.1.3)	d1m6sa_(c.67.1.1)	d1iuga_(c.67.1.3)
d1g9oa_	b.36.1.1	92	10	1	1	d1rzxa_(b.36.1.1)	d1rzxa_(b.36.1.1)	d1q3oa_(b.36.1.1)	d1uewa_(b.36.1.1)	d1r6ja_(b.36.1.1)	d1wi4a1(b.36.1.1)
d1gu2a_	a.3.1.1	125	6	1	1	d1dw0a_(a.3.1.1)	d1i8oa_(a.3.1.1)	d1e29a_(a.3.1.1)	d2c8sa1(a.3.1.1)	d1ctja_(a.3.1.1)	d1dw0a_(a.3.1.1)
d1hc7a3	d.68.5.1	75	1	1	1	d1nj8a2(d.68.5.1)	d1nj8a2(d.68.5.1)	d1msza_(d.68.7.1)	d1kj6a_(g.9.1.1)	d1hdmb2(d.19.1.1)	d2bkya1(d.68.6.1)
d1hdma2	d.19.1.1	82	6	1	1	d1hdmb2(d.19.1.1)	d1hyrc2(d.19.1.1)	d1lqva_(d.19.1.1)	d2h26a2(d.19.1.1)	d1hdmb2(d.19.1.1)	d1uvqb2(d.19.1.1)
d1hdmb2	d.19.1.1	86	6	1	1	d1uvqb2(d.19.1.1)	d1hdma2(d.19.1.1)	d1uvqb2(d.19.1.1)	d1lqva_(d.19.1.1)	d2k0bx1(a.5.2.1)	d1i8oa_(a.3.1.1)
d1hl2a_	c.1.10.1	296	2	0	1	d1rd5a_(c.1.2.4)	d1wbha1(c.1.10.1)	d1rd5a_(c.1.2.4)	d1ps9a1(c.1.4.1)	d1i4na_(c.1.2.4)	d1djqa1(c.1.4.1)
d1hxva_	d.26.1.1	86	6	1	1	d1u79a_(d.26.1.1)	d1jvwa_(d.26.1.1)	d1u79a_(d.26.1.1)	d1t11a3(d.26.1.1)	d1q6ha_(d.26.1.1)	d2f23a2(d.26.1.2)
d1hyrc2	d.19.1.1	182	6	1	1	d1t7va2(d.19.1.1)	d1uvqb2(d.19.1.1)	d1t7va2(d.19.1.1)	d1hdma2(d.19.1.1)	d1lqva_(d.19.1.1)	d2h26a2(d.19.1.1)
d1i4na_	c.1.2.4	252	2	1	1	d1vc4a_(c.1.2.4)	d1rd5a_(c.1.2.4)	d1vc4a_(c.1.2.4)	d1to3a_(c.1.10.1)	d1wbha1(c.1.10.1)	d1hl2a_(c.1.10.1)
d1i8oa_	a.3.1.1	115	6	1	1	d1ctja_(a.3.1.1)	d1wvec1(a.3.1.1)	d1e29a_(a.3.1.1)	d1dw0a_(a.3.1.1)	d2c8sa1(a.3.1.1)	d1ctja_(a.3.1.1)
d1ijva_	g.9.1.1	37	3	1	1	d1kj6a_(g.9.1.1)	d1kj6a_(g.9.1.1)	d1zuea1(g.9.1.1)	d1ewsa_(g.9.1.1)	d1jdqa_(d.68.3.3)	d1hc7a3(d.68.5.1)
d1iuga_	c.67.1.3	349	4	1	1	d1m32a_(c.67.1.3)	d1cl1a_(c.67.1.3)	d1m32a_(c.67.1.3)	d1v72a1(c.67.1.1)	d1m6sa_(c.67.1.1)	d1o69a_(c.67.1.4)
d1jdqa_	d.68.3.3	99	1	1	1	d1pava_(d.68.3.3)	d1pava_(d.68.3.3)	d1msza_(d.68.7.1)	d1eq3a_(d.26.1.1)	d1uewa_(b.36.1.1)	d1m5ya3(d.26.1.1)
d1jj7a_	c.37.1.12	252	3	1	1	d1l2ta_(c.37.1.12)	d3dhwc1(c.37.1.12)	d2onka1(c.37.1.12)	d1l2ta_(c.37.1.12)	d1nija1(c.37.1.10)	d1g6oa_(c.37.1.11)
d1jvwa_	d.26.1.1	161	6	1	1	d1u79a_(d.26.1.1)	d1u79a_(d.26.1.1)	d1t11a3(d.26.1.1)	d1q6ha_(d.26.1.1)	d1eq3a_(d.26.1.1)	d1hxva_(d.26.1.1)
d1kj6a_	g.9.1.1	46	3	1	1	d1ijva_(g.9.1.1)	d1zuea1(g.9.1.1)	d1ewsa_(g.9.1.1)	d1ijva_(g.9.1.1)	d1hc7a3(d.68.5.1)	d1jdqa_(d.68.3.3)
d1l2ta_	c.37.1.12	231	3	1	1	d3dhwc1(c.37.1.12)	d3dhwc1(c.37.1.12)	d1jj7a_(c.37.1.12)	d2onka1(c.37.1.12)	d1g6oa_(c.37.1.11)	d1nija1(c.37.1.10)
d1l5ja2	c.8.2.1	213	1	1	1	d2b3ya1(c.8.2.1)	d2b3ya1(c.8.2.1)	d1g6oa_(c.37.1.11)	d2hi6a1(c.8.2.3)	d1nxja_(c.8.7.1)	d1kbla2(c.8.1.1)
d1lqva_	d.19.1.1	173	6	1	1	d2h26a2(d.19.1.1)	d2h26a2(d.19.1.1)	d1t7va2(d.19.1.1)	d1uvqb2(d.19.1.1)	d1hyrc2(d.19.1.1)	d1hdmb2(d.19.1.1)
d1m32a_	c.67.1.3	362	4	1	1	d1iuga_(c.67.1.3)	d1iuga_(c.67.1.3)	d2ctza1(c.67.1.3)	d1m6sa_(c.67.1.1)	d1b9ha_(c.67.1.4)	d1v72a1(c.67.1.1)
d1m5ya3	d.26.1.1	109	6	1	1	d1eq3a_(d.26.1.1)	d1hxva_(d.26.1.1)	d1t11a3(d.26.1.1)	d1eq3a_(d.26.1.1)	d1hdma2(d.19.1.1)	d1jdqa_(d.68.3.3)
d1m6sa_	c.67.1.1	344	4	1	1	d1v72a1(c.67.1.1)	d1u08a_(c.67.1.1)	d1v72a1(c.67.1.1)	d1o69a_(c.67.1.4)	d1m32a_(c.67.1.3)	d1ohwa_(c.67.1.4)
d1mn3a_	a.5.2.4	55	1	1	1	d1otra_(a.5.2.4)	d1otra_(a.5.2.4)	d2bwba1(a.5.2.1)	d3e46a1(a.5.2.1)	d2k0bx1(a.5.2.1)	d1wgna_(a.5.2.1)
d1msza_	d.68.7.1	63	1	1	1	d1whra_(d.68.7.1)	d1whra_(d.68.7.1)	d1nj8a2(d.68.5.1)	d1ttea1(a.5.2.1)	d1pava_(d.68.3.3)	d1jdqa_(d.68.3.3)
d1nija1	c.37.1.10	223	2	1	1	d1byia_(c.37.1.10)	d1byia_(c.37.1.10)	d2onka1(c.37.1.12)	d1g6oa_(c.37.1.11)	d3dhwc1(c.37.1.12)	d1l2ta_(c.37.1.12)
d1nj8a2	d.68.5.1	63	1	1	1	d1hc7a3(d.68.5.1)	d1hc7a3(d.68.5.1)	d1whra_(d.68.7.1)	d1msza_(d.68.7.1)	d1eq3a_(d.26.1.1)	d1m5ya3(d.26.1.1)
d1o69a_	c.67.1.4	375	2	1	1	d1b9ha_(c.67.1.4)	d1b9ha_(c.67.1.4)	d1m32a_(c.67.1.3)	d1cl1a_(c.67.1.3)	d2ctza1(c.67.1.3)	d1m6sa_(c.67.1.1)
d1oaia_	a.5.2.3	60	1	1	1	d1v92a_(a.5.2.3)	d1v92a_(a.5.2.3)	d1xb2b1(a.5.2.2)	d1vdla_(a.5.2.1)	d1wj7a1(a.5.2.1)	d1wgna_(a.5.2.1)
d1ohwa_	c.67.1.4	462	2	0	0	d1m6sa_(c.67.1.1)	d1m6sa_(c.67.1.1)	d1m32a_(c.67.1.3)	d1iuga_(c.67.1.3)	d1c7na_(c.67.1.3)	d1v72a1(c.67.1.1)
d1otra_	a.5.2.4	50	1	0	0	d1wj7a1(a.5.2.1)	d1wj7a1(a.5.2.1)	d2crna1(a.5.2.1)	d1wgna_(a.5.2.1)	d1wiva_(a.5.2.1)	d1veka_(a.5.2.1)
d1pava_	d.68.3.3	79	1	1	1	d1jdqa_(d.68.3.3)	d1jdqa_(d.68.3.3)	d1eq3a_(d.26.1.1)	d1msza_(d.68.7.1)	d1m5ya3(d.26.1.1)	d1r6ja_(b.36.1.1)
d1pgya_	a.5.2.1	48	14	1	1	d1wgna_(a.5.2.1)	d1wiva_(a.5.2.1)	d1veka_(a.5.2.1)	d1wgna_(a.5.2.1)	d3e46a1(a.5.2.1)	d2dnaa1(a.5.2.1)
d1ps9a1	c.1.4.1	331	1	1	1	d1djqa1(c.1.4.1)	d1djqa1(c.1.4.1)	d1vc4a_(c.1.2.4)	d1to3a_(c.1.10.1)	d1hl2a_(c.1.10.1)	d1rd5a_(c.1.2.4)
d1q3oa_	b.36.1.1	105	10	1	1	d1vaea_(b.36.1.1)	d1rzxa_(b.36.1.1)	d1r6ja_(b.36.1.1)	d1vaea_(b.36.1.1)	d2cssa1(b.36.1.1)	d1wi4a1(b.36.1.1)
d1q6ha_	d.26.1.1	211	6	1	1	d1jvwa_(d.26.1.1)	d1t11a3(d.26.1.1)	d1jvwa_(d.26.1.1)	d1u79a_(d.26.1.1)	d1hxva_(d.26.1.1)	d1rypg_(d.153.1.4)
d1r61a_	c.8.8.1	206	1	1	1	d2b0aa1(c.8.8.1)	d2b0aa1(c.8.8.1)	d1a9xb1(c.8.3.1)	d1q3oa_(b.36.1.1)	d1nija1(c.37.1.10)	d1nxja_(c.8.7.1)
d1r6ja_	b.36.1.1	83	10	1	1	d1tp5a1(b.36.1.1)	d1tp5a1(b.36.1.1)	d1rzxa_(b.36.1.1)	d1rgwa_(b.36.1.1)	d2cssa1(b.36.1.1)	d1g9oa_(b.36.1.1)
d1rd5a_	c.1.2.4	262	2	1	1	d1i4na_(c.1.2.4)	d1i4na_(c.1.2.4)	d1vc4a_(c.1.2.4)	d1to3a_(c.1.10.1)	d1ps9a1(c.1.4.1)	d1wbha1(c.1.10.1)
d1rf6a_	d.68.2.2	428	2	1	1	d1ejda_(d.68.2.2)	d1ejda_(d.68.2.2)	d1g6sa_(d.68.2.2)	d1u08a_(c.67.1.1)	d1m32a_(c.67.1.3)	d1iuga_(c.67.1.3)
d1rgwa_	b.36.1.1	86	10	1	1	d1tp5a1(b.36.1.1)	d1tp5a1(b.36.1.1)	d1va8a1(b.36.1.1)	d1g9oa_(b.36.1.1)	d2cssa1(b.36.1.1)	d1vaea_(b.36.1.1)
d1ryp2_	d.153.1.4	234	7	1	1	d1rypc_(d.153.1.4)	d1rypc_(d.153.1.4)	d1ryph_(d.153.1.4)	d1rypg_(d.153.1.4)	d1g3ka_(d.153.1.4)	d1rypb_(d.153.1.4)
d1rypb_	d.153.1.4	251	7	1	1	d1rypc_(d.153.1.4)	d1ryp2_(d.153.1.4)	d1rypc_(d.153.1.4)	d1rype_(d.153.1.4)	d1rypg_(d.153.1.4)	d1g3ka_(d.153.1.4)
d1rypc_	d.153.1.4	245	7	1	1	d1yara1(d.153.1.4)	d1yara1(d.153.1.4)	d1ryp2_(d.153.1.4)	d1rypg_(d.153.1.4)	d1rypb_(d.153.1.4)	d1ryph_(d.153.1.4)
d1rype_	d.153.1.4	243	7	1	1	d1rypg_(d.153.1.4)	d1rypg_(d.153.1.4)	d1g3ka_(d.153.1.4)	d1ryp2_(d.153.1.4)	d1yara1(d.153.1.4)	d1ryph_(d.153.1.4)
d1rypg_	d.153.1.4	245	7	1	1	d1yara1(d.153.1.4)	d1yara1(d.153.1.4)	d1rype_(d.153.1.4)	d1rypc_(d.153.1.4)	d1ryp2_(d.153.1.4)	d1g3ka_(d.153.1.4)
d1ryph_	d.153.1.4	206	7	1	1	d1rypb_(d.153.1.4)	d1ryp2_(d.153.1.4)	d1yara1(d.153.1.4)	d1rype_(d.153.1.4)	d1g3ka_(d.153.1.4)	d1rypc_(d.153.1.4)

Superfamily											
**Name**	**SCOP ID**	**Length**	**Member**	**Rank1 Hit**	**Rank5 Hit**	**Rank1 List**	**Rank5 List**				
d1a0tp_	f.4.3.2	414	1	1	1	d1t16a_(f.4.3.4)	d1t16a_(f.4.3.4)	d1a9xb1(c.8.3.1)	d1eoka_(c.1.8.5)	d2b0aa1(c.8.8.1)	d1nxja_(c.8.7.1)
d1b9ha_	c.67.1.4	385	10	1	1	d2ctza1(c.67.1.3)	d1u08a_(c.67.1.1)	d1m6sa_(c.67.1.1)	d1cl1a_(c.67.1.3)	d2ctza1(c.67.1.3)	d1m32a_(c.67.1.3)
d1byia_	c.37.1.10	225	5	0	1	d1kkoa1(c.1.11.2)	d1jj7a_(c.37.1.12)	d1kkoa1(c.1.11.2)	d2d0oa1(c.8.6.1)	d1hl2a_(c.1.10.1)	d1u08a_(c.67.1.1)
d1c7na_	c.67.1.3	395	8	1	1	d2r5ea1(c.67.1.1)	d1v72a1(c.67.1.1)	d1m6sa_(c.67.1.1)	d2r5ea1(c.67.1.1)	d2hoxa1(c.67.1.1)	d1u08a_(c.67.1.1)
d1cl1a_	c.67.1.3	392	8	1	1	d1b9ha_(c.67.1.4)	d1u08a_(c.67.1.1)	d2r5ea1(c.67.1.1)	d1m6sa_(c.67.1.1)	d1b9ha_(c.67.1.4)	d1o69a_(c.67.1.4)
d1eq3a_	d.26.1.1	97	1	0	0	d1nj8a2(d.68.5.1)	d1jdqa_(d.68.3.3)	d1jo0a_(d.68.4.1)	d1pava_(d.68.3.3)	d1hdma2(d.19.1.1)	d1nj8a2(d.68.5.1)
d1g6oa_	c.37.1.11	324	7	1	1	d1jj7a_(c.37.1.12)	d2onka1(c.37.1.12)	d1l2ta_(c.37.1.12)	d1jj7a_(c.37.1.12)	d3dhwc1(c.37.1.12)	d1nija1(c.37.1.10)
d1hxva_	d.26.1.1	86	1	1	1	d2f23a2(d.26.1.2)	d2f23a2(d.26.1.2)	d1q3oa_(b.36.1.1)	d1jdqa_(d.68.3.3)	d1g9oa_(b.36.1.1)	d1rzxa_(b.36.1.1)
d1iuga_	c.67.1.3	349	8	1	1	d1v72a1(c.67.1.1)	d2r5ea1(c.67.1.1)	d1v72a1(c.67.1.1)	d1m6sa_(c.67.1.1)	d1o69a_(c.67.1.4)	d1b9ha_(c.67.1.4)
d1jj7a_	c.37.1.12	252	4	1	1	d1g6oa_(c.37.1.11)	d1nija1(c.37.1.10)	d1g6oa_(c.37.1.11)	d1byia_(c.37.1.10)	d1ps9a1(c.1.4.1)	d1djqa1(c.1.4.1)
d1jvwa_	d.26.1.1	161	1	0	0	d1va8a1(b.36.1.1)	d1pava_(d.68.3.3)	d1jdqa_(d.68.3.3)	d1va8a1(b.36.1.1)	d1q3oa_(b.36.1.1)	d1l5ja2(c.8.2.1)
d1kida_	c.8.5.1	194	1	1	1	d1q3qa2(c.8.5.2)	d1q3qa2(c.8.5.2)	d1nija1(c.37.1.10)	d2d0oa1(c.8.6.1)	d1i4na_(c.1.2.4)	d1wbha1(c.1.10.1)
d1l2ta_	c.37.1.12	231	4	1	1	d1g6oa_(c.37.1.11)	d1g6oa_(c.37.1.11)	d1nija1(c.37.1.10)	d1vcoa2(c.37.1.10)	d1vc4a_(c.1.2.4)	d1kida_(c.8.5.1)
d1l5ja2	c.8.2.1	213	1	1	1	d2hi6a1(c.8.2.3)	d2hi6a1(c.8.2.3)	d1g6oa_(c.37.1.11)	d1nxja_(c.8.7.1)	d1kbla2(c.8.1.1)	d1rd5a_(c.1.2.4)
d1m32a_	c.67.1.3	362	8	1	1	d1m6sa_(c.67.1.1)	d1m6sa_(c.67.1.1)	d1b9ha_(c.67.1.4)	d1o69a_(c.67.1.4)	d1v72a1(c.67.1.1)	d1u08a_(c.67.1.1)
d1m5ya3	d.26.1.1	109	1	0	0	d1jdqa_(d.68.3.3)	d1hdma2(d.19.1.1)	d1uvqb2(d.19.1.1)	d1pava_(d.68.3.3)	d1jdqa_(d.68.3.3)	d1nj8a2(d.68.5.1)
d1m6sa_	c.67.1.1	344	8	1	1	d1ohwa_(c.67.1.4)	d1c7na_(c.67.1.3)	d1o69a_(c.67.1.4)	d1m32a_(c.67.1.3)	d1ohwa_(c.67.1.4)	d1iuga_(c.67.1.3)
d1mn3a_	a.5.2.4	55	18	1	1	d1wgna_(a.5.2.1)	d2bwba1(a.5.2.1)	d3e46a1(a.5.2.1)	d2k0bx1(a.5.2.1)	d1wgna_(a.5.2.1)	d2dnaa1(a.5.2.1)
d1nija1	c.37.1.10	223	5	1	1	d2onka1(c.37.1.12)	d2onka1(c.37.1.12)	d1g6oa_(c.37.1.11)	d3dhwc1(c.37.1.12)	d1l2ta_(c.37.1.12)	d2d0oa1(c.8.6.1)
d1o69a_	c.67.1.4	375	10	1	1	d1m6sa_(c.67.1.1)	d1m32a_(c.67.1.3)	d1iuga_(c.67.1.3)	d1cl1a_(c.67.1.3)	d2ctza1(c.67.1.3)	d1m6sa_(c.67.1.1)
d1oaia_	a.5.2.3	60	18	1	1	d1xb2b1(a.5.2.2)	d1xb2b1(a.5.2.2)	d1vdla_(a.5.2.1)	d1wj7a1(a.5.2.1)	d2dnaa1(a.5.2.1)	d1wgna_(a.5.2.1)
d1ohwa_	c.67.1.4	462	10	1	1	d1m6sa_(c.67.1.1)	d1m6sa_(c.67.1.1)	d1m32a_(c.67.1.3)	d1iuga_(c.67.1.3)	d1c7na_(c.67.1.3)	d1v72a1(c.67.1.1)
d1otra_	a.5.2.4	50	18	1	1	d1wj7a1(a.5.2.1)	d1wj7a1(a.5.2.1)	d2crna1(a.5.2.1)	d1wgna_(a.5.2.1)	d1wiva_(a.5.2.1)	d1veka_(a.5.2.1)
d1pgya_	a.5.2.1	48	5	1	1	d1oaia_(a.5.2.3)	d1v92a_(a.5.2.3)	d1oaia_(a.5.2.3)	d1mn3a_(a.5.2.4)	d1xb2b1(a.5.2.2)	d1otra_(a.5.2.4)
d1q3qa2	c.8.5.2	154	1	1	1	d1kida_(c.8.5.1)	d1kida_(c.8.5.1)	d2isba1(c.8.9.1)	d1a9xb1(c.8.3.1)	d1kbla2(c.8.1.1)	d1wbha1(c.1.10.1)
d1q6ha_	d.26.1.1	211	1	0	0	d1rypg_(d.153.1.4)	d1yara1(d.153.1.4)	d1rypb_(d.153.1.4)	d1rypg_(d.153.1.4)	d1rype_(d.153.1.4)	d1b9ha_(c.67.1.4)
d1t11a3	d.26.1.1	114	1	1	1	d2f23a2(d.26.1.2)	d2f23a2(d.26.1.2)	d2cssa1(b.36.1.1)	d1g9oa_(b.36.1.1)	d1vaea_(b.36.1.1)	d1jdqa_(d.68.3.3)
d1t16a_	f.4.3.4	428	1	1	1	d1a0tp_(f.4.3.2)	d1a0tp_(f.4.3.2)	d1ejda_(d.68.2.2)	d1g6sa_(d.68.2.2)	d1rf6a_(d.68.2.2)	d1eoka_(c.1.8.5)
d1ttea1	a.5.2.1	56	5	0	1	d1msza_(d.68.7.1)	d1xb2b1(a.5.2.2)	d1mn3a_(a.5.2.4)	d1otra_(a.5.2.4)	d1msza_(d.68.7.1)	d1jo0a_(d.68.4.1)
d1u08a_	c.67.1.1	383	8	1	1	d1c7na_(c.67.1.3)	d1m32a_(c.67.1.3)	d1b9ha_(c.67.1.4)	d1c7na_(c.67.1.3)	d1cl1a_(c.67.1.3)	d2ctza1(c.67.1.3)
d1u79a_	d.26.1.1	126	1	0	1	d1uewa_(b.36.1.1)	d2f23a2(d.26.1.2)	d1g9oa_(b.36.1.1)	d1uewa_(b.36.1.1)	d1q3oa_(b.36.1.1)	d1tp5a1(b.36.1.1)
d1v72a1	c.67.1.1	346	8	1	1	d1m32a_(c.67.1.3)	d1m32a_(c.67.1.3)	d1c7na_(c.67.1.3)	d1o69a_(c.67.1.4)	d1iuga_(c.67.1.3)	d1b9ha_(c.67.1.4)
d1v92a_	a.5.2.3	47	18	1	1	d2bwba1(a.5.2.1)	d2bwba1(a.5.2.1)	d1wiva_(a.5.2.1)	d1wj7a1(a.5.2.1)	d1xb2b1(a.5.2.2)	d2dnaa1(a.5.2.1)
d1vcoa2	c.37.1.10	273	5	0	1	d1ps9a1(c.1.4.1)	d3dhwc1(c.37.1.12)	d1g6oa_(c.37.1.11)	d1rd5a_(c.1.2.4)	d1v72a1(c.67.1.1)	d1ps9a1(c.1.4.1)
d1vdla_	a.5.2.1	81	5	1	1	d1xb2b1(a.5.2.2)	d1otra_(a.5.2.4)	d1v92a_(a.5.2.3)	d1oaia_(a.5.2.3)	d1xb2b1(a.5.2.2)	d1mn3a_(a.5.2.4)
d1vega_	a.5.2.1	84	5	1	1	d1v92a_(a.5.2.3)	d1v92a_(a.5.2.3)	d1oaia_(a.5.2.3)	d1jo0a_(d.68.4.1)	d1dw0a_(a.3.1.1)	d1wvec1(a.3.1.1)
d1veka_	a.5.2.1	85	5	1	1	d1xb2b1(a.5.2.2)	d1mn3a_(a.5.2.4)	d1xb2b1(a.5.2.2)	d1otra_(a.5.2.4)	d1v92a_(a.5.2.3)	d1oaia_(a.5.2.3)
d1wgna_	a.5.2.1	64	5	1	1	d1xb2b1(a.5.2.2)	d1v92a_(a.5.2.3)	d1oaia_(a.5.2.3)	d1xb2b1(a.5.2.2)	d1otra_(a.5.2.4)	d1wvec1(a.3.1.1)
d1wiva_	a.5.2.1	74	5	1	1	d1xb2b1(a.5.2.2)	d1oaia_(a.5.2.3)	d1otra_(a.5.2.4)	d1xb2b1(a.5.2.2)	d1v92a_(a.5.2.3)	d1wvec1(a.3.1.1)
d1wj7a1	a.5.2.1	92	5	1	1	d1xb2b1(a.5.2.2)	d1otra_(a.5.2.4)	d1xb2b1(a.5.2.2)	d1oaia_(a.5.2.3)	d1v92a_(a.5.2.3)	d1wvec1(a.3.1.1)
d1xb2b1	a.5.2.2	57	19	1	1	d1wiva_(a.5.2.1)	d1wiva_(a.5.2.1)	d1vdla_(a.5.2.1)	d2bwba1(a.5.2.1)	d2cpwa1(a.5.2.1)	d2dnaa1(a.5.2.1)
d2b3ya1	c.8.2.1	260	1	0	0	d1i4na_(c.1.2.4)	d1djqa1(c.1.4.1)	d1eoka_(c.1.8.5)	d1vc4a_(c.1.2.4)	d1kida_(c.8.5.1)	d1i4na_(c.1.2.4)
d2bwba1	a.5.2.1	45	5	1	1	d1xb2b1(a.5.2.2)	d1xb2b1(a.5.2.2)	d1mn3a_(a.5.2.4)	d1otra_(a.5.2.4)	d1v92a_(a.5.2.3)	d1oaia_(a.5.2.3)
d2cpwa1	a.5.2.1	52	5	1	1	d1xb2b1(a.5.2.2)	d1xb2b1(a.5.2.2)	d1v92a_(a.5.2.3)	d1oaia_(a.5.2.3)	d1otra_(a.5.2.4)	d1wvec1(a.3.1.1)
d2crna1	a.5.2.1	52	5	1	1	d1xb2b1(a.5.2.2)	d1otra_(a.5.2.4)	d1v92a_(a.5.2.3)	d1xb2b1(a.5.2.2)	d1mn3a_(a.5.2.4)	d1oaia_(a.5.2.3)
d2ctza1	c.67.1.3	422	8	1	1	d1b9ha_(c.67.1.4)	d2r5ea1(c.67.1.1)	d1u08a_(c.67.1.1)	d1b9ha_(c.67.1.4)	d1m6sa_(c.67.1.1)	d1o69a_(c.67.1.4)
d2dkla1	a.5.2.1	73	5	1	1	d1otra_(a.5.2.4)	d1v92a_(a.5.2.3)	d1otra_(a.5.2.4)	d1xb2b1(a.5.2.2)	d1oaia_(a.5.2.3)	d1msza_(d.68.7.1)
d2dnaa1	a.5.2.1	51	5	1	1	d1xb2b1(a.5.2.2)	d1otra_(a.5.2.4)	d1mn3a_(a.5.2.4)	d1v92a_(a.5.2.3)	d1oaia_(a.5.2.3)	d1xb2b1(a.5.2.2)
d2f23a2	d.26.1.2	80	7	1	1	d1hxva_(d.26.1.1)	d1t11a3(d.26.1.1)	d1hxva_(d.26.1.1)	d1g9oa_(b.36.1.1)	d1r6ja_(b.36.1.1)	d1va8a1(b.36.1.1)
d2hi6a1	c.8.2.3	133	2	0	1	d1kbla2(c.8.1.1)	d1l5ja2(c.8.2.1)	d1nxja_(c.8.7.1)	d1kbla2(c.8.1.1)	d1a9xb1(c.8.3.1)	d2d0oa1(c.8.6.1)
d2hoxa1	c.67.1.1	426	8	1	1	d1c7na_(c.67.1.3)	d1b9ha_(c.67.1.4)	d1c7na_(c.67.1.3)	d1m32a_(c.67.1.3)	d2ctza1(c.67.1.3)	d1cl1a_(c.67.1.3)
d2k0bx1	a.5.2.1	53	5	1	1	d1oaia_(a.5.2.3)	d1xb2b1(a.5.2.2)	d1otra_(a.5.2.4)	d1v92a_(a.5.2.3)	d1oaia_(a.5.2.3)	d1mn3a_(a.5.2.4)
d2onka1	c.37.1.12	241	4	1	1	d1g6oa_(c.37.1.11)	d1g6oa_(c.37.1.11)	d1nija1(c.37.1.10)	d1byia_(c.37.1.10)	d1g6sa_(d.68.2.2)	d1ps9a1(c.1.4.1)
d2r5ea1	c.67.1.1	419	8	1	1	d1c7na_(c.67.1.3)	d2ctza1(c.67.1.3)	d1cl1a_(c.67.1.3)	d1c7na_(c.67.1.3)	d1b9ha_(c.67.1.4)	d1m32a_(c.67.1.3)
d3dhwc1	c.37.1.12	241	4	1	1	d1g6oa_(c.37.1.11)	d1g6oa_(c.37.1.11)	d1nija1(c.37.1.10)	d1vcoa2(c.37.1.10)	d1djqa1(c.1.4.1)	d1to3a_(c.1.10.1)
d3e46a1	a.5.2.1	43	5	1	1	d1v92a_(a.5.2.3)	d1mn3a_(a.5.2.4)	d1otra_(a.5.2.4)	d1xb2b1(a.5.2.2)	d1v92a_(a.5.2.3)	d1oaia_(a.5.2.3)
**Total Hit**				46	51						
**Total seq**				56	56						
**Success Rate**				82.14%	91.07%						

d1rzxa_	b.36.1.1	99	10	1	1	d1tp5a1(b.36.1.1)	d1q3oa_(b.36.1.1)	d1tp5a1(b.36.1.1)	d1g9oa_(b.36.1.1)	d1wi4a1(b.36.1.1)	d1uewa_(b.36.1.1)
d1t11a3	d.26.1.1	114	6	1	1	d1hxva_(d.26.1.1)	d1q6ha_(d.26.1.1)	d1hxva_(d.26.1.1)	d1u79a_(d.26.1.1)	d1jvwa_(d.26.1.1)	d2f23a2(d.26.1.2)
d1t7va2	d.19.1.1	180	6	1	1	d1hyrc2(d.19.1.1)	d2h26a2(d.19.1.1)	d1uvqb2(d.19.1.1)	d1hdma2(d.19.1.1)	d1hyrc2(d.19.1.1)	d1lqva_(d.19.1.1)
d1to3a_	c.1.10.1	292	2	0	1	d1rd5a_(c.1.2.4)	d1wbha1(c.1.10.1)	d1rd5a_(c.1.2.4)	d1vc4a_(c.1.2.4)	d1ps9a1(c.1.4.1)	d1i4na_(c.1.2.4)
d1tp5a1	b.36.1.1	103	10	1	1	d1q3oa_(b.36.1.1)	d1q3oa_(b.36.1.1)	d1rgwa_(b.36.1.1)	d1va8a1(b.36.1.1)	d1g9oa_(b.36.1.1)	d1r6ja_(b.36.1.1)
d1ttea1	a.5.2.1	56	14	1	1	d1wiva_(a.5.2.1)	d1veka_(a.5.2.1)	d1wgna_(a.5.2.1)	d1wiva_(a.5.2.1)	d2cpwa1(a.5.2.1)	d2crna1(a.5.2.1)
d1u08a_	c.67.1.1	383	4	1	1	d2r5ea1(c.67.1.1)	d2r5ea1(c.67.1.1)	d1m6sa_(c.67.1.1)	d2hoxa1(c.67.1.1)	d1b9ha_(c.67.1.4)	d1c7na_(c.67.1.3)
d1u79a_	d.26.1.1	126	6	1	1	d1jvwa_(d.26.1.1)	d1jvwa_(d.26.1.1)	d1q6ha_(d.26.1.1)	d1hxva_(d.26.1.1)	d1t11a3(d.26.1.1)	d1uewa_(b.36.1.1)
d1uewa_	b.36.1.1	115	10	1	1	d2cssa1(b.36.1.1)	d1g9oa_(b.36.1.1)	d1rgwa_(b.36.1.1)	d2cssa1(b.36.1.1)	d1vaea_(b.36.1.1)	d1wi4a1(b.36.1.1)
d1uvqb2	d.19.1.1	93	6	1	1	d1lqva_(d.19.1.1)	d1t7va2(d.19.1.1)	d1hdmb2(d.19.1.1)	d1lqva_(d.19.1.1)	d1hyrc2(d.19.1.1)	d1hdma2(d.19.1.1)
d1v72a1	c.67.1.1	346	4	1	1	d1m6sa_(c.67.1.1)	d1m6sa_(c.67.1.1)	d1u08a_(c.67.1.1)	d1m32a_(c.67.1.3)	d1iuga_(c.67.1.3)	d1b9ha_(c.67.1.4)
d1v92a_	a.5.2.3	47	1	1	1	d1oaia_(a.5.2.3)	d1oaia_(a.5.2.3)	d2bwba1(a.5.2.1)	d1wiva_(a.5.2.1)	d1xb2b1(a.5.2.2)	d2dnaa1(a.5.2.1)
d1va8a1	b.36.1.1	101	10	1	1	d2cssa1(b.36.1.1)	d2cssa1(b.36.1.1)	d1r6ja_(b.36.1.1)	d1rgwa_(b.36.1.1)	d1wi4a1(b.36.1.1)	d1vaea_(b.36.1.1)
d1vaea_	b.36.1.1	112	10	1	1	d1tp5a1(b.36.1.1)	d1uewa_(b.36.1.1)	d1tp5a1(b.36.1.1)	d1rgwa_(b.36.1.1)	d1g9oa_(b.36.1.1)	d1q3oa_(b.36.1.1)
d1vc4a_	c.1.2.4	255	2	1	1	d1i4na_(c.1.2.4)	d1i4na_(c.1.2.4)	d1rd5a_(c.1.2.4)	d1to3a_(c.1.10.1)	d1ps9a1(c.1.4.1)	d1wbha1(c.1.10.1)
d1vcoa2	c.37.1.10	273	2	1	1	d1byia_(c.37.1.10)	d1byia_(c.37.1.10)	d1rd5a_(c.1.2.4)	d1v72a1(c.67.1.1)	d3dhwc1(c.37.1.12)	d1ps9a1(c.1.4.1)
d1vdla_	a.5.2.1	81	14	1	1	d1wj7a1(a.5.2.1)	d1wgna_(a.5.2.1)	d1wj7a1(a.5.2.1)	d2bwba1(a.5.2.1)	d2dnaa1(a.5.2.1)	d1xb2b1(a.5.2.2)
d1vega_	a.5.2.1	84	14	1	1	d1wiva_(a.5.2.1)	d2cpwa1(a.5.2.1)	d1wiva_(a.5.2.1)	d1veka_(a.5.2.1)	d1wgna_(a.5.2.1)	d2bwba1(a.5.2.1)
d1veka_	a.5.2.1	85	14	1	1	d2crna1(a.5.2.1)	d1wiva_(a.5.2.1)	d2cpwa1(a.5.2.1)	d2crna1(a.5.2.1)	d1wgna_(a.5.2.1)	d2bwba1(a.5.2.1)
d1wbha1	c.1.10.1	214	2	0	1	d1vc4a_(c.1.2.4)	d1hl2a_(c.1.10.1)	d1rd5a_(c.1.2.4)	d1ps9a1(c.1.4.1)	d1vc4a_(c.1.2.4)	d1i4na_(c.1.2.4)
d1wgna_	a.5.2.1	64	14	1	1	d1veka_(a.5.2.1)	d3e46a1(a.5.2.1)	d2cpwa1(a.5.2.1)	d1wiva_(a.5.2.1)	d2crna1(a.5.2.1)	d1veka_(a.5.2.1)
d1whra_	d.68.7.1	125	1	1	1	d1msza_(d.68.7.1)	d1msza_(d.68.7.1)	d1e29a_(a.3.1.1)	d1kida_(c.8.5.1)	d1jo0a_(d.68.4.1)	d1i8oa_(a.3.1.1)
d1wi4a1	b.36.1.1	97	10	1	1	d1tp5a1(b.36.1.1)	d1g9oa_(b.36.1.1)	d1rgwa_(b.36.1.1)	d1tp5a1(b.36.1.1)	d1va8a1(b.36.1.1)	d1rzxa_(b.36.1.1)
d1wiva_	a.5.2.1	74	14	1	1	d1wgna_(a.5.2.1)	d1wgna_(a.5.2.1)	d1veka_(a.5.2.1)	d2crna1(a.5.2.1)	d2cpwa1(a.5.2.1)	d2bwba1(a.5.2.1)
d1wj7a1	a.5.2.1	92	14	1	1	d1wiva_(a.5.2.1)	d1vdla_(a.5.2.1)	d1wiva_(a.5.2.1)	d2dnaa1(a.5.2.1)	d1veka_(a.5.2.1)	d2bwba1(a.5.2.1)
d1wvec1	a.3.1.1	75	6	1	1	d2c8sa1(a.3.1.1)	d1dw0a_(a.3.1.1)	d1e29a_(a.3.1.1)	d1i8oa_(a.3.1.1)	d1ctja_(a.3.1.1)	d2c8sa1(a.3.1.1)
d1yara1	d.153.1.4	222	7	1	1	d1rype_(d.153.1.4)	d1g3ka_(d.153.1.4)	d1rype_(d.153.1.4)	d1ryph_(d.153.1.4)	d1rypg_(d.153.1.4)	d1rypb_(d.153.1.4)
d1zuea1	g.9.1.1	43	3	1	1	d1kj6a_(g.9.1.1)	d1ewsa_(g.9.1.1)	d1ijva_(g.9.1.1)	d1kj6a_(g.9.1.1)	d1hc7a3(d.68.5.1)	d1hdmb2(d.19.1.1)
d2b0aa1	c.8.8.1	187	1	1	1	d1r61a_(c.8.8.1)	d1r61a_(c.8.8.1)	d1a9xb1(c.8.3.1)	d1pava_(d.68.3.3)	d2b3ya1(c.8.2.1)	d1u08a_(c.67.1.1)
d2b3ya1	c.8.2.1	260	1	1	1	d1l5ja2(c.8.2.1)	d1l5ja2(c.8.2.1)	d1djqa1(c.1.4.1)	d1eoka_(c.1.8.5)	d1kida_(c.8.5.1)	d1i4na_(c.1.2.4)
d2bwba1	a.5.2.1	45	14	1	1	d2cpwa1(a.5.2.1)	d1wgna_(a.5.2.1)	d1veka_(a.5.2.1)	d1wiva_(a.5.2.1)	d2cpwa1(a.5.2.1)	d2dnaa1(a.5.2.1)
d2c8sa1	a.3.1.1	150	6	1	1	d1ctja_(a.3.1.1)	d1dw0a_(a.3.1.1)	d1ctja_(a.3.1.1)	d1e29a_(a.3.1.1)	d1i8oa_(a.3.1.1)	d1wvec1(a.3.1.1)
d2cpwa1	a.5.2.1	52	14	1	1	d2crna1(a.5.2.1)	d2crna1(a.5.2.1)	d1wgna_(a.5.2.1)	d1veka_(a.5.2.1)	d1wiva_(a.5.2.1)	d2bwba1(a.5.2.1)
d2crna1	a.5.2.1	52	14	1	1	d1veka_(a.5.2.1)	d1veka_(a.5.2.1)	d1wiva_(a.5.2.1)	d1wgna_(a.5.2.1)	d2dnaa1(a.5.2.1)	d2cpwa1(a.5.2.1)
d2cssa1	b.36.1.1	109	10	1	1	d1rgwa_(b.36.1.1)	d1rgwa_(b.36.1.1)	d1tp5a1(b.36.1.1)	d1g9oa_(b.36.1.1)	d1wi4a1(b.36.1.1)	d1va8a1(b.36.1.1)
d2ctza1	c.67.1.3	422	4	1	1	d1cl1a_(c.67.1.3)	d1m32a_(c.67.1.3)	d1cl1a_(c.67.1.3)	d2r5ea1(c.67.1.1)	d1b9ha_(c.67.1.4)	d1o69a_(c.67.1.4)
d2dkla1	a.5.2.1	73	14	1	1	d1wiva_(a.5.2.1)	d1wiva_(a.5.2.1)	d1veka_(a.5.2.1)	d2crna1(a.5.2.1)	d1wgna_(a.5.2.1)	d2cpwa1(a.5.2.1)
d2dnaa1	a.5.2.1	51	14	1	1	d2bwba1(a.5.2.1)	d3e46a1(a.5.2.1)	d1wiva_(a.5.2.1)	d2cpwa1(a.5.2.1)	d2bwba1(a.5.2.1)	d2k0bx1(a.5.2.1)
d2h26a2	d.19.1.1	178	6	1	1	d1lqva_(d.19.1.1)	d1lqva_(d.19.1.1)	d1hyrc2(d.19.1.1)	d1t7va2(d.19.1.1)	d1hdma2(d.19.1.1)	d1uvqb2(d.19.1.1)
d2hoxa1	c.67.1.1	426	4	0	1	d1c7na_(c.67.1.3)	d1v72a1(c.67.1.1)	d1u08a_(c.67.1.1)	d2r5ea1(c.67.1.1)	d1c7na_(c.67.1.3)	d1cl1a_(c.67.1.3)
d2k0bx1	a.5.2.1	53	14	1	1	d2dnaa1(a.5.2.1)	d1wiva_(a.5.2.1)	d2dnaa1(a.5.2.1)	d1wgna_(a.5.2.1)	d2bwba1(a.5.2.1)	d2crna1(a.5.2.1)
d2onka1	c.37.1.12	241	3	1	1	d3dhwc1(c.37.1.12)	d1l2ta_(c.37.1.12)	d1jj7a_(c.37.1.12)	d3dhwc1(c.37.1.12)	d1g6oa_(c.37.1.11)	d1nija1(c.37.1.10)
d2r5ea1	c.67.1.1	419	4	1	1	d1u08a_(c.67.1.1)	d1m6sa_(c.67.1.1)	d1u08a_(c.67.1.1)	d2hoxa1(c.67.1.1)	d1c7na_(c.67.1.3)	d1b9ha_(c.67.1.4)
d2tpta3	d.41.3.1	106	1	1	1	d1brwa3(d.41.3.1)	d1brwa3(d.41.3.1)	d1tp5a1(b.36.1.1)	d2gyck1(d.41.4.2)	d1nj8a2(d.68.5.1)	d1wi4a1(b.36.1.1)
d3dhwc1	c.37.1.12	241	3	1	1	d1jj7a_(c.37.1.12)	d1jj7a_(c.37.1.12)	d2onka1(c.37.1.12)	d1l2ta_(c.37.1.12)	d1g6oa_(c.37.1.11)	d1nija1(c.37.1.10)
d3e46a1	a.5.2.1	43	14	1	1	d1wiva_(a.5.2.1)	d1wgna_(a.5.2.1)	d2dnaa1(a.5.2.1)	d2bwba1(a.5.2.1)	d2cpwa1(a.5.2.1)	d1wiva_(a.5.2.1)
**Total Hit**				**99**	**103**						
**Total seq**				**106**	**106**						
**Success Rate**				**93.40%**	**97.17%**						

Fold											
**Name**	**SCOP ID**	**Length**	**Member**	**Rank1 Hit**	**Rank5 Hit**	**Rank1 List**	**Rank5 List**				
d1a9xb1	c.8.3.1	152	11	0	1	d1u08a_(c.67.1.1)	d2b0aa1(c.8.8.1)	d2ctza1(c.67.1.3)	d1u08a_(c.67.1.1)	d1i4na_(c.1.2.4)	d1r6ja_(b.36.1.1)
d1brwa3	d.41.3.1	104	1	0	0	d1wi4a1(b.36.1.1)	d1jdqa_(d.68.3.3)	d2d0oa1(c.8.6.1)	d1wi4a1(b.36.1.1)	d2cssa1(b.36.1.1)	d1tp5a1(b.36.1.1)
d1djqa1	c.1.4.1	341	8	1	1	d1rd5a_(c.1.2.4)	d1wbha1(c.1.10.1)	d1rd5a_(c.1.2.4)	d1kkoa1(c.1.11.2)	d1i4na_(c.1.2.4)	d1vc4a_(c.1.2.4)
d1ejda_	d.68.2.2	420	8	0	0	d1m6sa_(c.67.1.1)	d1m6sa_(c.67.1.1)	d1ps9a1(c.1.4.1)	d1iuga_(c.67.1.3)	d1u08a_(c.67.1.1)	d1m32a_(c.67.1.3)
d1eoka_	c.1.8.5	283	9	1	1	d1ps9a1(c.1.4.1)	d1djqa1(c.1.4.1)	d1ps9a1(c.1.4.1)	d2b3ya1(c.8.2.1)	d2hoxa1(c.67.1.1)	d2r5ea1(c.67.1.1)
d1g6sa_	d.68.2.2	428	8	0	0	d1m32a_(c.67.1.3)	d1m32a_(c.67.1.3)	d2r5ea1(c.67.1.1)	d1u08a_(c.67.1.1)	d1m6sa_(c.67.1.1)	d1iuga_(c.67.1.3)
d1hc7a3	d.68.5.1	75	9	1	1	d1msza_(d.68.7.1)	d1msza_(d.68.7.1)	d2bkya1(d.68.6.1)	d1kj6a_(g.9.1.1)	d1r6ja_(b.36.1.1)	d1hdmb2(d.19.1.1)
d1hl2a_	c.1.10.1	296	7	1	1	d1rd5a_(c.1.2.4)	d1rd5a_(c.1.2.4)	d1ps9a1(c.1.4.1)	d1i4na_(c.1.2.4)	d1djqa1(c.1.4.1)	d1c7na_(c.67.1.3)
d1i4na_	c.1.2.4	252	7	1	1	d1wbha1(c.1.10.1)	d1djqa1(c.1.4.1)	d1to3a_(c.1.10.1)	d1wbha1(c.1.10.1)	d1hl2a_(c.1.10.1)	d1cl1a_(c.67.1.3)
d1jdqa_	d.68.3.3	99	9	1	1	d1msza_(d.68.7.1)	d1msza_(d.68.7.1)	d1jo0a_(d.68.4.1)	d1eq3a_(d.26.1.1)	d1uewa_(b.36.1.1)	d1m5ya3(d.26.1.1)
d1jo0a_	d.68.4.1	98	10	0	1	d1kida_(c.8.5.1)	d1jdqa_(d.68.3.3)	d1ttea1(a.5.2.1)	d1kida_(c.8.5.1)	d1hl2a_(c.1.10.1)	d1e29a_(a.3.1.1)
d1kbla2	c.8.1.1	134	11	1	1	d2hi6a1(c.8.2.3)	d2isba1(c.8.9.1)	d1nxja_(c.8.7.1)	d2hi6a1(c.8.2.3)	d2b3ya1(c.8.2.1)	d1wbha1(c.1.10.1)
d1kida_	c.8.5.1	194	10	0	1	d1i4na_(c.1.2.4)	d2d0oa1(c.8.6.1)	d1nija1(c.37.1.10)	d1jj7a_(c.37.1.12)	d1i4na_(c.1.2.4)	d1wbha1(c.1.10.1)
d1kkoa1	c.1.11.2	252	9	1	1	d1djqa1(c.1.4.1)	d1vc4a_(c.1.2.4)	d1djqa1(c.1.4.1)	d1wbha1(c.1.10.1)	d1i4na_(c.1.2.4)	d1ps9a1(c.1.4.1)
d1l5ja2	c.8.2.1	213	9	1	1	d1kbla2(c.8.1.1)	d1nxja_(c.8.7.1)	d2d0oa1(c.8.6.1)	d1kbla2(c.8.1.1)	d1g6oa_(c.37.1.11)	d1rd5a_(c.1.2.4)
d1msza_	d.68.7.1	63	9	0	1	d1ttea1(a.5.2.1)	d1nj8a2(d.68.5.1)	d1pava_(d.68.3.3)	d1jdqa_(d.68.3.3)	d1ttea1(a.5.2.1)	d1v92a_(a.5.2.3)
d1nj8a2	d.68.5.1	63	9	0	1	d1m5ya3(d.26.1.1)	d1whra_(d.68.7.1)	d1msza_(d.68.7.1)	d2bkya1(d.68.6.1)	d1eq3a_(d.26.1.1)	d1m5ya3(d.26.1.1)
d1nxja_	c.8.7.1	157	11	1	1	d1kbla2(c.8.1.1)	d2b3ya1(c.8.2.1)	d1kbla2(c.8.1.1)	d1r61a_(c.8.8.1)	d2isba1(c.8.9.1)	d1pava_(d.68.3.3)
d1pava_	d.68.3.3	79	9	1	1	d1msza_(d.68.7.1)	d1msza_(d.68.7.1)	d1hxva_(d.26.1.1)	d1eq3a_(d.26.1.1)	d1m5ya3(d.26.1.1)	d1r6ja_(b.36.1.1)
d1ps9a1	c.1.4.1	331	8	1	1	d1rd5a_(c.1.2.4)	d1wbha1(c.1.10.1)	d1vc4a_(c.1.2.4)	d1to3a_(c.1.10.1)	d1hl2a_(c.1.10.1)	d1rd5a_(c.1.2.4)
d1q3qa2	c.8.5.2	154	10	1	1	d1kbla2(c.8.1.1)	d2isba1(c.8.9.1)	d1a9xb1(c.8.3.1)	d1kbla2(c.8.1.1)	d1lqva_(d.19.1.1)	d1wbha1(c.1.10.1)
d1r61a_	c.8.8.1	206	10	1	1	d1nxja_(c.8.7.1)	d1a9xb1(c.8.3.1)	d1nxja_(c.8.7.1)	d1q3oa_(b.36.1.1)	d1nija1(c.37.1.10)	d1m32a_(c.67.1.3)
d1rd5a_	c.1.2.4	262	7	1	1	d1wbha1(c.1.10.1)	d1to3a_(c.1.10.1)	d1hl2a_(c.1.10.1)	d1ps9a1(c.1.4.1)	d1wbha1(c.1.10.1)	d1djqa1(c.1.4.1)
d1rf6a_	d.68.2.2	428	8	0	0	d1m32a_(c.67.1.3)	d1u08a_(c.67.1.1)	d1m32a_(c.67.1.3)	d2ctza1(c.67.1.3)	d1iuga_(c.67.1.3)	d1cl1a_(c.67.1.3)
d1to3a_	c.1.10.1	292	7	1	1	d1rd5a_(c.1.2.4)	d1rd5a_(c.1.2.4)	d1vc4a_(c.1.2.4)	d1ps9a1(c.1.4.1)	d1i4na_(c.1.2.4)	d1kkoa1(c.1.11.2)
d1vc4a_	c.1.2.4	255	7	1	1	d1wbha1(c.1.10.1)	d1to3a_(c.1.10.1)	d1djqa1(c.1.4.1)	d1kkoa1(c.1.11.2)	d1ps9a1(c.1.4.1)	d1wbha1(c.1.10.1)
d1wbha1	c.1.10.1	214	7	1	1	d1vc4a_(c.1.2.4)	d1rd5a_(c.1.2.4)	d1ps9a1(c.1.4.1)	d1vc4a_(c.1.2.4)	d1djqa1(c.1.4.1)	d1i4na_(c.1.2.4)
d1whra_	d.68.7.1	125	9	0	1	d1kida_(c.8.5.1)	d1jo0a_(d.68.4.1)	d1ttea1(a.5.2.1)	d1e29a_(a.3.1.1)	d1kida_(c.8.5.1)	d1i8oa_(a.3.1.1)
d2b0aa1	c.8.8.1	187	10	1	1	d1a9xb1(c.8.3.1)	d1a9xb1(c.8.3.1)	d2b3ya1(c.8.2.1)	d1pava_(d.68.3.3)	d1u08a_(c.67.1.1)	d1i4na_(c.1.2.4)
d2b3ya1	c.8.2.1	260	9	0	1	d1i4na_(c.1.2.4)	d1kida_(c.8.5.1)	d1djqa1(c.1.4.1)	d1eoka_(c.1.8.5)	d1vc4a_(c.1.2.4)	d1i4na_(c.1.2.4)
d2bkya1	d.68.6.1	90	10	1	1	d1hc7a3(d.68.5.1)	d1hc7a3(d.68.5.1)	d1hxva_(d.26.1.1)	d1q3oa_(b.36.1.1)	d1eq3a_(d.26.1.1)	d1rzxa_(b.36.1.1)
d2d0oa1	c.8.6.1	163	11	1	1	d2hi6a1(c.8.2.3)	d2hi6a1(c.8.2.3)	d1kida_(c.8.5.1)	d1nija1(c.37.1.10)	d1byia_(c.37.1.10)	d2f23a2(d.26.1.2)
d2gyck1	d.41.4.2	132	2	0	0	d2cssa1(b.36.1.1)	d2cssa1(b.36.1.1)	d1wi4a1(b.36.1.1)	d1tp5a1(b.36.1.1)	d1va8a1(b.36.1.1)	d2d0oa1(c.8.6.1)
d2hi6a1	c.8.2.3	133	9	1	1	d1kbla2(c.8.1.1)	d1nxja_(c.8.7.1)	d1kbla2(c.8.1.1)	d1a9xb1(c.8.3.1)	d2d0oa1(c.8.6.1)	d1pava_(d.68.3.3)
d2isba1	c.8.9.1	179	11	0	1	d1m32a_(c.67.1.3)	d1q3qa2(c.8.5.2)	d1nxja_(c.8.7.1)	d1kbla2(c.8.1.1)	d1m32a_(c.67.1.3)	d1rf6a_(d.68.2.2)
d2tpta3	d.41.3.1	106	1	0	1	d1wi4a1(b.36.1.1)	d2gyck1(d.41.4.2)	d1tp5a1(b.36.1.1)	d2cssa1(b.36.1.1)	d1nj8a2(d.68.5.1)	d1wi4a1(b.36.1.1)
**Total Hit**				**22**	**31**						
**Total seq**				**36**	**36**						
**Success Rate**				**61.11%**	**86.11%**						

Prediction results on SCOP_TEST by DN-FoldR

Family											
**Name**	**SCOP ID**	**Length**	**Member**	**Rank1 Hit**	**Rank5 Hit**	**Rank1 List**	**Rank5 List**				
d1b9ha_	c.67.1.4	385	2	1	1	d1o69a_(c.67.1.4)	d1o69a_(c.67.1.4)	d1m6sa_(c.67.1.1)	d1cl1a_(c.67.1.3)	d2ctza1(c.67.1.3)	d1m32a_(c.67.1.3)
d1brwa3	d.41.3.1	104	1	1	1	d2tpta3(d.41.3.1)	d2tpta3(d.41.3.1)	d1g9oa_(b.36.1.1)	d1wi4a1(b.36.1.1)	d2cssa1(b.36.1.1)	d1tp5a1(b.36.1.1)
d1byia_	c.37.1.10	225	2	1	1	d1nija1(c.37.1.10)	d1vcoa2(c.37.1.10)	d1nija1(c.37.1.10)	d1kkoa1(c.1.11.2)	d1wbha1(c.1.10.1)	d1djqa1(c.1.4.1)
d1c7na_	c.67.1.3	395	4	0	1	d1u08a_(c.67.1.1)	d1m32a_(c.67.1.3)	d1m6sa_(c.67.1.1)	d2r5ea1(c.67.1.1)	d2hoxa1(c.67.1.1)	d1u08a_(c.67.1.1)
d1cl1a_	c.67.1.3	392	4	1	1	d2ctza1(c.67.1.3)	d1m32a_(c.67.1.3)	d1iuga_(c.67.1.3)	d2ctza1(c.67.1.3)	d1b9ha_(c.67.1.4)	d1o69a_(c.67.1.4)
d1ctja_	a.3.1.1	90	6	1	1	d1wvec1(a.3.1.1)	d2c8sa1(a.3.1.1)	d1wvec1(a.3.1.1)	d1i8oa_(a.3.1.1)	d1dw0a_(a.3.1.1)	d1e29a_(a.3.1.1)
d1djqa1	c.1.4.1	341	1	1	1	d1ps9a1(c.1.4.1)	d1ps9a1(c.1.4.1)	d1cl1a_(c.67.1.3)	d1rd5a_(c.1.2.4)	d1vc4a_(c.1.2.4)	d1hl2a_(c.1.10.1)
d1dw0a_	a.3.1.1	113	6	1	1	d1gu2a_(a.3.1.1)	d1gu2a_(a.3.1.1)	d2c8sa1(a.3.1.1)	d1e29a_(a.3.1.1)	d1i8oa_(a.3.1.1)	d1ctja_(a.3.1.1)
d1e29a_	a.3.1.1	136	6	1	1	d1ctja_(a.3.1.1)	d1wvec1(a.3.1.1)	d2c8sa1(a.3.1.1)	d1i8oa_(a.3.1.1)	d1ctja_(a.3.1.1)	d1gu2a_(a.3.1.1)
d1ejda_	d.68.2.2	420	2	1	1	d1g6sa_(d.68.2.2)	d1g6sa_(d.68.2.2)	d1rf6a_(d.68.2.2)	d1m6sa_(c.67.1.1)	d1iuga_(c.67.1.3)	d1m32a_(c.67.1.3)
d1eq3a_	d.26.1.1	97	6	1	1	d1m5ya3(d.26.1.1)	d1t11a3(d.26.1.1)	d1m5ya3(d.26.1.1)	d1hxva_(d.26.1.1)	d1pava_(d.68.3.3)	d1nj8a2(d.68.5.1)
d1ewsa_	g.9.1.1	33	3	1	1	d1ijva_(g.9.1.1)	d1ijva_(g.9.1.1)	d1kj6a_(g.9.1.1)	d1zuea1(g.9.1.1)	d1msza_(d.68.7.1)	d1nj8a2(d.68.5.1)
d1g3ka_	d.153.1.4	174	7	1	1	d1ryph_(d.153.1.4)	d1ryph_(d.153.1.4)	d1rypg_(d.153.1.4)	d1ryp2_(d.153.1.4)	d1yara1(d.153.1.4)	d1rypc_(d.153.1.4)
d1g6sa_	d.68.2.2	428	2	1	1	d1rf6a_(d.68.2.2)	d1ejda_(d.68.2.2)	d1rf6a_(d.68.2.2)	d1m32a_(c.67.1.3)	d1m6sa_(c.67.1.1)	d1iuga_(c.67.1.3)
d1g9oa_	b.36.1.1	92	10	1	1	d1tp5a1(b.36.1.1)	d1rzxa_(b.36.1.1)	d1r6ja_(b.36.1.1)	d1tp5a1(b.36.1.1)	d1va8a1(b.36.1.1)	d1rgwa_(b.36.1.1)
d1gu2a_	a.3.1.1	125	6	1	1	d1dw0a_(a.3.1.1)	d1i8oa_(a.3.1.1)	d1e29a_(a.3.1.1)	d2c8sa1(a.3.1.1)	d1ctja_(a.3.1.1)	d1dw0a_(a.3.1.1)
d1hc7a3	d.68.5.1	75	1	1	1	d1nj8a2(d.68.5.1)	d1nj8a2(d.68.5.1)	d1va8a1(b.36.1.1)	d1msza_(d.68.7.1)	d1r6ja_(b.36.1.1)	d2bkya1(d.68.6.1)
d1hdma2	d.19.1.1	82	6	1	1	d1hdmb2(d.19.1.1)	d1hdmb2(d.19.1.1)	d1uvqb2(d.19.1.1)	d1rzxa_(b.36.1.1)	d1va8a1(b.36.1.1)	d1tp5a1(b.36.1.1)
d1hdmb2	d.19.1.1	86	6	1	1	d1uvqb2(d.19.1.1)	d1hdma2(d.19.1.1)	d1uvqb2(d.19.1.1)	d1lqva_(d.19.1.1)	d1tp5a1(b.36.1.1)	d1wi4a1(b.36.1.1)
d1hl2a_	c.1.10.1	296	2	0	1	d1ps9a1(c.1.4.1)	d1wbha1(c.1.10.1)	d1rd5a_(c.1.2.4)	d1b9ha_(c.67.1.4)	d1ps9a1(c.1.4.1)	d1i4na_(c.1.2.4)
d1hxva_	d.26.1.1	86	6	1	1	d1t11a3(d.26.1.1)	d1jvwa_(d.26.1.1)	d1u79a_(d.26.1.1)	d1t11a3(d.26.1.1)	d1q6ha_(d.26.1.1)	d2f23a2(d.26.1.2)
d1hyrc2	d.19.1.1	182	6	1	1	d1t7va2(d.19.1.1)	d1t7va2(d.19.1.1)	d1lqva_(d.19.1.1)	d2h26a2(d.19.1.1)	d1kida_(c.8.5.1)	d1g3ka_(d.153.1.4)
d1i4na_	c.1.2.4	252	2	1	1	d1vc4a_(c.1.2.4)	d1rd5a_(c.1.2.4)	d1vc4a_(c.1.2.4)	d1to3a_(c.1.10.1)	d1wbha1(c.1.10.1)	d1hl2a_(c.1.10.1)
d1i8oa_	a.3.1.1	115	6	1	1	d1e29a_(a.3.1.1)	d1wvec1(a.3.1.1)	d1e29a_(a.3.1.1)	d1dw0a_(a.3.1.1)	d2c8sa1(a.3.1.1)	d1ctja_(a.3.1.1)
d1ijva_	g.9.1.1	37	3	1	1	d1kj6a_(g.9.1.1)	d1kj6a_(g.9.1.1)	d1zuea1(g.9.1.1)	d1ewsa_(g.9.1.1)	d1ttea1(a.5.2.1)	d1nj8a2(d.68.5.1)
d1iuga_	c.67.1.3	349	4	1	1	d1m32a_(c.67.1.3)	d1m32a_(c.67.1.3)	d2r5ea1(c.67.1.1)	d1v72a1(c.67.1.1)	d1m6sa_(c.67.1.1)	d1o69a_(c.67.1.4)
d1jdqa_	d.68.3.3	99	1	1	1	d1pava_(d.68.3.3)	d1pava_(d.68.3.3)	d1eq3a_(d.26.1.1)	d1hxva_(d.26.1.1)	d1m5ya3(d.26.1.1)	d1u79a_(d.26.1.1)
d1jj7a_	c.37.1.12	252	3	1	1	d3dhwc1(c.37.1.12)	d3dhwc1(c.37.1.12)	d2onka1(c.37.1.12)	d1l2ta_(c.37.1.12)	d1nija1(c.37.1.10)	d1g6oa_(c.37.1.11)
d1jvwa_	d.26.1.1	161	6	1	1	d1q6ha_(d.26.1.1)	d1u79a_(d.26.1.1)	d1t11a3(d.26.1.1)	d1q6ha_(d.26.1.1)	d1hxva_(d.26.1.1)	d1kbla2(c.8.1.1)
d1kj6a_	g.9.1.1	46	3	1	1	d1ijva_(g.9.1.1)	d1zuea1(g.9.1.1)	d1ewsa_(g.9.1.1)	d1ijva_(g.9.1.1)	d1xb2b1(a.5.2.2)	d1v92a_(a.5.2.3)
d1l2ta_	c.37.1.12	231	3	1	1	d3dhwc1(c.37.1.12)	d3dhwc1(c.37.1.12)	d1jj7a_(c.37.1.12)	d2onka1(c.37.1.12)	d1nija1(c.37.1.10)	d1kida_(c.8.5.1)
d1l5ja2	c.8.2.1	213	1	1	1	d2b3ya1(c.8.2.1)	d2b3ya1(c.8.2.1)	d1wbha1(c.1.10.1)	d2hi6a1(c.8.2.3)	d1byia_(c.37.1.10)	d1kbla2(c.8.1.1)
d1lqva_	d.19.1.1	173	6	1	1	d2h26a2(d.19.1.1)	d2h26a2(d.19.1.1)	d1t7va2(d.19.1.1)	d1uvqb2(d.19.1.1)	d1hyrc2(d.19.1.1)	d1g3ka_(d.153.1.4)
d1m32a_	c.67.1.3	362	4	1	1	d1iuga_(c.67.1.3)	d1iuga_(c.67.1.3)	d1c7na_(c.67.1.3)	d1m6sa_(c.67.1.1)	d1o69a_(c.67.1.4)	d1v72a1(c.67.1.1)
d1m5ya3	d.26.1.1	109	6	1	1	d1eq3a_(d.26.1.1)	d1hxva_(d.26.1.1)	d1t11a3(d.26.1.1)	d1eq3a_(d.26.1.1)	d1tp5a1(b.36.1.1)	d1jdqa_(d.68.3.3)
d1m6sa_	c.67.1.1	344	4	1	1	d1v72a1(c.67.1.1)	d1v72a1(c.67.1.1)	d1c7na_(c.67.1.3)	d1m32a_(c.67.1.3)	d1ohwa_(c.67.1.4)	d1iuga_(c.67.1.3)
d1mn3a_	a.5.2.4	55	1	0	1	d1wgna_(a.5.2.1)	d1otra_(a.5.2.4)	d2bwba1(a.5.2.1)	d1wgna_(a.5.2.1)	d2dnaa1(a.5.2.1)	d1wiva_(a.5.2.1)
d1msza_	d.68.7.1	63	1	1	1	d1whra_(d.68.7.1)	d1whra_(d.68.7.1)	d1oaia_(a.5.2.3)	d2bkya1(d.68.6.1)	d1ttea1(a.5.2.1)	d1pava_(d.68.3.3)
d1nija1	c.37.1.10	223	2	1	1	d1byia_(c.37.1.10)	d1byia_(c.37.1.10)	d2onka1(c.37.1.12)	d3dhwc1(c.37.1.12)	d1kida_(c.8.5.1)	d1l2ta_(c.37.1.12)
d1nj8a2	d.68.5.1	63	1	1	1	d1hc7a3(d.68.5.1)	d1hc7a3(d.68.5.1)	d1msza_(d.68.7.1)	d1eq3a_(d.26.1.1)	d2f23a2(d.26.1.2)	d1m5ya3(d.26.1.1)
d1o69a_	c.67.1.4	375	2	1	1	d1b9ha_(c.67.1.4)	d1b9ha_(c.67.1.4)	d1m32a_(c.67.1.3)	d1iuga_(c.67.1.3)	d1cl1a_(c.67.1.3)	d2ctza1(c.67.1.3)
d1oaia_	a.5.2.3	60	1	1	1	d1v92a_(a.5.2.3)	d1v92a_(a.5.2.3)	d1xb2b1(a.5.2.2)	d3e46a1(a.5.2.1)	d2bwba1(a.5.2.1)	d1wgna_(a.5.2.1)
d1ohwa_	c.67.1.4	462	2	0	0	d1m6sa_(c.67.1.1)	d1m6sa_(c.67.1.1)	d1m32a_(c.67.1.3)	d1iuga_(c.67.1.3)	d2r5ea1(c.67.1.1)	d1v72a1(c.67.1.1)
d1otra_	a.5.2.4	50	1	0	0	d1wgna_(a.5.2.1)	d2bwba1(a.5.2.1)	d1wgna_(a.5.2.1)	d1wiva_(a.5.2.1)	d2cpwa1(a.5.2.1)	d2dnaa1(a.5.2.1)
d1pava_	d.68.3.3	79	1	1	1	d1jdqa_(d.68.3.3)	d1jdqa_(d.68.3.3)	d1eq3a_(d.26.1.1)	d2f23a2(d.26.1.2)	d1msza_(d.68.7.1)	d1m5ya3(d.26.1.1)
d1pgya_	a.5.2.1	48	14	1	1	d1wgna_(a.5.2.1)	d1wiva_(a.5.2.1)	d1wgna_(a.5.2.1)	d3e46a1(a.5.2.1)	d2dnaa1(a.5.2.1)	d1oaia_(a.5.2.3)
d1ps9a1	c.1.4.1	331	1	1	1	d1djqa1(c.1.4.1)	d1djqa1(c.1.4.1)	d1vc4a_(c.1.2.4)	d1to3a_(c.1.10.1)	d1hl2a_(c.1.10.1)	d1rd5a_(c.1.2.4)
d1q3oa_	b.36.1.1	105	10	1	1	d1rzxa_(b.36.1.1)	d1rzxa_(b.36.1.1)	d1r6ja_(b.36.1.1)	d1g9oa_(b.36.1.1)	d1tp5a1(b.36.1.1)	d1va8a1(b.36.1.1)
d1q6ha_	d.26.1.1	211	6	1	1	d1jvwa_(d.26.1.1)	d1t11a3(d.26.1.1)	d1jvwa_(d.26.1.1)	d1u79a_(d.26.1.1)	d1hxva_(d.26.1.1)	d1rypb_(d.153.1.4)
d1r61a_	c.8.8.1	206	1	1	1	d2b0aa1(c.8.8.1)	d2b0aa1(c.8.8.1)	d1a9xb1(c.8.3.1)	d2b3ya1(c.8.2.1)	d1nxja_(c.8.7.1)	d1to3a_(c.1.10.1)
d1r6ja_	b.36.1.1	83	10	1	1	d1q3oa_(b.36.1.1)	d1rzxa_(b.36.1.1)	d1rgwa_(b.36.1.1)	d1g9oa_(b.36.1.1)	d1q3oa_(b.36.1.1)	d1va8a1(b.36.1.1)
d1rd5a_	c.1.2.4	262	2	1	1	d1i4na_(c.1.2.4)	d1i4na_(c.1.2.4)	d1vc4a_(c.1.2.4)	d1to3a_(c.1.10.1)	d1hl2a_(c.1.10.1)	d1wbha1(c.1.10.1)
d1rf6a_	d.68.2.2	428	2	1	1	d1g6sa_(d.68.2.2)	d1ejda_(d.68.2.2)	d1g6sa_(d.68.2.2)	d1u08a_(c.67.1.1)	d1m32a_(c.67.1.3)	d1iuga_(c.67.1.3)
d1rgwa_	b.36.1.1	86	10	1	1	d1q3oa_(b.36.1.1)	d1tp5a1(b.36.1.1)	d1va8a1(b.36.1.1)	d1rzxa_(b.36.1.1)	d1g9oa_(b.36.1.1)	d1q3oa_(b.36.1.1)
d1ryp2_	d.153.1.4	234	7	1	1	d1ryph_(d.153.1.4)	d1rypc_(d.153.1.4)	d1ryph_(d.153.1.4)	d1rypg_(d.153.1.4)	d1rype_(d.153.1.4)	d1yara1(d.153.1.4)
d1rypb_	d.153.1.4	251	7	1	1	d1rypc_(d.153.1.4)	d1ryp2_(d.153.1.4)	d1rypc_(d.153.1.4)	d1rype_(d.153.1.4)	d1rypg_(d.153.1.4)	d1yara1(d.153.1.4)
d1rypc_	d.153.1.4	245	7	1	1	d1rypg_(d.153.1.4)	d1yara1(d.153.1.4)	d1ryp2_(d.153.1.4)	d1rypg_(d.153.1.4)	d1rypb_(d.153.1.4)	d1rype_(d.153.1.4)
d1rype_	d.153.1.4	243	7	1	1	d1rypg_(d.153.1.4)	d1rypg_(d.153.1.4)	d1ryp2_(d.153.1.4)	d1yara1(d.153.1.4)	d1rypb_(d.153.1.4)	d1rypc_(d.153.1.4)
d1rypg_	d.153.1.4	245	7	1	1	d1rypc_(d.153.1.4)	d1yara1(d.153.1.4)	d1rype_(d.153.1.4)	d1rypc_(d.153.1.4)	d1ryp2_(d.153.1.4)	d1rypb_(d.153.1.4)
d1ryph_	d.153.1.4	206	7	1	1	d1ryp2_(d.153.1.4)	d1ryp2_(d.153.1.4)	d1yara1(d.153.1.4)	d1rype_(d.153.1.4)	d1g3ka_(d.153.1.4)	d1rypc_(d.153.1.4)
d1rzxa_	b.36.1.1	99	10	1	1	d1q3oa_(b.36.1.1)	d1q3oa_(b.36.1.1)	d1tp5a1(b.36.1.1)	d1g9oa_(b.36.1.1)	d1uewa_(b.36.1.1)	d1va8a1(b.36.1.1)
d1t11a3	d.26.1.1	114	6	1	1	d1hxva_(d.26.1.1)	d1q6ha_(d.26.1.1)	d1hxva_(d.26.1.1)	d1u79a_(d.26.1.1)	d1jvwa_(d.26.1.1)	d2f23a2(d.26.1.2)
d1t7va2	d.19.1.1	180	6	1	1	d2h26a2(d.19.1.1)	d2h26a2(d.19.1.1)	d1uvqb2(d.19.1.1)	d1hyrc2(d.19.1.1)	d1lqva_(d.19.1.1)	d1g3ka_(d.153.1.4)
d1to3a_	c.1.10.1	292	2	0	0	d1rd5a_(c.1.2.4)	d1rd5a_(c.1.2.4)	d1vc4a_(c.1.2.4)	d1m6sa_(c.67.1.1)	d1i4na_(c.1.2.4)	d1kkoa1(c.1.11.2)
d1tp5a1	b.36.1.1	103	10	1	1	d1g9oa_(b.36.1.1)	d1va8a1(b.36.1.1)	d1g9oa_(b.36.1.1)	d1wi4a1(b.36.1.1)	d1rzxa_(b.36.1.1)	d1uewa_(b.36.1.1)
d1ttea1	a.5.2.1	56	14	1	1	d1wgna_(a.5.2.1)	d1veka_(a.5.2.1)	d1wgna_(a.5.2.1)	d1wiva_(a.5.2.1)	d2cpwa1(a.5.2.1)	d2crna1(a.5.2.1)
d1u08a_	c.67.1.1	383	4	1	1	d2r5ea1(c.67.1.1)	d2r5ea1(c.67.1.1)	d1m6sa_(c.67.1.1)	d1v72a1(c.67.1.1)	d2hoxa1(c.67.1.1)	d1c7na_(c.67.1.3)
d1u79a_	d.26.1.1	126	6	1	1	d1jvwa_(d.26.1.1)	d1jvwa_(d.26.1.1)	d1q6ha_(d.26.1.1)	d1hxva_(d.26.1.1)	d1t11a3(d.26.1.1)	d1g9oa_(b.36.1.1)
d1uewa_	b.36.1.1	115	10	1	1	d1tp5a1(b.36.1.1)	d1g9oa_(b.36.1.1)	d1rzxa_(b.36.1.1)	d1va8a1(b.36.1.1)	d1q3oa_(b.36.1.1)	d1tp5a1(b.36.1.1)
d1uvqb2	d.19.1.1	93	6	1	1	d1hdmb2(d.19.1.1)	d1t7va2(d.19.1.1)	d1hdmb2(d.19.1.1)	d1lqva_(d.19.1.1)	d1hyrc2(d.19.1.1)	d1tp5a1(b.36.1.1)
d1v72a1	c.67.1.1	346	4	1	1	d1m6sa_(c.67.1.1)	d2r5ea1(c.67.1.1)	d1m6sa_(c.67.1.1)	d1m32a_(c.67.1.3)	d1c7na_(c.67.1.3)	d1iuga_(c.67.1.3)
d1v92a_	a.5.2.3	47	1	1	1	d1oaia_(a.5.2.3)	d1oaia_(a.5.2.3)	d1wgna_(a.5.2.1)	d2bwba1(a.5.2.1)	d1xb2b1(a.5.2.2)	d2dnaa1(a.5.2.1)
d1va8a1	b.36.1.1	101	10	1	1	d1tp5a1(b.36.1.1)	d1g9oa_(b.36.1.1)	d1rzxa_(b.36.1.1)	d1tp5a1(b.36.1.1)	d1q3oa_(b.36.1.1)	d1uewa_(b.36.1.1)
d1vaea_	b.36.1.1	112	10	1	1	d1g9oa_(b.36.1.1)	d1tp5a1(b.36.1.1)	d1g9oa_(b.36.1.1)	d1q3oa_(b.36.1.1)	d2cssa1(b.36.1.1)	d1va8a1(b.36.1.1)
d1vc4a_	c.1.2.4	255	2	1	1	d1i4na_(c.1.2.4)	d1i4na_(c.1.2.4)	d1rd5a_(c.1.2.4)	d1to3a_(c.1.10.1)	d1kkoa1(c.1.11.2)	d1wbha1(c.1.10.1)
d1vcoa2	c.37.1.10	273	2	1	1	d1byia_(c.37.1.10)	d1nija1(c.37.1.10)	d1byia_(c.37.1.10)	d1rd5a_(c.1.2.4)	d1v72a1(c.67.1.1)	d1ps9a1(c.1.4.1)
d1vdla_	a.5.2.1	81	14	1	1	d1wj7a1(a.5.2.1)	d1wgna_(a.5.2.1)	d1wj7a1(a.5.2.1)	d1wiva_(a.5.2.1)	d2dnaa1(a.5.2.1)	d1xb2b1(a.5.2.2)
d1vega_	a.5.2.1	84	14	1	1	d1veka_(a.5.2.1)	d1wiva_(a.5.2.1)	d1veka_(a.5.2.1)	d1wgna_(a.5.2.1)	d2crna1(a.5.2.1)	d1vdla_(a.5.2.1)
d1veka_	a.5.2.1	85	14	1	1	d1wiva_(a.5.2.1)	d1wiva_(a.5.2.1)	d1vega_(a.5.2.1)	d2cpwa1(a.5.2.1)	d2crna1(a.5.2.1)	d1wgna_(a.5.2.1)
d1wbha1	c.1.10.1	214	2	0	1	d1vc4a_(c.1.2.4)	d1hl2a_(c.1.10.1)	d1kkoa1(c.1.11.2)	d1rd5a_(c.1.2.4)	d1vc4a_(c.1.2.4)	d1i4na_(c.1.2.4)
d1wgna_	a.5.2.1	64	14	1	1	d1wiva_(a.5.2.1)	d2cpwa1(a.5.2.1)	d2dnaa1(a.5.2.1)	d1wiva_(a.5.2.1)	d2crna1(a.5.2.1)	d1veka_(a.5.2.1)
d1whra_	d.68.7.1	125	1	1	1	d1msza_(d.68.7.1)	d1msza_(d.68.7.1)	d1eq3a_(d.26.1.1)	d1e29a_(a.3.1.1)	d2c8sa1(a.3.1.1)	d1jo0a_(d.68.4.1)
d1wi4a1	b.36.1.1	97	10	1	1	d1tp5a1(b.36.1.1)	d1tp5a1(b.36.1.1)	d1va8a1(b.36.1.1)	d1rzxa_(b.36.1.1)	d1uewa_(b.36.1.1)	d2cssa1(b.36.1.1)
d1wiva_	a.5.2.1	74	14	1	1	d1wgna_(a.5.2.1)	d1wgna_(a.5.2.1)	d1veka_(a.5.2.1)	d2crna1(a.5.2.1)	d2cpwa1(a.5.2.1)	d2bwba1(a.5.2.1)
d1wj7a1	a.5.2.1	92	14	1	1	d1veka_(a.5.2.1)	d1vdla_(a.5.2.1)	d1wiva_(a.5.2.1)	d2dnaa1(a.5.2.1)	d1veka_(a.5.2.1)	d1wgna_(a.5.2.1)
d1wvec1	a.3.1.1	75	6	1	1	d1ctja_(a.3.1.1)	d1dw0a_(a.3.1.1)	d1e29a_(a.3.1.1)	d1i8oa_(a.3.1.1)	d1ctja_(a.3.1.1)	d2c8sa1(a.3.1.1)
d1yara1	d.153.1.4	222	7	1	1	d1rypc_(d.153.1.4)	d1rype_(d.153.1.4)	d1rypg_(d.153.1.4)	d1rypb_(d.153.1.4)	d1rypc_(d.153.1.4)	d1ryp2_(d.153.1.4)
d1zuea1	g.9.1.1	43	3	1	1	d1ijva_(g.9.1.1)	d1ewsa_(g.9.1.1)	d1ijva_(g.9.1.1)	d1kj6a_(g.9.1.1)	d1hc7a3(d.68.5.1)	d1oaia_(a.5.2.3)
d2b0aa1	c.8.8.1	187	1	1	1	d1r61a_(c.8.8.1)	d1r61a_(c.8.8.1)	d1a9xb1(c.8.3.1)	d2b3ya1(c.8.2.1)	d2d0oa1(c.8.6.1)	d2isba1(c.8.9.1)
d2b3ya1	c.8.2.1	260	1	1	1	d1l5ja2(c.8.2.1)	d1l5ja2(c.8.2.1)	d1wbha1(c.1.10.1)	d1vc4a_(c.1.2.4)	d1kida_(c.8.5.1)	d1i4na_(c.1.2.4)
d2bwba1	a.5.2.1	45	14	1	1	d2dnaa1(a.5.2.1)	d1wgna_(a.5.2.1)	d3e46a1(a.5.2.1)	d1wiva_(a.5.2.1)	d2cpwa1(a.5.2.1)	d2dnaa1(a.5.2.1)
d2c8sa1	a.3.1.1	150	6	1	1	d1wvec1(a.3.1.1)	d1dw0a_(a.3.1.1)	d1ctja_(a.3.1.1)	d1e29a_(a.3.1.1)	d1i8oa_(a.3.1.1)	d1wvec1(a.3.1.1)
d2cpwa1	a.5.2.1	52	14	1	1	d1wgna_(a.5.2.1)	d2crna1(a.5.2.1)	d1wgna_(a.5.2.1)	d1veka_(a.5.2.1)	d1wiva_(a.5.2.1)	d2bwba1(a.5.2.1)
d2crna1	a.5.2.1	52	14	1	1	d1veka_(a.5.2.1)	d1veka_(a.5.2.1)	d2bwba1(a.5.2.1)	d1wiva_(a.5.2.1)	d1wgna_(a.5.2.1)	d2cpwa1(a.5.2.1)
d2cssa1	b.36.1.1	109	10	1	1	d1rzxa_(b.36.1.1)	d1tp5a1(b.36.1.1)	d1wi4a1(b.36.1.1)	d1va8a1(b.36.1.1)	d1rzxa_(b.36.1.1)	d1q3oa_(b.36.1.1)
d2ctza1	c.67.1.3	422	4	1	1	d1cl1a_(c.67.1.3)	d1iuga_(c.67.1.3)	d1m32a_(c.67.1.3)	d1cl1a_(c.67.1.3)	d1b9ha_(c.67.1.4)	d1o69a_(c.67.1.4)
d2dkla1	a.5.2.1	73	14	1	1	d1wiva_(a.5.2.1)	d1wiva_(a.5.2.1)	d1vdla_(a.5.2.1)	d1veka_(a.5.2.1)	d2dnaa1(a.5.2.1)	d1wgna_(a.5.2.1)
d2dnaa1	a.5.2.1	51	14	1	1	d2bwba1(a.5.2.1)	d3e46a1(a.5.2.1)	d1wiva_(a.5.2.1)	d1wgna_(a.5.2.1)	d2cpwa1(a.5.2.1)	d2bwba1(a.5.2.1)
d2h26a2	d.19.1.1	178	6	1	1	d1lqva_(d.19.1.1)	d1lqva_(d.19.1.1)	d1hyrc2(d.19.1.1)	d1t7va2(d.19.1.1)	d1ryph_(d.153.1.4)	d1g3ka_(d.153.1.4)
d2hoxa1	c.67.1.1	426	4	1	1	d1u08a_(c.67.1.1)	d1u08a_(c.67.1.1)	d2r5ea1(c.67.1.1)	d1c7na_(c.67.1.3)	d1m32a_(c.67.1.3)	d1cl1a_(c.67.1.3)
d2k0bx1	a.5.2.1	53	14	1	1	d2dnaa1(a.5.2.1)	d1wiva_(a.5.2.1)	d3e46a1(a.5.2.1)	d2dnaa1(a.5.2.1)	d1wgna_(a.5.2.1)	d2bwba1(a.5.2.1)
d2onka1	c.37.1.12	241	3	1	1	d3dhwc1(c.37.1.12)	d1l2ta_(c.37.1.12)	d1jj7a_(c.37.1.12)	d3dhwc1(c.37.1.12)	d1g6oa_(c.37.1.11)	d1byia_(c.37.1.10)
d2r5ea1	c.67.1.1	419	4	1	1	d1u08a_(c.67.1.1)	d1v72a1(c.67.1.1)	d1u08a_(c.67.1.1)	d2hoxa1(c.67.1.1)	d1c7na_(c.67.1.3)	d1m32a_(c.67.1.3)

Superfamily											
**Name**	**SCOP ID**	**Length**	**Member**	**Rank1 Hit**	**Rank5 Hit**	**Rank1 List**	**Rank5 List**				
d1a0tp_	f.4.3.2	414	1	1	1	d1t16a_(f.4.3.4)	d1t16a_(f.4.3.4)	d1v72a1(c.67.1.1)	d1rf6a_(d.68.2.2)	d1rypg_(d.153.1.4)	d1ejda_(d.68.2.2)
d1b9ha_	c.67.1.4	385	10	1	1	d2ctza1(c.67.1.3)	d2r5ea1(c.67.1.1)	d1m6sa_(c.67.1.1)	d1cl1a_(c.67.1.3)	d2ctza1(c.67.1.3)	d1m32a_(c.67.1.3)
d1byia_	c.37.1.10	225	5	0	1	d1kkoa1(c.1.11.2)	d2onka1(c.37.1.12)	d1kkoa1(c.1.11.2)	d1wbha1(c.1.10.1)	d1djqa1(c.1.4.1)	d1eoka_(c.1.8.5)
d1c7na_	c.67.1.3	395	8	1	1	d1u08a_(c.67.1.1)	d1v72a1(c.67.1.1)	d1m6sa_(c.67.1.1)	d2r5ea1(c.67.1.1)	d2hoxa1(c.67.1.1)	d1u08a_(c.67.1.1)
d1cl1a_	c.67.1.3	392	8	1	1	d1o69a_(c.67.1.4)	d1u08a_(c.67.1.1)	d2r5ea1(c.67.1.1)	d1m6sa_(c.67.1.1)	d1b9ha_(c.67.1.4)	d1o69a_(c.67.1.4)
d1eq3a_	d.26.1.1	97	1	0	0	d1nj8a2(d.68.5.1)	d1jdqa_(d.68.3.3)	d1brwa3(d.41.3.1)	d1pava_(d.68.3.3)	d1nj8a2(d.68.5.1)	d2tpta3(d.41.3.1)
d1g6oa_	c.37.1.11	324	7	1	1	d1jj7a_(c.37.1.12)	d2onka1(c.37.1.12)	d1l2ta_(c.37.1.12)	d1jj7a_(c.37.1.12)	d3dhwc1(c.37.1.12)	d1m32a_(c.67.1.3)
d1hxva_	d.26.1.1	86	1	1	1	d2f23a2(d.26.1.2)	d2f23a2(d.26.1.2)	d1q3oa_(b.36.1.1)	d1jdqa_(d.68.3.3)	d2bkya1(d.68.6.1)	d1rzxa_(b.36.1.1)
d1iuga_	c.67.1.3	349	8	1	1	d1v72a1(c.67.1.1)	d2r5ea1(c.67.1.1)	d1v72a1(c.67.1.1)	d1m6sa_(c.67.1.1)	d1o69a_(c.67.1.4)	d1b9ha_(c.67.1.4)
d1jj7a_	c.37.1.12	252	4	1	1	d1g6oa_(c.37.1.11)	d1nija1(c.37.1.10)	d1g6oa_(c.37.1.11)	d1ryp2_(d.153.1.4)	d1rypc_(d.153.1.4)	d1vc4a_(c.1.2.4)
d1jvwa_	d.26.1.1	161	1	0	0	d1kbla2(c.8.1.1)	d1a9xb1(c.8.3.1)	d1kbla2(c.8.1.1)	d1q3qa2(c.8.5.2)	d1va8a1(b.36.1.1)	d1q3oa_(b.36.1.1)
d1kida_	c.8.5.1	194	1	1	1	d1q3qa2(c.8.5.2)	d1q3qa2(c.8.5.2)	d1nija1(c.37.1.10)	d2b3ya1(c.8.2.1)	d1l2ta_(c.37.1.12)	d1wbha1(c.1.10.1)
d1l2ta_	c.37.1.12	231	4	1	1	d1nija1(c.37.1.10)	d1g6oa_(c.37.1.11)	d1nija1(c.37.1.10)	d1l5ja2(c.8.2.1)	d1kida_(c.8.5.1)	d1ryph_(d.153.1.4)
d1l5ja2	c.8.2.1	213	1	0	1	d1wbha1(c.1.10.1)	d2hi6a1(c.8.2.3)	d1wbha1(c.1.10.1)	d1byia_(c.37.1.10)	d1l2ta_(c.37.1.12)	d1kbla2(c.8.1.1)
d1m32a_	c.67.1.3	362	8	1	1	d1v72a1(c.67.1.1)	d1m6sa_(c.67.1.1)	d1b9ha_(c.67.1.4)	d2r5ea1(c.67.1.1)	d1o69a_(c.67.1.4)	d1v72a1(c.67.1.1)
d1m5ya3	d.26.1.1	109	1	0	0	d1jdqa_(d.68.3.3)	d1wvec1(a.3.1.1)	d1tp5a1(b.36.1.1)	d1pava_(d.68.3.3)	d1jdqa_(d.68.3.3)	d1nj8a2(d.68.5.1)
d1m6sa_	c.67.1.1	344	8	1	1	d1ohwa_(c.67.1.4)	d1c7na_(c.67.1.3)	d1o69a_(c.67.1.4)	d1m32a_(c.67.1.3)	d1ohwa_(c.67.1.4)	d1iuga_(c.67.1.3)
d1mn3a_	a.5.2.4	55	18	1	1	d1wgna_(a.5.2.1)	d2bwba1(a.5.2.1)	d2k0bx1(a.5.2.1)	d1wgna_(a.5.2.1)	d2dnaa1(a.5.2.1)	d1wiva_(a.5.2.1)
d1nija1	c.37.1.10	223	5	1	1	d2onka1(c.37.1.12)	d2onka1(c.37.1.12)	d3dhwc1(c.37.1.12)	d1l2ta_(c.37.1.12)	d1kida_(c.8.5.1)	d1rd5a_(c.1.2.4)
d1o69a_	c.67.1.4	375	10	1	1	d2ctza1(c.67.1.3)	d1m32a_(c.67.1.3)	d1iuga_(c.67.1.3)	d1cl1a_(c.67.1.3)	d2ctza1(c.67.1.3)	d1m6sa_(c.67.1.1)
d1oaia_	a.5.2.3	60	18	1	1	d1xb2b1(a.5.2.2)	d1xb2b1(a.5.2.2)	d1vdla_(a.5.2.1)	d3e46a1(a.5.2.1)	d2bwba1(a.5.2.1)	d1wgna_(a.5.2.1)
d1ohwa_	c.67.1.4	462	10	1	1	d1m6sa_(c.67.1.1)	d1m6sa_(c.67.1.1)	d1m32a_(c.67.1.3)	d1iuga_(c.67.1.3)	d2r5ea1(c.67.1.1)	d1v72a1(c.67.1.1)
d1otra_	a.5.2.4	50	18	1	1	d1wgna_(a.5.2.1)	d2bwba1(a.5.2.1)	d1wgna_(a.5.2.1)	d1wiva_(a.5.2.1)	d2cpwa1(a.5.2.1)	d2dnaa1(a.5.2.1)
d1pgya_	a.5.2.1	48	5	1	1	d1oaia_(a.5.2.3)	d1v92a_(a.5.2.3)	d1oaia_(a.5.2.3)	d1mn3a_(a.5.2.4)	d1xb2b1(a.5.2.2)	d1otra_(a.5.2.4)
d1q3qa2	c.8.5.2	154	1	0	1	d1kbla2(c.8.1.1)	d1kida_(c.8.5.1)	d2isba1(c.8.9.1)	d1a9xb1(c.8.3.1)	d1kbla2(c.8.1.1)	d1wbha1(c.1.10.1)
d1q6ha_	d.26.1.1	211	1	0	0	d1rypb_(d.153.1.4)	d1byia_(c.37.1.10)	d1rypb_(d.153.1.4)	d1ryph_(d.153.1.4)	d1rypg_(d.153.1.4)	d1rype_(d.153.1.4)
d1t11a3	d.26.1.1	114	1	1	1	d2f23a2(d.26.1.2)	d2f23a2(d.26.1.2)	d2cssa1(b.36.1.1)	d1g9oa_(b.36.1.1)	d2tpta3(d.41.3.1)	d1jdqa_(d.68.3.3)
d1t16a_	f.4.3.4	428	1	1	1	d1a0tp_(f.4.3.2)	d1a0tp_(f.4.3.2)	d1ejda_(d.68.2.2)	d1g6sa_(d.68.2.2)	d1rf6a_(d.68.2.2)	d2b3ya1(c.8.2.1)
d1ttea1	a.5.2.1	56	5	0	1	d1msza_(d.68.7.1)	d1v92a_(a.5.2.3)	d1xb2b1(a.5.2.2)	d1oaia_(a.5.2.3)	d1wvec1(a.3.1.1)	d1msza_(d.68.7.1)
d1u08a_	c.67.1.1	383	8	1	1	d1c7na_(c.67.1.3)	d1m32a_(c.67.1.3)	d1b9ha_(c.67.1.4)	d1c7na_(c.67.1.3)	d1cl1a_(c.67.1.3)	d1iuga_(c.67.1.3)
d1u79a_	d.26.1.1	126	1	0	0	d1g9oa_(b.36.1.1)	d1g9oa_(b.36.1.1)	d1uewa_(b.36.1.1)	d2cssa1(b.36.1.1)	d1rzxa_(b.36.1.1)	d1jdqa_(d.68.3.3)
d1v72a1	c.67.1.1	346	8	1	1	d1m32a_(c.67.1.3)	d1m32a_(c.67.1.3)	d1c7na_(c.67.1.3)	d1o69a_(c.67.1.4)	d1iuga_(c.67.1.3)	d1ohwa_(c.67.1.4)
d1v92a_	a.5.2.3	47	18	1	1	d1xb2b1(a.5.2.2)	d1wgna_(a.5.2.1)	d2bwba1(a.5.2.1)	d1wiva_(a.5.2.1)	d1xb2b1(a.5.2.2)	d2dnaa1(a.5.2.1)
d1vcoa2	c.37.1.10	273	5	0	1	d1v72a1(c.67.1.1)	d3dhwc1(c.37.1.12)	d1l2ta_(c.37.1.12)	d1rd5a_(c.1.2.4)	d1v72a1(c.67.1.1)	d1ps9a1(c.1.4.1)
d1vdla_	a.5.2.1	81	5	1	1	d1xb2b1(a.5.2.2)	d1otra_(a.5.2.4)	d1v92a_(a.5.2.3)	d1oaia_(a.5.2.3)	d1xb2b1(a.5.2.2)	d1ctja_(a.3.1.1)
d1vega_	a.5.2.1	84	5	1	1	d1v92a_(a.5.2.3)	d1v92a_(a.5.2.3)	d1xb2b1(a.5.2.2)	d1rgwa_(b.36.1.1)	d1jo0a_(d.68.4.1)	d1rzxa_(b.36.1.1)
d1veka_	a.5.2.1	85	5	1	1	d1xb2b1(a.5.2.2)	d1xb2b1(a.5.2.2)	d1otra_(a.5.2.4)	d1v92a_(a.5.2.3)	d1oaia_(a.5.2.3)	d1jdqa_(d.68.3.3)
d1wgna_	a.5.2.1	64	5	1	1	d1oaia_(a.5.2.3)	d1mn3a_(a.5.2.4)	d1v92a_(a.5.2.3)	d1oaia_(a.5.2.3)	d1xb2b1(a.5.2.2)	d1otra_(a.5.2.4)
d1wiva_	a.5.2.1	74	5	1	1	d1xb2b1(a.5.2.2)	d1oaia_(a.5.2.3)	d1otra_(a.5.2.4)	d1xb2b1(a.5.2.2)	d1v92a_(a.5.2.3)	d1wvec1(a.3.1.1)
d1wj7a1	a.5.2.1	92	5	1	1	d1oaia_(a.5.2.3)	d1otra_(a.5.2.4)	d1xb2b1(a.5.2.2)	d1oaia_(a.5.2.3)	d1v92a_(a.5.2.3)	d1tp5a1(b.36.1.1)
d1xb2b1	a.5.2.2	57	19	1	1	d1oaia_(a.5.2.3)	d1oaia_(a.5.2.3)	d1vdla_(a.5.2.1)	d2bwba1(a.5.2.1)	d1v92a_(a.5.2.3)	d2dnaa1(a.5.2.1)
d2b3ya1	c.8.2.1	260	1	0	0	d1i4na_(c.1.2.4)	d1wbha1(c.1.10.1)	d1m32a_(c.67.1.3)	d1vc4a_(c.1.2.4)	d1kida_(c.8.5.1)	d1i4na_(c.1.2.4)
d2bwba1	a.5.2.1	45	5	1	1	d1xb2b1(a.5.2.2)	d1xb2b1(a.5.2.2)	d1mn3a_(a.5.2.4)	d1otra_(a.5.2.4)	d1v92a_(a.5.2.3)	d1oaia_(a.5.2.3)
d2cpwa1	a.5.2.1	52	5	1	1	d1xb2b1(a.5.2.2)	d1xb2b1(a.5.2.2)	d1mn3a_(a.5.2.4)	d1v92a_(a.5.2.3)	d1oaia_(a.5.2.3)	d1otra_(a.5.2.4)
d2crna1	a.5.2.1	52	5	1	1	d1xb2b1(a.5.2.2)	d1otra_(a.5.2.4)	d1v92a_(a.5.2.3)	d1xb2b1(a.5.2.2)	d1mn3a_(a.5.2.4)	d1oaia_(a.5.2.3)
d2ctza1	c.67.1.3	422	8	1	1	d1o69a_(c.67.1.4)	d2r5ea1(c.67.1.1)	d1b9ha_(c.67.1.4)	d1m6sa_(c.67.1.1)	d1o69a_(c.67.1.4)	d1v72a1(c.67.1.1)
d2dkla1	a.5.2.1	73	5	1	1	d1xb2b1(a.5.2.2)	d1v92a_(a.5.2.3)	d1otra_(a.5.2.4)	d1xb2b1(a.5.2.2)	d1oaia_(a.5.2.3)	d1mn3a_(a.5.2.4)
d2dnaa1	a.5.2.1	51	5	1	1	d1xb2b1(a.5.2.2)	d1otra_(a.5.2.4)	d1mn3a_(a.5.2.4)	d1v92a_(a.5.2.3)	d1oaia_(a.5.2.3)	d1xb2b1(a.5.2.2)
d2f23a2	d.26.1.2	80	7	1	1	d1hxva_(d.26.1.1)	d1t11a3(d.26.1.1)	d1hxva_(d.26.1.1)	d1rzxa_(b.36.1.1)	d1rgwa_(b.36.1.1)	d1va8a1(b.36.1.1)
d2hi6a1	c.8.2.3	133	2	0	1	d1kbla2(c.8.1.1)	d1l5ja2(c.8.2.1)	d1nxja_(c.8.7.1)	d1kbla2(c.8.1.1)	d1a9xb1(c.8.3.1)	d2d0oa1(c.8.6.1)
d2hoxa1	c.67.1.1	426	8	1	1	d1c7na_(c.67.1.3)	d1c7na_(c.67.1.3)	d1m32a_(c.67.1.3)	d2ctza1(c.67.1.3)	d1iuga_(c.67.1.3)	d1cl1a_(c.67.1.3)
d2k0bx1	a.5.2.1	53	5	1	1	d1oaia_(a.5.2.3)	d1xb2b1(a.5.2.2)	d1otra_(a.5.2.4)	d1v92a_(a.5.2.3)	d1oaia_(a.5.2.3)	d1mn3a_(a.5.2.4)
d2onka1	c.37.1.12	241	4	1	1	d1g6oa_(c.37.1.11)	d1g6oa_(c.37.1.11)	d1nija1(c.37.1.10)	d1byia_(c.37.1.10)	d1to3a_(c.1.10.1)	d1ryph_(d.153.1.4)
d2r5ea1	c.67.1.1	419	8	1	1	d1c7na_(c.67.1.3)	d1iuga_(c.67.1.3)	d1o69a_(c.67.1.4)	d1c7na_(c.67.1.3)	d1b9ha_(c.67.1.4)	d1m32a_(c.67.1.3)
d3dhwc1	c.37.1.12	241	4	1	1	d1g6oa_(c.37.1.11)	d1g6oa_(c.37.1.11)	d1nija1(c.37.1.10)	d1byia_(c.37.1.10)	d1kida_(c.8.5.1)	d1to3a_(c.1.10.1)
d3e46a1	a.5.2.1	43	5	1	1	d1oaia_(a.5.2.3)	d1mn3a_(a.5.2.4)	d1otra_(a.5.2.4)	d1xb2b1(a.5.2.2)	d1v92a_(a.5.2.3)	d1oaia_(a.5.2.3)
**Total Hit**				**44**	**50**						
**Total seq**				**56**	**56**						
**Success Rate**				**78.57%**	**89.29%**						

Fold											
**Name**	**SCOP ID**	**Length**	**Member**	**Rank1 Hit**	**Rank5 Hit**	**Rank1 List**	**Rank5 List**				
d1a9xb1	c.8.3.1	152	11	1	1	d2b0aa1(c.8.8.1)	d2hi6a1(c.8.2.3)	d2b0aa1(c.8.8.1)	d1kbla2(c.8.1.1)	d1q3qa2(c.8.5.2)	d1i4na_(c.1.2.4)
d1brwa3	d.41.3.1	104	1	0	0	d2cssa1(b.36.1.1)	d1g9oa_(b.36.1.1)	d1wi4a1(b.36.1.1)	d1vaea_(b.36.1.1)	d2cssa1(b.36.1.1)	d1tp5a1(b.36.1.1)
d1djqa1	c.1.4.1	341	8	1	1	d1vc4a_(c.1.2.4)	d1rd5a_(c.1.2.4)	d1vc4a_(c.1.2.4)	d1hl2a_(c.1.10.1)	d1cl1a_(c.67.1.3)	d1m6sa_(c.67.1.1)
d1ejda_	d.68.2.2	420	8	0	0	d1m6sa_(c.67.1.1)	d1m6sa_(c.67.1.1)	d1b9ha_(c.67.1.4)	d1iuga_(c.67.1.3)	d1u08a_(c.67.1.1)	d1m32a_(c.67.1.3)
d1eoka_	c.1.8.5	283	9	1	1	d1djqa1(c.1.4.1)	d1djqa1(c.1.4.1)	d2b3ya1(c.8.2.1)	d1t16a_(f.4.3.4)	d1byia_(c.37.1.10)	d1r61a_(c.8.8.1)
d1g6sa_	d.68.2.2	428	8	0	0	d1iuga_(c.67.1.3)	d1m32a_(c.67.1.3)	d2r5ea1(c.67.1.1)	d2ctza1(c.67.1.3)	d1m6sa_(c.67.1.1)	d1iuga_(c.67.1.3)
d1hc7a3	d.68.5.1	75	9	1	1	d1msza_(d.68.7.1)	d1msza_(d.68.7.1)	d2bkya1(d.68.6.1)	d1tp5a1(b.36.1.1)	d1va8a1(b.36.1.1)	d1r6ja_(b.36.1.1)
d1hl2a_	c.1.10.1	296	7	1	1	d1ps9a1(c.1.4.1)	d1rd5a_(c.1.2.4)	d1ps9a1(c.1.4.1)	d1i4na_(c.1.2.4)	d1b9ha_(c.67.1.4)	d1iuga_(c.67.1.3)
d1i4na_	c.1.2.4	252	7	1	1	d1wbha1(c.1.10.1)	d1to3a_(c.1.10.1)	d1wbha1(c.1.10.1)	d1kkoa1(c.1.11.2)	d1hl2a_(c.1.10.1)	d1kida_(c.8.5.1)
d1jdqa_	d.68.3.3	99	9	0	1	d1m5ya3(d.26.1.1)	d1jo0a_(d.68.4.1)	d1eq3a_(d.26.1.1)	d1hxva_(d.26.1.1)	d1m5ya3(d.26.1.1)	d1u79a_(d.26.1.1)
d1jo0a_	d.68.4.1	98	10	1	1	d2bkya1(d.68.6.1)	d1msza_(d.68.7.1)	d2bkya1(d.68.6.1)	d1r6ja_(b.36.1.1)	d1vaea_(b.36.1.1)	d1m5ya3(d.26.1.1)
d1kbla2	c.8.1.1	134	11	1	1	d2hi6a1(c.8.2.3)	d2isba1(c.8.9.1)	d1nxja_(c.8.7.1)	d2hi6a1(c.8.2.3)	d1q3qa2(c.8.5.2)	d1wbha1(c.1.10.1)
d1kida_	c.8.5.1	194	10	0	1	d1nija1(c.37.1.10)	d2b3ya1(c.8.2.1)	d1kbla2(c.8.1.1)	d1nija1(c.37.1.10)	d1l2ta_(c.37.1.12)	d1wbha1(c.1.10.1)
d1kkoa1	c.1.11.2	252	9	1	1	d1to3a_(c.1.10.1)	d1vc4a_(c.1.2.4)	d1to3a_(c.1.10.1)	d1wbha1(c.1.10.1)	d1i4na_(c.1.2.4)	d1byia_(c.37.1.10)
d1l5ja2	c.8.2.1	213	9	0	1	d1wbha1(c.1.10.1)	d1r61a_(c.8.8.1)	d1kbla2(c.8.1.1)	d1wbha1(c.1.10.1)	d1byia_(c.37.1.10)	d1l2ta_(c.37.1.12)
d1msza_	d.68.7.1	63	9	1	1	d1pava_(d.68.3.3)	d1nj8a2(d.68.5.1)	d2bkya1(d.68.6.1)	d1pava_(d.68.3.3)	d1oaia_(a.5.2.3)	d1ttea1(a.5.2.1)
d1nj8a2	d.68.5.1	63	9	0	1	d1m5ya3(d.26.1.1)	d1msza_(d.68.7.1)	d1eq3a_(d.26.1.1)	d2f23a2(d.26.1.2)	d1kbla2(c.8.1.1)	d1m5ya3(d.26.1.1)
d1nxja_	c.8.7.1	157	11	1	1	d1kbla2(c.8.1.1)	d2d0oa1(c.8.6.1)	d1kbla2(c.8.1.1)	d1r61a_(c.8.8.1)	d2isba1(c.8.9.1)	d1byia_(c.37.1.10)
d1pava_	d.68.3.3	79	9	1	1	d1msza_(d.68.7.1)	d1msza_(d.68.7.1)	d1hxva_(d.26.1.1)	d1eq3a_(d.26.1.1)	d2f23a2(d.26.1.2)	d1m5ya3(d.26.1.1)
d1ps9a1	c.1.4.1	331	8	1	1	d1to3a_(c.1.10.1)	d1vc4a_(c.1.2.4)	d1to3a_(c.1.10.1)	d1hl2a_(c.1.10.1)	d1rd5a_(c.1.2.4)	d1b9ha_(c.67.1.4)
d1q3qa2	c.8.5.2	154	10	1	1	d1kbla2(c.8.1.1)	d2isba1(c.8.9.1)	d1a9xb1(c.8.3.1)	d1kbla2(c.8.1.1)	d1wbha1(c.1.10.1)	d1m5ya3(d.26.1.1)
d1r61a_	c.8.8.1	206	10	1	1	d2b3ya1(c.8.2.1)	d1a9xb1(c.8.3.1)	d2b3ya1(c.8.2.1)	d1nxja_(c.8.7.1)	d2isba1(c.8.9.1)	d1to3a_(c.1.10.1)
d1rd5a_	c.1.2.4	262	7	1	1	d1wbha1(c.1.10.1)	d1to3a_(c.1.10.1)	d1hl2a_(c.1.10.1)	d1ps9a1(c.1.4.1)	d1wbha1(c.1.10.1)	d1djqa1(c.1.4.1)
d1rf6a_	d.68.2.2	428	8	0	0	d1m32a_(c.67.1.3)	d1u08a_(c.67.1.1)	d1m32a_(c.67.1.3)	d2ctza1(c.67.1.3)	d1iuga_(c.67.1.3)	d1cl1a_(c.67.1.3)
d1to3a_	c.1.10.1	292	7	1	1	d1rd5a_(c.1.2.4)	d1rd5a_(c.1.2.4)	d1vc4a_(c.1.2.4)	d1i4na_(c.1.2.4)	d1kkoa1(c.1.11.2)	d1m6sa_(c.67.1.1)
d1vc4a_	c.1.2.4	255	7	1	1	d1wbha1(c.1.10.1)	d1to3a_(c.1.10.1)	d1djqa1(c.1.4.1)	d1kkoa1(c.1.11.2)	d1ps9a1(c.1.4.1)	d1wbha1(c.1.10.1)
d1wbha1	c.1.10.1	214	7	1	1	d1vc4a_(c.1.2.4)	d1kkoa1(c.1.11.2)	d1rd5a_(c.1.2.4)	d1vc4a_(c.1.2.4)	d1i4na_(c.1.2.4)	d1byia_(c.37.1.10)
d1whra_	d.68.7.1	125	9	1	1	d1jo0a_(d.68.4.1)	d1jo0a_(d.68.4.1)	d1eq3a_(d.26.1.1)	d1e29a_(a.3.1.1)	d1jvwa_(d.26.1.1)	d2c8sa1(a.3.1.1)
d2b0aa1	c.8.8.1	187	10	1	1	d1a9xb1(c.8.3.1)	d1a9xb1(c.8.3.1)	d2b3ya1(c.8.2.1)	d2d0oa1(c.8.6.1)	d2isba1(c.8.9.1)	d1ryph_(d.153.1.4)
d2b3ya1	c.8.2.1	260	9	0	1	d1i4na_(c.1.2.4)	d1kida_(c.8.5.1)	d1wbha1(c.1.10.1)	d1m32a_(c.67.1.3)	d1vc4a_(c.1.2.4)	d1i4na_(c.1.2.4)
d2bkya1	d.68.6.1	90	10	1	1	d1jo0a_(d.68.4.1)	d1jo0a_(d.68.4.1)	d1hxva_(d.26.1.1)	d1r6ja_(b.36.1.1)	d1m5ya3(d.26.1.1)	d1eq3a_(d.26.1.1)
d2d0oa1	c.8.6.1	163	11	1	1	d2hi6a1(c.8.2.3)	d1nxja_(c.8.7.1)	d2hi6a1(c.8.2.3)	d1q3qa2(c.8.5.2)	d1kida_(c.8.5.1)	d1nija1(c.37.1.10)
d2gyck1	d.41.4.2	132	2	0	0	d1tp5a1(b.36.1.1)	d2cssa1(b.36.1.1)	d1q3qa2(c.8.5.2)	d1q3oa_(b.36.1.1)	d1tp5a1(b.36.1.1)	d1va8a1(b.36.1.1)

d2tpta3	d.41.3.1	106	1	1	1	d1brwa3(d.41.3.1)	d1brwa3(d.41.3.1)	d1tp5a1(b.36.1.1)	d2cssa1(b.36.1.1)	d1kbla2(c.8.1.1)	d1wi4a1(b.36.1.1)
d3dhwc1	c.37.1.12	241	3	1	1	d1l2ta_(c.37.1.12)	d1jj7a_(c.37.1.12)	d2onka1(c.37.1.12)	d1l2ta_(c.37.1.12)	d1g6oa_(c.37.1.11)	d1nija1(c.37.1.10)
d3e46a1	a.5.2.1	43	14	1	1	d1wgna_(a.5.2.1)	d1wgna_(a.5.2.1)	d2dnaa1(a.5.2.1)	d2bwba1(a.5.2.1)	d2cpwa1(a.5.2.1)	d1wiva_(a.5.2.1)
**Total Hit**				**99**	**103**						
**Total seq**				**106**	**106**						
**Success Rate**				**93.40%**	**97.17%**						

d2hi6a1	c.8.2.3	133	9	1	1	d1kbla2(c.8.1.1)	d1nxja_(c.8.7.1)	d1kbla2(c.8.1.1)	d1a9xb1(c.8.3.1)	d2d0oa1(c.8.6.1)	d1u79a_(d.26.1.1)
d2isba1	c.8.9.1	179	11	1	1	d1nxja_(c.8.7.1)	d1q3qa2(c.8.5.2)	d2b0aa1(c.8.8.1)	d1nxja_(c.8.7.1)	d1kbla2(c.8.1.1)	d1l2ta_(c.37.1.12)
d2tpta3	d.41.3.1	106	1	0	0	d1tp5a1(b.36.1.1)	d1tp5a1(b.36.1.1)	d2cssa1(b.36.1.1)	d1kbla2(c.8.1.1)	d1rzxa_(b.36.1.1)	d1wi4a1(b.36.1.1)
**Total Hit**				**25**	**30**						
**Total seq**				**36**	**36**						
**Success Rate**				**69.44%**	**83.33%**						
